# Diversity analysis of 80,000 wheat accessions reveals consequences and opportunities of selection footprints

**DOI:** 10.1038/s41467-020-18404-w

**Published:** 2020-09-11

**Authors:** Carolina Sansaloni, Jorge Franco, Bruno Santos, Lawrence Percival-Alwyn, Sukhwinder Singh, Cesar Petroli, Jaime Campos, Kate Dreher, Thomas Payne, David Marshall, Benjamin Kilian, Iain Milne, Sebastian Raubach, Paul Shaw, Gordon Stephen, Jason Carling, Carolina Saint Pierre, Juan Burgueño, José Crosa, HuiHui Li, Carlos Guzman, Zakaria Kehel, Ahmed Amri, Andrzej Kilian, Peter Wenzl, Cristobal Uauy, Marianne Banziger, Mario Caccamo, Kevin Pixley

**Affiliations:** 1 Genetic Resources Program, International Maize and Wheat Improvement Center (CIMMYT), Carretera México-Veracruz Km. 45 El Batán, Texcoco, C.P. 56237 Mexico; 2 Departamento de Biometria y Estadística, Facultad de agronomía, Universidad de la República, Ruta 3, km 363, Paysandú, C.P. 60000 Uruguay; 3 NIAB, 93 Lawrence Weaver Road, Cambridge, CB3 0LE UK; 4 Geneshifters, 222 Mary Jena Lane, Pullman, WA 99163 USA; 5 Information and Computational Science, The James Hutton Institute, Invergowrie Dundee, DD2 5DA Scotland; 6 Global Crop Diversity Trust, Platz Der Vereinten Nationen 7, Bonn, 53113 Germany; 7 Diversity Arrays Technology, Building 3, Level D, University of Canberra, Monana St., Bruce, ACT 2617 Australia; 8 Departamento de Genética Escuela Técnica Superior de Ingeniería Agronómica y de Montes, Universidad de Córdoba, Córdoba, Spain; 9 Genetic Resouces Program, International Center for Agricultural Research in the Dry Areas (ICARDA), Rabat, Rabat-Salé-Zemmour-Zaër, Morocco; 10 Genetic Resouces Program, International Center for Tropical Agriculture (CIAT), Km 17 Recta Cali-Palmira CP 763537 Apartado Aéreo 6713, Cali, Colombia; 11 John Innes Centre, Norwich Research Park, Norwich, NR4 7UH UK

**Keywords:** Agricultural genetics, Genetic markers, Genetic variation, Plant breeding

## Abstract

Undomesticated wild species, crop wild relatives, and landraces represent sources of variation for wheat improvement to address challenges from climate change and the growing human population. Here, we study 56,342 domesticated hexaploid, 18,946 domesticated tetraploid and 3,903 crop wild relatives in a massive-scale genotyping and diversity analysis. Using DArTseq^TM^ technology, we identify more than 300,000 high-quality SNPs and SilicoDArT markers and align them to three reference maps: the IWGSC RefSeq v1.0 genome assembly, the durum wheat genome assembly (cv. Svevo), and the DArT genetic map. On average, 72% of the markers are uniquely placed on these maps and 50% are linked to genes. The analysis reveals landraces with unexplored diversity and genetic footprints defined by regions under selection. This provides fertile ground to develop wheat varieties of the future by exploring specific gene or chromosome regions and identifying germplasm conserving allelic diversity missing in current breeding programs.

## Abstract (web summary)

Genebanks hold comprehensive collections of wild species, wild relatives, and landraces that are useful for genetic improvement. Here, the authors report the genotype of nearly 80,000 wheat accessions using DArTseq technology to show the less explored genetic diversity.

## Introduction

Wheat is the world’s most widely grown crop, with annual production in excess of 600 million tons across the Americas, Europe, Asia, Australia, and Africa. Wheat provides 20% of the total protein and calories in human nutrition^1^, and supplies ~40% of the dietary intake of essential micronutrients, including zinc, iron, manganese, magnesium, and vitamins B and E for millions of people, who rely on wheat-based diets^2^. It is also an important energy source for farm animals^3^ and is processed for various other uses including fuel^4,5^. Approximately 95% of the global crop is hexaploid bread wheat (*Triticum aestivum* L. *aestivum*, genomic constitution AABBDD), whereas the remaining includes tetraploid durum wheat (*Triticum turgidum* L. *durum*, AABB) and other wheat types of smaller economic importance^6^.

Since its domestication >10,000 years ago, wheat cultivars have increased yields and adapted to a number of different climates and growing conditions^7–10^. This success, however, has resulted in a reduction of the genetic diversity in the elite gene pool^11–14^, This limits the development of new wheat varieties required to sustainably address the demands of the growing world population in a backdrop of climate changes, and abiotic and biotic stresses^15,16^. Wheat germplasm banks conserve ex situ ca. 560,000 accessions, including crop wild relatives (CWR) and landraces (as well as modern cultivars), which harbor untapped genetic diversity that will prove crucial for overcoming these challenges^17–20^. CWR and landraces have evolved mechanisms to survive or thrive in challenging environments through continuous cycles of natural and human selection. However, their resilience and adaptive mechanisms are poorly understood, which limits their use in breeding efforts^12,15^. Among the main challenges limiting the use of this germplasm in breeding is the need to efficiently identify accessions harboring advantageous genetic variants, and linkage drag associated with introgressing these desired genetic variants into elite germplasm^20^. Recent advances in genomics and molecular technologies, however, facilitate the characterization of the genetic diversity in large collections of accessions^17,21,22^, and increasingly provide strategies, e.g., gene editing, that avoid linkage drag^23^. For example, high-throughput genotyping can support the characterization of core subsets of accessions in a germplasm bank that capture most of the genetic diversity of larger germplasm groups^24^, and are a valuable starting point for phenotyping in search of diversity for use in breeding^17,25–28^.

The International Maize and Wheat Improvement Center’s (CIMMYT) germplasm bank is one of the largest wheat (and maize) germplasm providers worldwide, distributing ca. 20,000 packages of wheat seeds per year to ~100 countries. The Seeds of Discovery initiative (SeeD; http://seedsofdiscovery.org/), which aims to facilitate the effective use of genetic diversity of wheat and maize^29^, has characterized nearly 80,000 accessions from two of the world’s largest wheat germplasm banks: (i) CIMMYT, which hosts 140,812 wheat accessions, and (ii) the International Center for Agricultural Research in the Dry Areas (ICARDA), with 43,924 wheat accessions (https://www.genesys-pgr.org/welcome). Genotypic data from these germplasm banks have been used for a wide range of applications^25,28,30–32^.

The main objective of this work is to characterize the global genetic diversity of nearly 80,000 accessions from CIMMYT and ICARDA’s wheat collections, divided in three biological categories: (i) CWR, (ii) domesticated tetraploid taxa (AABB), and (iii) domesticated hexaploid (AABBDD) accessions. Secondly, we aim to understand this diversity and its possible use in breeding by mapping genetic variants (DArTseq-based SNP and SilicoDArT)^33^ to the IWGSC v1.0 reference genome^34^, to the Svevo (durum) tetraploid reference genome^10^, and against the DArT genetic map. Finally, we validate our approach by analyzing previously identified genomic regions associated with key agronomic traits, and we uncover regions or QTL associated with footprints of modern wheat breeding. This provides clues and targets for the wheat research and breeding community.

## Results

### Diversity analysis of nearly 80,000 wheat accessions

We characterized the genetic diversity of 79,191 accessions from the CIMMYT and ICARDA germplasm banks: 56,342 domesticated wheat hexaploids, 18,946 domesticated wheat tetraploids, and 3903 CWR. These wheat accessions originated from 109 countries (passport data in Supplementary Data 1). For this analysis, we used high-quality SNPs and SilicoDArT (PAV (presence/absence variation)) markers generated independently for each of these three collections. As expected, there was a larger proportion (~50%) of common markers between the hexaploid and tetraploid accessions than shared between these and the CWRs (1–6%; Supplementary Fig. 1). After discarding markers with missing rate > 50% and minor allele frequency (MAF) ≤ 0.1%, markers with MAF between 0.1 and 1% constituted 41, 14, and 7% in the hexaploid, tetraploid, and CWRs, respectively (Table 1).

**Table 1:** Number of segregating SNPs and distribution of minor allele frequencies (MAF) across data sets after filtering for missing rate > 50% and MAF ≤ 0.1%.

	Accessions	# of SNPs	SNPs with MAF ≥ 1%	SNPs with MAF ≥ 5%	SilicoDArT presence/absence
Hexaploid	56,342	85,531	50,068	28,078	26,507
Tetraploid	18,946	45,376	38,935	25,084	26,526
CWRs	3903	55,739	51,626	39,907	61,505

We aligned the SNPs and SilicoDArT markers to the hexaploidy wheat RefSeq v1.0 reference genome^34^, and to the durum wheat (Svevo) reference genome^10^ (Supplementary Table 1, Supplementary Fig. 2 for hexaploid, Supplementary Fig. 3 for tetraploid, and Supplementary Fig. 4 for CWR): 70% (66,067) of the markers mapped uniquely for the hexaploids on the RefSeq v1.0, 68% (30,806) and 69% (31,181) for the tetraploids, with the RefSeq v1.0 and Svevo genomes, respectively, and 50% (28,054) for the CWR on the RefSeq v1.0. As expected, most of the SNP markers were in intergenic regions, outside repeats (Supplementary Data 2 and detailed statistics in Supplementary Fig. 5).

The DArT genetic map (v4) (Supplementary Data 3, see “Methods” section) includes 105,122 markers distributed across the 21 hexaploid bread wheat chromosomes, with a mean of 5006 markers per chromosome. Positioning of SNPs and SilicoDArT markers on the DArT genetic map v4 resulted in a homogeneous distribution across all chromosomes (Supplementary Table 2). Totals of 44,501, 24,185, and 18,738 SNP markers have map positions, representing 52.03%, 53.29%, and 33.61% of all SNP markers for the hexaploid, tetraploid, and CWR data sets, respectively. Similarly, 23,571 (89.0%), 18,711 (70.5%), and 19,022 (30.9%) SilicoDArT markers aligned to the genetic map for the hexaploid, tetraploid, and CWR accessions, respectively. Supplementary Fig. 6 shows the complete distribution of the DArT genetic map markers for the hexaploid, tetraploid, and CWR, and the markers in common among pairs and all three groups of accessions.

We used this genotypic data to analyze each group independently. Accessions were classified as either hexaploid, tetraploid, or CWR based on their passport data. An iterative hierarchical clustering approach, based on modified Roger’s distance matrix (MRD), performed a stepwise branching of each group of accessions into subgroups or clusters of germplasm with maximum genetic diversity among and minimum genetic diversity within groups (see “Methods” section). In addition, we performed admixture analysis on a reduced number of markers (MAF > 5%, and linkage disequilibrium thinned), and admixture populations were compared to the MRD cluster approach. Below, we present the results of these analyses for each group.

### Hexaploid analysis reveals unexplored genetic diversity

The 56,342 hexaploid accessions (81% from CIMMYT and 19% from ICARDA germplasm banks) encompass eight domesticated taxa (Supplementary Table 3), in which 99% are *T. aestivum* L. *aestivum*. We assigned these accessions to seven subgroups based on passport data: (1) landraces (40.3%), (2) cultivars, i.e., genotypes that are distinct, uniform, and stable, have been selected for desirable traits, and are widely cultivated (5.9%), (3) elite breeding lines (20.9%), (4) nursery lines, i.e., groups of distinct lines, assembled for comparative description, or evaluation (2.1%), (5) genetic stocks, i.e., typically lines resulting from the intercrossing of distinct species, with described whole chromosome or partial chromatin addition or deletion (1.9%), (6) primary synthetics (stable wheat genetic stocks, botanically named *Aegilotriticum* spp.) resulting from the crossing and chromosome doubling of tetraploid *Triticum* species (usually AABB genome durum wheat) with *Triticum tauschii* (DD genome; 0.25%), and (7) synthetic derivatives lines and their descendants resulting from crosses between a primary synthetic hexaploid and bread wheat (*T. aestivum* L. *aestivum*; 13.8%). We could not classify 8275 (14.7%) of the samples due to the lack of information in their passport data. The accessions originated from 105 countries, with greatest representation from Mexico (31.1%), Iran (8.1%), Turkey (4.4%), China (4.2%), Morocco (2.5%), Pakistan (2.1%), and Afghanistan (2.1%).

**Fig. 1:**
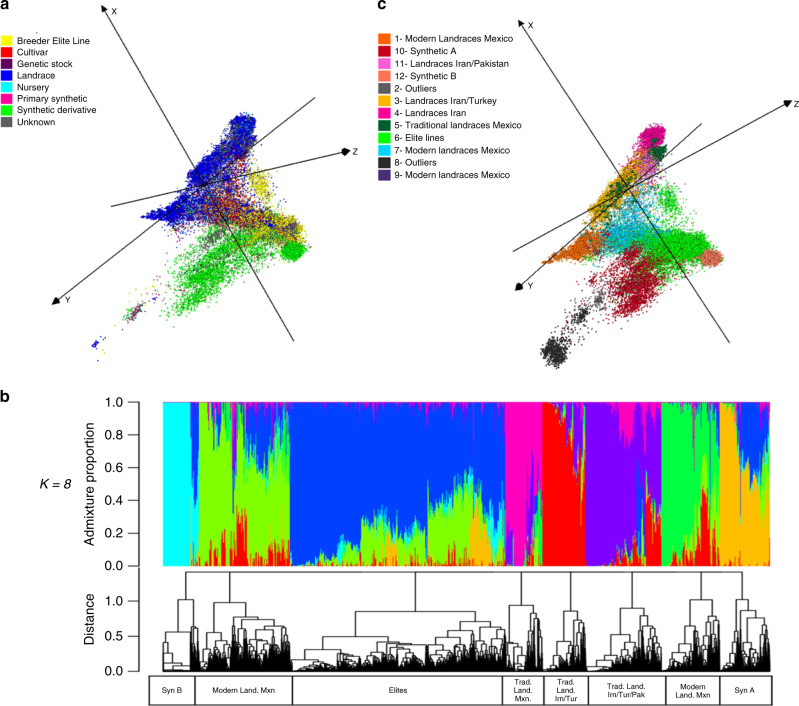
Diversity analysis of domesticated hexaploid accessions. **a** Multidimensional scaling plot visualized in Curlywhirly of 56,342 domesticated hexaploid accession with 66,067 SNP markers differentiated by biological status based on passport information (elite, landraces, cultivar, synthetic, etc.); **b** ADMIXTURE ancestry coefficients (*k* = 6, 12) for a subset of 45,000 samples and dendogram of *k* = 12; **c** the 56,342 hexaploids distributed in 12 clusters based on MRD and clustering analysis. The axes *X*, *Y*, and *Z* correspond to first, second, and third dimensions in MDS.

The multidimensional scaling (MDS) plots (Fig. 1a) illustrate distinct biological groupings within the hexaploid wheats and suggest that a large portion of the genetic diversity in the landraces has not been sampled in modern breeding (for more details see Supplementary Data 4 and Supplementary Movie 1): 70.1% of landraces are at >0.24 MRD distance from the average elite lines (Supplementary Fig. 7).

Admixture analysis at *k* = 8 (Fig. 1b and Supplementary Figs. 8–11) revealed a good fit of the hexaploid diversity into eight groups: (1) traditional landraces from Mexico (Mxn); (2) traditional landraces from Iran (Irn) and Turkey (Tur); (3) traditional landraces from Iran, Turkey, and Pakistan (Pak); (4) and (5) two groups of modern landraces from Mexico; (6) and (7) two groups of synthetic materials, one mainly from primary synthetics (Syn A) and the second with synthetic derivatives crossed with two and up to six elites lines (Syn B); and (8) a group of elite wheat germplasm. Within the large group of elite materials, we found 2260 elite lines, 4400 admixtures of elite with modern landraces, and a few traditional landraces from Iran and Turkey, and synthetics populations. The 2260 elite lines in this group contain in their pedigrees the varieties Kauz, Pastor, or Baviacora, which are progenies of Veery, a high-yielding CIMMYT line from the late 1970s, produced from a spring × winter wheat cross. Other studies have reported genetic separation of these varieties^35,36^, indicating that the 1B/1R translocated segment from rye, present in Veery and derived from Russian cultivar Kavkaz, markedly increases yield^37^.

Analysis of molecular variance (AMOVA) between clusters identified by the diversity analysis (*F*_ST_ values) indicated that grouping into 6 and 12 clusters were the most informative (Supplementary Fig. 12 and Supplementary Movie 2). At the level of 12 clusters, the traditional landraces are divided in four subgroups: traditional Mexican landraces (subgroup 5 in Fig. 1c), landraces from Iran and Pakistan (Lr Irn/Pak, 11), landraces from Iran (Lr Irn, 4), and landraces from Iran and Turkey (Lr Irn/Tur, 3). Two small subgroups (2 and 8), comprising 2.6% of the accessions, were separated from the Syn A group (10); based on their lack of markers on chromosome D, these two small subgroups are likely tetraploids that were originally miss-classified as hexaploids in their passport data and were identified by the analysis as outliers. The modern Mexican landraces were further subdivided, reflecting a greater contribution of elite germplasm to subgroup 7 compared with 1; subgroup 9 is too small to allow interpretation of its uniqueness. Supplementary Figs. 13–15 contains the *F*_ST_ distributions across all chromosomes and Supplementary Figs. 16–21 shows the distribution inside each chromosome. These results are consistent with the MRD analysis and support the idea that a large fraction of genetic variation present in landraces has not been incorporated into elite breeding programs.

### Tetraploid analysis reveals breadth of diversity in durum elites

**Fig. 2:**
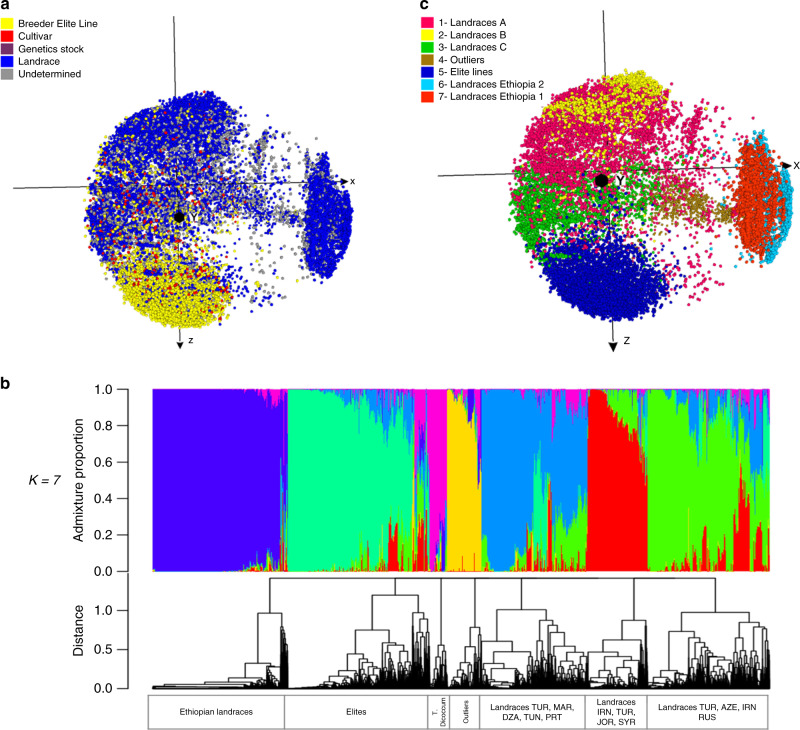
Diversity analysis of domesticated tetraploid accessions. **a** Multidimensional scaling plot visualized in Curlywhirly of 18,946 domesticated tetraploid accessions with 30,806 SNP markers differentiated by biological status based on passport information; **b** ADMIXTURE ancestry coefficients (*k* = 7) and its dendrogram; **c** tetraploids distributed in seven clusters based on MRD.

The 18,946 domesticated tetraploid accessions (20% from CIMMYT and 80% from ICARDA germplasm banks) encompass eight taxa Supplementary Table 4, in which 77.6% are *T. durum*. Based on passport information, we divided the accessions into four groups: landraces (55.8%), elite breeding lines (21.4%), cultivars (3.2%), and genetics stock (0.06%). We could not classify 19.6% of the accessions due to incomplete passport data, but we included those samples in the analysis. The accessions originated from 75 countries, with >50% coming from Ethiopia (18.1%), Turkey (16.7%), Mexico (7.7%), Iran (3.4%), Tunisia (2.8%), Morocco (2.7%), and Syria (2.5%). Analysis of the similarity matrix using MRD with an MDS plot (Fig. 2a) showed that the elite lines are distributed across almost the entire genetic diversity space of the landraces, with the notable exception of a genetically distinct group of several hundred Ethiopian landraces (Supplementary Data 5 and Supplementary Movie 3). Four distinct groups of landraces were identified based on MRD from the allelic frequencies of the group of 4048 elite breeding lines: 817 landraces (7.8%) were genetically close (MRD < 0.2) to the elite lines; 5918 landraces (55.9%) with MRD between 0.20 and 0.30, among which 29% are from Turkey and 10% from Iran; 3483 (32.9%) with MRD between 0.30 and 0.35, mostly (92%) from Ethiopia; and 356 (3.4%) with MRD > 0.35 from the elite lines, 42% of which are from Turkey (Supplementary Fig. 22).

The admixture analysis (*K* = 7) divided the diversity of the tetraploid accessions (Fig. 2b) into: (1) a remarkably distinct group of landraces from Ethiopia; (2) landraces mainly from northern Africa (Morocco, Algeria, and Tunisia) and Portugal; (3) landraces from Turkey, Iran, Jordan, and Syria; and (4) landraces from Turkey, Azerbaijan, Iran, and Russia. The analysis also identified a group (5) of the 3157 elite lines, 2279 (72%) of which were pure to this population, and 879 (28%) were admixtures of elites with landraces. Finally, we identified (6) a small group of accessions from *Triticum diccocum*, and (7) outliers that are potentially hexaploid accessions. All admixture figures from *K* = 2 to *K* = 7 are presented in Supplementary Figs. 23 and 24.

The cluster diversity analysis revealed a high *F*_ST_ value (0.307) at the level of seven clusters (Supplementary Fig. 25, Fig. 2c and Supplementary Movie 4). This analysis highlights the relative uniqueness of the diversity of Ethiopian landraces compared with all other tetraploid materials: tetraploid wheat has been cultivated in Ethiopia for thousands of years, and the area is considered a center of diversity for that species^38^. The *F*_ST_ analysis shows a substantial contribution to genetic diversity from the B genome (Fig. 2c and Supplementary Movie 4). Supplementary Figs. 26 and 27 contains the *F*_ST_ distributions across all chromosomes and Supplementary Figs. 28–30 shows the distribution inside each chromosome. This analysis also identified a group of 1008 accessions with 20% of their SNP markers mapping to D chromosomes, suggesting that they are hexaploids and demonstrating the value of genomic profiling to curate germplasm bank collections.

### CWR analysis characterizes wheat sub-genomes diversity

**Fig. 3:**
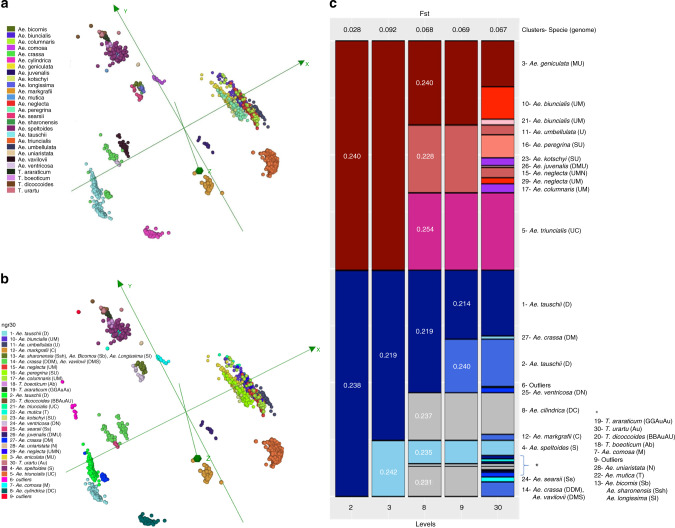
Diversity analysis of CWR accessions. **a** Multidimensional scaling plot visualized in Curlywhirly of 3903 CWR accessions based on Jaccard distance using 61,505 SilicoDArT markers, including the 27 wild relative species; **b** CWR distributed in 30 clusters; **c** Representation of the distribution of 30 groups based on cluster analysis. The graph should be interpreted from left to right. The size of the boxes is proportional to the number of accessions. On the right edge are the numbers of the clusters, species name, and the genome constitution (bold) for each species.

The 3903 accessions of wheat wild relative species (21% from CIMMYT and 79% from ICARDA germplasm banks) include all known 27 wild species from the *Aegilops*–*Triticum* species complex (Fig. 3a, Supplementary Table 5, Supplementary Data 6 and Supplementary Movie 5). *Aegilops* comprises 23 annual species, of which 11 are diploid and 12 are allopolyploid^39–41^. The most represented species in this study were *Aegilops tauschii (*974 accessions)*, Aegilops triuncialis (*661)*, Aegilops geniculata (*401)*, Aegilops cylindrica (*351), and *Aegilops biuncialis* (331). The 27 species comprised 11 genomic constitutions^39^. The accessions originated from 55 countries, with the largest representations from Turkey (17%), Iran (11%), Syria (10%), Lebanon (5%), Jordan (4%), and Greece (4%).

Both the MRD and admixture analysis grouped the accessions by similarity of genomes and then at species level (Fig. 3b, c). In the cluster analysis, the *Aegilops* accessions (1857 accessions of 11 species) containing genome UU at three ploidy levels (red bar in Fig. 3c), including D-genome-containing *Aegilops juvenalis* (DDMMUU), were separated from all other *Aegilops* and *Triticum* species (blue bar in Fig. 3c). Taxa with similar genomes clustered together (e.g., *Aegilops kotschyi* and *Aegilops peregrina*). The *Aegilops* and *Triticum* clusters were further differentiated into section *Cylindropyron* (*Aegilops markgrafii* and *A. cylindrica*) and most species of section *Vertebrata* (*A. tauschii*, *Aegilops crassa*, and *Aegilops ventricosa*) versus sections *Sitopsis* and *Triticum*. Section *Cylindropyron* are differentiated from section *Vertebrata*. The four wild *Triticum* species plus *Aegilops speltoides* (SS) initially grouped together with accessions from section *Sitopsis* (*Aegilops bicornis, Aegilops longissima*, and *Aegilops sharonensis)*. *Aegilops mutica* clustered with section *Comopyrum* (*Aegilops comosa* and *Aegilops uniaristata*) and with *A. uniaristata. Aegilops crassa* (DDDDMM) and *Aegilops vavilovii* (DDMMSS) grouped together. Interestingly, the *Aegilops neglecta* were separated into two groups: (1) 49 potentially tetraploid samples (ssp. *neglecta*, UUMM) and (2) 76 potentially hexaploid (ssp. *recta*, UUMMNN) samples. These subspecies are difficult to distinguish phenotypically according to Van Slageren^42^. It was also interesting that *A. biuncialis* was separated into clusters of 261 and 46 samples. A total of 199 accessions (5.1%) were identified as outliers or potentially misclassified samples, and will be reanalyzed and evaluated to validate or correct their passport data.

### Core set capture global diversity and misclassified accessions

Core germplasm subsets aim to eliminate redundancies and identify representative samples for use in various analyses. We formed core subsets that captured similar genetic diversity to their respective complete collections as indicated by values of expected heterozygosity (He^2^), inbreeding, and Shannon indices for both complete population and core subsets (Supplementary Fig. 31). The core subsets contained 20% of the complete populations, and consisted of 11,235, 3157, and 746 hexaploid, tetraploid, and CWR accessions, respectively (Supplementary Fig. 32).

We conducted a global diversity analysis for wheat by reanalysing the three core subsets together, obtaining 41,717 SilicoDArT and 112,748 SNP markers. The three groups were clearly differentiated using the SilicoDArT markers, and as expected from the results for individual group analyses, we found some outliers among putative tetraploids and hexaploids. We investigated the outliers by calculating the percentage of SilicoDArT markers located on the A, B, and D genomes (Supplementary Fig. 33). A total of 138 (4.4%) putative tetraploids had >10% of markers mapped to the D genome, and 97 of them had >20%. Similarly, among putative hexaploids, 273 (2.4%) had <20% of the markers mapped to the D genome. These observations suggest that 4.4% of tetraploid and 2.4% of hexaploid samples are misclassified in their passport information, a concern that is now being verified by the CIMMYT and ICARDA genebanks. With this analysis, we demonstrate the value of profiling using genomic tools to identified misclassification in germplasm banks.

### Genomic regions under positive selection

**Fig. 4:**
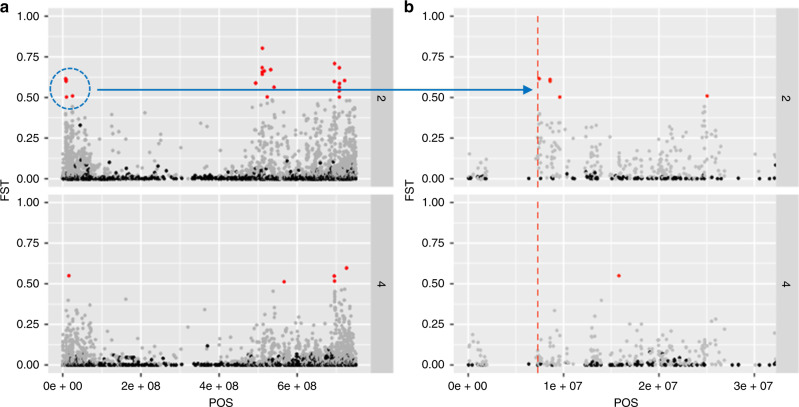
Analysis of *F*_ST_ values on a variant per variant basis across the genome highlights areas of positive selection. **a** *F*_ST_ analysis of the complete chromosome 3A in cluster 2 (upper half) and 4 (lower half of figures); **b** zoom in of chromosome 3A positioning of the preharvest sprouting gene *TaMFT*.

Analysis of *F*_ST_ values on a variant per variant basis across the bread wheat genome highlights areas of positive selection. This is particularly informative when relatively high *F*_ST_ values are considered together with the backgrounds of the groups defined by the clusters (Supplementary Figs. 13–15). For each cluster split, the highest *F*_ST_ values reveal the genomic variants that contributed to the separation of the two subgroups, thereby identifying molecular footprints possibly associated with selective sweeps. We implemented this analysis across the full dataset, noting the genomic regions with high *F*_ST_ values (Supplementary Figs. 34–54). We illustrate the numerous potentially interesting analyses by focusing on two important cluster splits in the hexaploidy group: (1) the first split, which separates the accessions of traditional germplasm from the group that includes most of the elite lines, and (2) the third split, which consolidates the core cluster of elite lines by removing a large set of Mexican landraces (Supplementary Fig. 12). This analysis identified genomic regions that are known to be associated with key agronomic traits, but more importantly, we also uncovered many regions that could help explain the recent history of modern wheat breeding and offer target alleles for future breeding. For example, clusters of loci with high *F*_ST_ within a region of chromosome 3A associated with the well-characterized preharvest sprouting gene (*TaMFT*)^43^, are present in germplasm in cluster 2 (elite lines and Mexican landraces), but are absent in cluster 4 (elite lines and cultivars; Fig. 4).

### GWAS analysis reveals loci associated with GPC and SDS

**Fig. 5:**
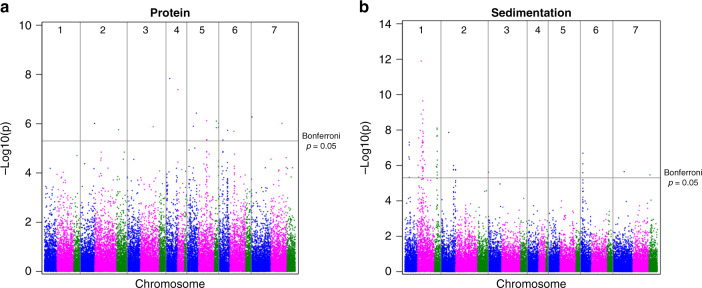
Genome-wide association analysis for wheat quality characters was performed using an iterative usage of fixed and random model circulating probability implemented by R software with correction of kinship, including the first three PCA values as fixed effects. The test was adjusted for multiple comparison in terms of *P*-value cutoff determination and FDR calculation. **a** The 18 genomic regions were associated with GPC on 12 chromosomes, with the highest peaks on 4A and 4B, followed by 5A, 5B, 7A, and 7B. **b** The 19 genomic regions associated with SDS on four chromosomes, 1A, 1B, and 1D, previously reported, and a QTL on 2A.

To conduct association scans with the DArTseq data, we phenotyped 3870 samples for two important traits for processing and end-use quality, grain protein content (GPC) and SDS sedimentation (Fig. 5 and Supplementary Table 6). We found 18 genomic regions associated with GPC on 12 chromosomes, with highest peaks on 4A and 4B, followed by 5A, 5B, 7A, and 7B. Similarly, Kumar et al.^44^ reported major and stable QTL for GPC on chromosomes 5B, 7A, and 7B of an exotic genotype, and indicated that these QTL were independent of grain yield. Such QTL could be useful to enhance GPC through marker-assisted selection (MAS), particularly if they do not compromise yield. Comparison with 49 GPC studies^45^ suggests that QGPC.ndsu.5B (located on 5BS) and QGPC.ndsu.7A.2 (located on 7AL) could be novel QTL, and the exotic germplasm could contribute to the wheat breeding gene pool for increasing GPC.

SDS sedimentation is a common test to determine overall gluten quality. High values on this test are associated with strong gluten (preferred for bread-making), while low values are associated with weak gluten (preferred for pastry products). Here, we report significant QTL for SDS sedimentation and putatively associate them with known storage protein genes. Specifically, high molecular weight glutenins, Glu-A1, Glu-B1, and Glu-D1 (located on the long arms of chromosomes 1A, 1B, and 1D), and low molecular weight glutenins, Glu-A3, Glu-B3, and Glu-D3 (located on the short arms of chromosomes 1A, 1B, and 1D) are candidate genes for the QTL on chromosomes 1A, 1B, and 1D, which had the largest effects on SDS sedimentation in this study. All these glutenin genes are well known, and their variability and effects on processing and end-use quality have been extensively reported^46^.

## Discussion

This diversity analysis for ca. 80,000 accessions from CIMMYT and ICARDA’s seed banks is likely the largest such analysis for any agricultural crop. The analysis of the 56,342 hexaploid accessions highlighted that relatively little of the genetic diversity available in landraces has been used in modern breeding. The largest genetic distance among the hexaploid samples was between elite germplasm and synthetic derivatives, the distinction being driven, as expected, by alleles introduced from *A. tauschii*, the D genome donor. The clustering analysis also identified landraces that have contributed to the genetic pool of modern breeding lines (e.g., landraces in clusters 6 and 7 in Fig. 2c), and landraces that host unexplored alleles/diversity (e.g., landraces from clusters 3, 4, 5, and 11), both presenting fertile ground for exploration and application in breeding programs.

Our diversity clustering analyses reveal genetic footprints defined by regions under selection. We describe the example of preharvest sprouting among hexaploids, for which genetic variants within the *TaMFT* gene (Chromosome 3A) are absent in the cluster of elite lines and cultivars, descendant from precursor cluster 2 that contains these variants. We envision numerous genetic studies that researchers may wish to conduct using the 231 *F*_ST_ chromosome profiles (2–12 cluster on 21 chromosomes) available in Supplementary Figs. 34–53. These data can be used, for example, to explore selective sweeps for any specific gene or chromosome region, analyze footprints defining divergence of landraces from distinct ecologies, or identify germplasm groups conserving allelic diversity missing in current breeding programs.

The analysis of 18,946 tetraploid accessions showed, and in contrast to the hexaploids, that much of the total genetic diversity is represented or explored by the elite durum accessions. There is, however, a large subset of accessions collected in Ethiopia that forms an isolated cluster, and whose genetic content is distinct from and largely unexplored by the elite materials. Analysis of the 3903 CWR highlighted the strong differentiation of sections, followed by species with similar genome constitution, and accurately identified subspecies ploidy level.

We anchored the genomic data to three genomic resources: the latest hexaploid and tetraploid wheat reference genomes and the DArT genetic map, to obtain both physical and genetic positions for the markers capturing the genetic diversity present in the hexaploid and tetraploid wheat accessions, and the CWRs. All the genotypic (DArTSeq) data, and two tools, the DArT genetic map, and CurlyWhirly, are available for use by the research community (see “Methods” section). The DArT genetic map enables confident localization of markers to genome-specific regions, avoiding the common problem in wheat (and other polyploids) of confounding effects among homologous chromosomal regions. CurlyWhirly is valuable for visualizing complex diversity data, e.g., Figs. 1–3. These resources should be useful in gene discovery, cloning, marker development, genomic prediction or selection (GS), MAS, genome-wide association studies (GWAS), and other applications.

These massive-scale genotypic data have already been used in several studies focused on enhancing the use of genetic diversity in wheat breeding. Singh et al.^28^ used DArTseq genotypic and multi-environment phenotypic data to demonstrate positive contributions of exotic germplasm to lines derived from crosses of exotics with CIMMYT’s best elite lines. Genomic-based prediction using 8416 Mexican and 2403 Iranian landraces from CIMMYT’s germplasm bank estimated prediction accuracies from 0.41 to 0.65 for Mexican, and from 0.18 to 0.65 for Iranian landraces^31^. Saint Pierre et al.^32^ characterized 803 spring wheat lines, including elite germplasm and diverse accessions, to develop models for genomic prediction of phenology traits and grain yield, and to predict performance of lines in environments where the lines were not tested. Sehgal et al.^30^ selected 200 diverse gene bank accessions out of 1423 spring bread wheat accessions for use in pre-breeding and allele mining for candidate genes for drought and heat stress tolerance. Finally, Sehgal et al.^47^ described efforts to identify genomic regions with stable expression and their epistatic interactions for grain yield and yield stability in a large panel of elite wheat under multiple environments via a genome-wide association mapping approach. These multiple studies exemplify the value of this germplasm that is now easier to utilize and exploit, thanks to the resources generated in the present study.

Rapid human population growth, climate change, and the need to balance increasing agricultural production with increased environmental sustainability make it necessary to optimize the use of available resources. Native allelic variation for relevant breeding traits is one such resource. The analysis provides a basis for targeted exploration and allele mining activities moving forward. Diversity per se is of limited value for breeding, instead the value lies in the understanding of diversity, and the identification and use of diversity associated with breeder relevant traits. There are a number of paradigms currently in use to better understand and identify breeder relevant diversity. Before the advent of widespread genomic characterization core collections were proposed as a model for mining representations of general diversity, these have evolved to use genomic data in their definition as more widespread characterization has become available^48–50^. Another approach, reflecting landrace adaptation to local environments, was the FIGS (Focused Identification of Germplasm Strategy)^51^ where passport derived collection site variables were used to identify materials of potential interest for phenotypic evaluation for specific environment-associated traits. More recent analysis has extended and revised these approaches to incorporate in-depth understanding and application of genomics. In maize, passport data, associated climate variables from collection sites are being used in conjunction with genome-wide fingerprint data to identify alleles from broad germplasm collections associated with breeder relevant parameters^52,53^. Using this information and screening against genomic profiles of existing elite germplasm enables the identification of both previously unhighlighted standing variation of breeding relevance existing within elite germplasm and also breeder relevant diversity that can then be introgressed into breeding pools using appropriate strategies (S. Hearne pers comm). Taking these parallels and moving forward with wheat, there is a clear opportunity to use the understandings derived from comprehensive genomic characterization, together with associated data, to define and implement clear strategies to explore, and use relevant genetic diversity for breeding in a more targeted data-driven manner.

The genomic data and analysis tools made public with this paper can assist wheat researchers to discover and use functional diversity that may be essential for meeting these challenges. The massive scale of the genotypic data, describing nearly 80,000 publicly available germplasm accessions, offer ample scope for further analyses, not only to understand selection footprints and possible genetic diversity bottlenecks in current elite germplasm, but to leverage relevant diversity from the shelves of germplasm vaults into the hands of breeders. The analyses conducted and described herein, plus examples cited from recent literature, demonstrate the value of molecular data to enrich current passport records and drive the transformation of germplasm banks from museums into strategically, effectively used genetic resource centers.

## Methods

### Plant material and DNA isolation

We explored a total of 79,191 wheat samples; 50,053 from the CIMMYT and 29,138 from the ICARDA germplasm banks. The material included landraces, elite breeding lines, cultivars, primary synthetics, synthetic derivatives, genetic stocks, and wheat wild relatives. The complete list of material is provided in Supplementary Data 1. We randomly selected five seeds of each accession number and grew them at CIMMYT greenhouses in Mexico for 2 weeks. We harvested young leaves of a single plant per accession number, froze them at −80 °C, and lyophilized for 24 h. To track and organize such a large number of accessions, we used CIMMYT’s DNA Sample Tracker System, a platform specifically developed to assist in the tracking of samples from seed to DNA. Genomic DNA was extracted in 96-well plate format from lyophilized leaves using a modified cetyltrimethyl-ammonium bromide method^54^. DNA quality and concentration were determined by electrophoresis on 1% agarose gels.

### High-throughput genotyping using the DArTseq™

We use DArTdb (Diversity Arrays Technology’s database and Laboratory Information Management System) to track the DNA samples from wet lab analysis through to genotyping results. We employed a high-throughput genotyping method using DArTseq™ technology^33^ to genotype all samples. In this technology, the allele-calling pipeline does not require a reference genome that offers an unbiased method to assess genetic diversity in a large collection of accessions, as the one we have analyzed. It might not be the most suitable approach for other investigations, in which having a free reference calling or not using a fully repeatable method like a chip or array it could be a disadvantage. But, considering the objectives of this study and the exotic material we are analyzing, we found that DArTseq was the most appropriate genotyping approached to use at the beginning of the SeeD project. We genotyped ~20% of the samples at the DArT laboratory in Australia and 80% at the Genetic Analysis Service for Agriculture (Spanish acronym SAGA) in Mexico. The first step of library preparation is genomic complexity reduction of the samples through a digestion/ligation reaction using a combination of two restriction enzymes, PstI and HpaII. A PstI-compatible adapter include the Illumina flowcell attachment sequence, the sequencing primer (AATGATACGGCGACCACCGAGATCTACACTCTTTCCCTACACGACGCTCTTCCGATCT), and varying length barcode regions. The reverse adapter contain the flowcell attachment region and the HpaII-compatible overhang sequence (CAAGCAGAAGACGGCATACGAGATCGGTCTCGGCATTCCTGCTGAACCGCTCTTCCGATCTCGG). Only fragments containing PstI-HpaII ends are amplified. After the PCR reaction, equimolar amounts of amplification products from each sample of the 96-well microtiter plate were bulked together, purified, and quantified, followed by sequencing of 77 cycles on Illumina Hiseq 2500 (Illumina Inc., San Diego, CA). The sequences were processed using proprietary DArT analytical pipelines. In the primary pipeline, the FASTQ files were first processed to filter poor-quality sequences by applying two filters: (1) a more stringent filter performed on barcode sequences using a Phred quality score of 30 (representing base call accuracy of 99.9% for at least 75% of the bases), and (2) on the rest of the sequence a Phred quality score of 10 (representing base call accuracy of 90% for at least 50% of the bases). The assignment of sequences to specific samples carried in the barcode split step is therefore very reliable. Approximately 2 million sequences per barcode/sample were used for marker discovery, of which 500,000 unique sequences collapsed into FASTQCOL files. The unique sequences were then identified and clustered by sequence similarity at a distance threshold of three base variations, using DArTsoft14 plugin in KDCompute application (http://www.kddart.org/kdcompute.html). The sequence clusters were then parsed into SNP and SilicoDArT markers using a range of metadata parameters derived from the quantity and distribution of each sequence across all samples in the analysis. One of the crucial parameters is balance of allele counts within the locus across the whole population under study, but there are many additional parameters involved in marker selection. The marker parsing algorithm was trained using data generated on DArTseq platform for over 1000 mapping populations across a broad range of species, including several hundred maps generated on wheat populations at three levels of ploidy reported in this paper. The training process enabled clear discrimination between allelic variants and sequence variation due to paralogous sequences, confirmed by segregation of markers in agreement with Mendelian distributions. DArTsoft14 enables additional tuning of the analysis process to specific material/genome, as it offers 43 parameters for sequence selection/filtering, SNP, and SilicoDArT marker selection and reproducibility calculations.

Most accessions were genotyped once, with a single representative plant per accession number. However, 23% (667 CWR, 4,057 tetraploid and 13,466 hexaploids) of the DNA samples were genotyped multiple times as technical replicates, which enabled calculation of reproducibility scores for each candidate marker. Thus, the total number of libraries analyzed was 97,381. The main parameters to select the markers were call rate (the proportion of samples with genotypic score, i.e. not recorded as missing data) with the threshold of 0.5, and average reproducibility (the proportion of technical replicate assay pairs for which the marker score is consistent) at least 0.95. Similar filtering parameters were used for SilicoDArT markers, but with call rate selection >80%.

DArTsoft14 exports two types of markers, SilicoDArT and SNP. SilicoDArT markers represent presence/absence of restriction fragments of a particular sequence in genomic representations. The PAV acronym has been used for these markers in some reports for genotyping-by-sequencing methods. However, as PAV is a commonly used term to describe presence/absence of a section of DNA in the genome, applying the same term to presence/absence of fragments in genomic representations is a source of confusion. SilicoDArTs are extracted from the sequence data using the DArT proprietary algorithm in DArTsoft14 software. SilicoDArTs are genetically dominant markers, analogous to microarray DArTs, but extracted in silico from sequences, hence the name. There are multiple molecular bases for SilicoDArT markers, with the PAV (absence of DNA sequence in the genome) being the least frequent one. SNPs in the recognition site of the restriction enzymes used in complexity reduction are usually the most common cause of SilicoDArT markers. Indels, both in recognition sites and within restriction fragments delineated by those sites, are also a frequent cause for SilicoDArTs. Cytosine methylation polymorphism within restriction enzyme recognition sites (when using methylation sensitive enzymes for complexity reduction) are responsible for varying proportions of SilicoDArTs, with the proportion of SilicoDArTs resulting from this type of molecular mechanism increasing with the genome size, with PstI site methylation being responsible for <10% of DArT markers small genome *Arabidopsis thaliana*^55^. SilicoDArT’s ability to detect methylation variation is very important as it complements the SNP-based genome profiling by providing some insight into epigenetic variation. SilicoDArTs also outperform SNPs in most deep phylogenetics analyses^56^.

SNP markers are identified de novo by comparing the sequences of fragments present in genomic representations (libraries) of samples processed in DArTsoft14. SNP markers are identified and called completely independently of any reference genome. These two elements (de novo calling and independence from the reference genome) make DArTseq SNP markers particularly robust and practically free from ascertainment bias that plagues many other genotyping technologies.

In the diversity analysis of hexaploid and tetraploid groups, we used SNP markers because it has more resolution for similar samples genetic background. Then, for CWR, which include more diverse germplasm with 11 different genome constitutions, we used SilicoDArT markers that perform better than SNP in deep phylogenetic studies.

### Alignment on the genome references

The sequence data of molecular markers generated were processed with the dartR packagev1.0.5 (ref. ^57^) and converted to a SNPRelate CoreArray Genomic Data Structure (GDS)^58^ before exporting to plink bed format.

Nucleotide sequences for the DArT alleles were extracted from the markers sequence file and transformed into FASTA format using an in-house Python script. Adaptor trimming (performed by the same script) finds matches of at least 6 nt between the adaptor and the 3′ of both DArT allele sequences. The resulting FASTA was then used for downstream alignment.

A reciprocal Bowtie2 (ref. ^59^) alignment strategy was used to map the markers to the genomic sequences. Markers were first aligned to the bread wheat IWGSC RefSeq v1.0. for all three biological categories (domesticated hexaploids, domesticated tetraploids, and CWR) and to the durum wheat Svevo reference for the tetraploid group using end-to-end alignment. Unmapped reads were realigned using local alignment. Bowtie2 version 2.3.3.1 was used with default parameters with the following exceptions: –very-sensitive, -p 24 and –k 10, and –very-sensitive-local. Samtools 1.5 (ref. ^60^) was used to convert sam to bam, with the resultant files then merged using samtools merge and their output parsed with a Python script to extract the genomic alignment regions. Bowtie2 with –very-sensitive-local, -L 18, and –p 24 was then run to align these against the original reads. The output of this step was then parsed and compared to the alignments of the previous step. Alignments were parsed using the same Python script and only alignments where both DArT alleles agreed and the alignment was reciprocal were taken forward. For the remaining alignments, the number of mismatches was calculated (ignoring the SNP position) and the alignment with the least number of mismatches was written to a map plink file. A custom Python script was then used to extract the corresponding entries from the initially generated ped plink file using the recently created map file and produce a plink ped file containing genomic coordinates. Plink v1.90b4 was used to convert the ped file to vcf format, the vcf file was annotated using SnpEff^61^, and vcftools was used to generate SNP density coverage statistics.

### Genetic consensus map

The genetic consensus map was built based on 81 individual genetic maps using a DArT markers consensus map software developed by F. Detering (DArT PL unpublished). The software requires the following inputs: (i) a reference map with one linkage group and marker positions per chromosome, and (ii) set of linkage groups from individual populations with marker positions and chromosome assignment for each group. The construction procedure uses the following pseudo-code algorithm: (i) initialize consensus map with the seed map and (ii) for each chromosome, find subset of linkage groups for this chromosome and repeat until subset is empty for each group in subset. The process requires at least three markers in common with the consensus map and correlates the positions of common markers with the consensus map. The group with the highest commonality (correlation × log [number of common markers]) is identified, and if the correlation is larger than 0.5, all markers are joined to the consensus map by linear interpolation. The group is removed from the subset and the process is repeated. Using this iterative process, DArT consensus map version 4 has 105,122 markers and is accessible at https://www.diversityarrays.com/technology-and-resources/genetic-maps/.

### Data cleaning filters

After allele frequency estimation, three filters were applied to the SNP data: (1) selection of markers with proportion of missing values ≤0.50; (2) selection of markers MAF > 0.001; and (3) selection of accessions with proportion of missing values ≤0.50 (for hexaploids and tetraploids) or 0.75 (for wild relatives). In the case of SilicoDArT markers, we instead used a filter to select markers with proportion of missing values ≤0.80.

### Diversity indices

For the genetic diversity analysis we used the allele frequencies of the markers to calculate the expected and observed heterozygosity, inbreeding coefficient, Shannon entropy index, MRD for SNP data, and Jaccard distance for SilicoDArT data.

Expected heterozygosity^62^, or gene diversity^63^, he, is the most used index and, is defined as:

1 $${{he}}_i = 1 - \mathop {\sum}\limits_{j = 1}^2 {\hat p_{ij}^2},$$

for an *i*th diploid marker (locus), and

2 $${{he}} = \frac{1}{L}\mathop {\sum}\limits_{i = 1}^L {{{he}}_i},$$

the average over all loci for the population. The index summarizes genetic variation and it reaches a 0.5 value for diploid loci when the allelic frequencies are equal to 0.5, maximum of diversity. We used 2× he to describe the diversity on a 0 to 1 scale.

Observed heterozygosity, ho_*i*_, is the proportion of heterozygotes at locus *i*th, and it is averaged for the population characterization, ho. Inbreeding and other evolutionary processes affect ho, and comparison with he produces the inbreeding coefficient *f* for a locus:

3 $$f_i = 1 - {{ho}}_i/{{he}}_i,$$

and their average value for a population. The *f* coefficient is the maximum likelihood estimator of inbreeding under Hardy–Weinberg equilibrium^63^.

We used the Shannon diversity index for the *i*th locus:

4 $${\mathrm{sh}}_i = - \mathop {\sum}\limits_{j = 1}^2 {\hat p_{ij}} \, {\mathrm{log}}_2(\hat p_{ij}),$$

and its average value for the population. We used the base-2 logarithm as when the allele frequencies are equal to 0.5 the index value is 1.0, maximum of diversity.

### Genetic distances between individuals

Based on its good mathematical and genetic properties^14^, we selected the MRD to calculate the genetic distance between two individuals *x* and *y*, measured by a set of L SNP markers:

5 $$0 \le {\mathrm{mrd}}_{xy} = \frac{1}{{\sqrt {2L} }}\sqrt {\mathop {\sum }\limits_{i = 1}^L \mathop {\sum }\limits_{j = 1}^2 \left( {\hat p_{ij(x)} - \hat p_{ij(y)}} \right)^2} \le 1.$$

When using SilicoDArT data, we used the Jaccard distance, the ratio of number of agreements (present, present) divided by the total number of loci comparisons excluding the agreement (absent, absent):

6 $$0 \le jd_{xy} = \frac{{n_{pp}}}{{n_{ap} + n_{pa} + n_{pp}}} \le 1,$$

where *n* is the number of agreements or disagreements.

### Graphical representation

We used the MDS statistical method to represent distances (measured into *P* > 3 dimensions) into three dimensions. MDS^64^ is a multivariate method for dimension reduction whose objective function, to be minimized, is the sum of squared differences of the distance between pairs of objects observed in *P* dimensions, minus an estimated distance between the same objects measured into two or three dimensions. We used two algorithms for the MDS analysis: (1) for hexaploids, we used the classical approach in which, to avoid computer memory issues, a matrix basic algorithm^65^ is applied to obtain a solution (60k × 60k distance matrix); and (2) the SMACOF^66^ solution (using a majorization algorithm, minimizing the same objective function as in the classical solution) was used with tetraploids (20k × 20k) and wild relatives (7k × 7k) matrices.

### Cluster analysis and *F*_ST_ computation

We implemented an iterative hierarchical cluster analysis to group the accessions based on genetic diversity. This process starts by considering *n* (the size of the whole population being clustered) clusters. At each iteration, the method joins the nearest (minimum distance) two accessions, or a group and an accession, or two nearest groups to create *n*1, *n*2, …, 2, and 1 final cluster, where genetic distance is maximized between clusters and minimized within clusters. To identify the appropriate number of clusters that best explain the genetic distances in the original collection, we used the changes in pseudo *F* statistic^67^, i.e., the quotient between the variance between clusters divided by the variance within clusters. At each clustering level, we described the genetic diversity using the AMOVA both between and within clusters. For each clustering, we also analyzed the *F*_ST_ values using sliding window and considered their chromosome position to identify putative genomic regions driving the differences in the genetic diversity between and within clusters.

To understand which alleles contribute to the cluster subdivisions in each of the three main groups (CWR, tetraploids, and hexaploids), we computed *F*_ST_ calculation using vcftools v0.1.15 (ref. ^68^). For each cluster subdivision, a list of individuals was generated and calculations were performed, using *F*_ST_ window size of 1 Mb. Results were then loaded into R and plotted using the ggplot2 library. A scatter plot was generated, where *x* is the BIN_START, *y* is the WEIGHT_*F*_ST_ and each chromosome is plotted and colored separately. A geometric smooth was also added with a span = 0.1.

### Building core subsets

Core subsets are a sampling solution to a germplasm bank manager’s challenge of managing big collections. The idea, from Brown^24^, is to look for a 10 or 20% subset of accessions representing the diversity of the whole collection. We followed the strategy proposed by Franco et al. (refs. ^69,70^) to form 20% subsets for genetic analyses, although the method allows formation of smaller or lager subsets according to the needs of the researcher. Briefly, accessions were grouped based on their pair distances (MRDs) and Ward^71^ minimum variance within groups clustering method, before assigning to each cluster a number of genotypes to be sampled that was proportional to the diversity of the cluster, measured as the average value of MRD distances within the group (D-method). Finally, 1000 candidate samples were extracted using stratified random sampling, and the most diverse candidate sample (the sample showing the maximum of average distance) was selected to be the core subset.

### Analysis of molecular variance

The AMOVA analysis was challenging due to the specific markers, even after the filtering process. When comparing hexaploid, tetraploid, and CWR biological groups, we could use a defined number of markers that were informative for those three groups when together, but a different set of markers would be informative when comparing only a pair of them. To address this, we performed different AMOVA analyses: (1) analysis using all informative (polymorphic) markers for all comparisons, (2) analysis using only the informative markers for each specific comparison between pairs, even if a marker was not informative for one of the elements in the comparison, and (3) analysis using only the informative markers for all the groups or categories being compared.

AMOVA analyses were done both per locus (useful to identify different groups) and across all of them (a more general test). We used the AMOVA method as proposed by Nei^72^, and posteriorly described by Berg and Hamrick^73^. *H*_T_ is defined as the genetic diversity in the whole populations, that is, the average value for he for the pooled population, and *H*s is defined as the average value of the within subpopulation genetic diversity values:

7 $$D_{{\mathrm{ST}}} = H_{\mathrm{T}} - H_{\mathrm{S}},$$

is the among subpopulations genetic diversity, and the ratio:

8 $$F_{{\mathrm{ST}}} = D_{{\mathrm{ST}}}/H_{\mathrm{T}},$$

is the proportion of the total diversity distributed among populations. *F*_ST_ does not own an identified statistical distribution, thus a way to determine its significance is using permutation tests, that is, obtaining an estimation of the *F*_ST_ statistic under the null hypothesis (*H*_0_: there are not biological subpopulations), and comparing the real observed value with the 90, 95, or another significant percentile of the null distribution. To obtain the null distribution, 1000 random permutations of genotypes (and their associated allelic frequencies) across biological groups were obtained and their *F*_ST_ values calculated.

### Genome-wide association scans

We subsampled 3870 samples from the diversity analysis to phenotype for GPC and SDS sedimentation at the Wheat Quality laboratory at CIMMYT. We obtained a total of 145,422 SNPs by DArTSeq platform. We filtered to 46,594 SNPs that met the criteria RepAvg ≥ 0.95, MAF ≥ 0.01, missing rate ≤ 0.6, and heterozygosity rate ≤ 0.2. For high-quality 46,594 SNPs dataset, we firstly align it to the wheat reference genome, 31,694 SNPs across the wheat genome were obtained with 11,751, 13,441, and 6502 SNPs distributed on A, B, and D genome separately, secondly, the dataset was compared with consensus map, 3447 SNPs were obtained on A, B, and D genome with 1,234, 1,793, and 420 SNPs, finally, there are 8.62% SNPs couldn’t be mapped. Combined with the two mapped data sets, 35,141 SNPs across the wheat genome were retained for further study. We performed genome-wide association analysis on 35,141 SNPs dataset using an iterative usage of fixed and random model circulating probability unification^74^ implemented by R software with correction of kinship, including the first three PCA values as fixed effects.

### Visualization tools

In this study, we used visualization tools and interactive data repository to handle the large data volumes generated. One of the tools is Flapjack application that provides interactive visualizations of high-throughput genotypic data, allowing for rapid navigation and comparisons between lines, markers, and chromosomes. We exported data sets created in Flapjack or Germinate for visualization using CurlyWhirly (https://ics.hutton.ac.uk/curlywhirly), a tool that we hereby release for use by the research community. CurlyWhirly is a 3D visualization tool that handles large-scale genetic diversity data with plots containing hundreds of thousands of data points. To explore data sets in detail, a comprehensive hierarchical categorization and filtering system allows for fine-grained filtering and selection of data points. Furthermore, CurlyWhirly allows export of screenshots and videos of the data, with associated categorical color keys included. 3D-based multi-select functionality allows selection of data points within a given distance from a defined point, and export of these data points to a file. CurlyWhirly links smoothly with Germinate and Flapjack, providing many options for sub-setting, analysis, and visualization of large data sets.

### Reporting summary

Further information on research design is available in the Nature Research Reporting Summary linked to this article.

## Supplementary information

Supplementary Information

Peer Review

Reporting Summary

Description of Additional Supplementary Files

Supplementary Data 1

Supplementary Data 2

Supplementary Data 3

Supplementary Data 4

Supplementary Data 5

Supplementary Data 6

Supplementary Movie 1

Supplementary Movie 2

Supplementary Movie 3

Supplementary Movie 4

Supplementary Movie 5

## Data Availability

Data supporting the findings of this work is available in the Supplementary Information files and CIMMYT Dataverse repository (https://data.cimmyt.org/dataset.xhtml?persistentId=hdl:11529/10548030). We also stored the same data into Germinate (http://germinate.cimmyt.org/wheat/), which are accessible after a brief registration in the website. All data sets generated and analyzed during the current study are available from the corresponding author upon request. The data sets created in Flapjack or Germinate can be visualized using CurlyWhirly (https://ics.hutton.ac.uk/curlywhirly). A reporting summary for this article is available as a Supplementary Information file. All biological material is available at the CIMMYT and ICARDA germplasm bank upon on-line request at https://www.cimmyt.org/resources/seed-request/ or https://indms.icarda.org/. Records for all germplasm accessions with DOIs included in this study can also be accessed through the Global Information System of the International Treaty on Plant Genetic Resources for Food and Agriculture at https://ssl.fao.org/glis/. Publicly available data used in this manuscript are IWGSC RefSeq v1.0 (https://wheat-urgi.versailles.inra.fr/Seq-Repository/Assemblies) and durum wheat genome (cv. Svevo) (https://www.interomics.eu/durum-wheat-genome). The detailed description of all parameters and settings used in the marker calling pipeline cannot be provided given the proprietary nature of the software, but access to the software can be negotiated and provided free for testing purposes by Diversity Arrays Technology Pty Ltd by contacting dart@diversityarrays.com. The source data underlying Figs. 1–5 are provided as a Source data file.

## Source data

Source Data

## Code availability

The CurlyWhirly visualization tool is described and available for download at https://ics.hutton.ac.uk/curlywhirly/ and the underlying code are available at GitHub (https://github.com/cropgeeks/curlywhirly). The Flapjack analysis and visualization tool is described and available for download at https://ics.hutton.ac.uk/flapjack/ and the underlying code are available at GitHub (https://github.com/cropgeeks/flapjack). Free R software environment used for the diversity and clustering analysis are available at GitHub (https://github.com/jfranco1951/Genetic-Diversity).
