# Supplementary material for: Diversity analysis of 80,000 wheat accessions reveals consequences and opportunities of selection footprints
Source: Nat Commun. 2020 Sep 11;11:4572. doi: 10.1038/s41467-020-18404-w (PMC7486412; doi:10.1038/s41467-020-18404-w)
Supplement: Supplementary file 1 — Supplementary Information [file 41467_2020_18404_MOESM1_ESM.pdf]

Diversity analysis of 80,000 wheat accessions reveals consequences and opportunities of selection footprints

Sansaloni *et al.*

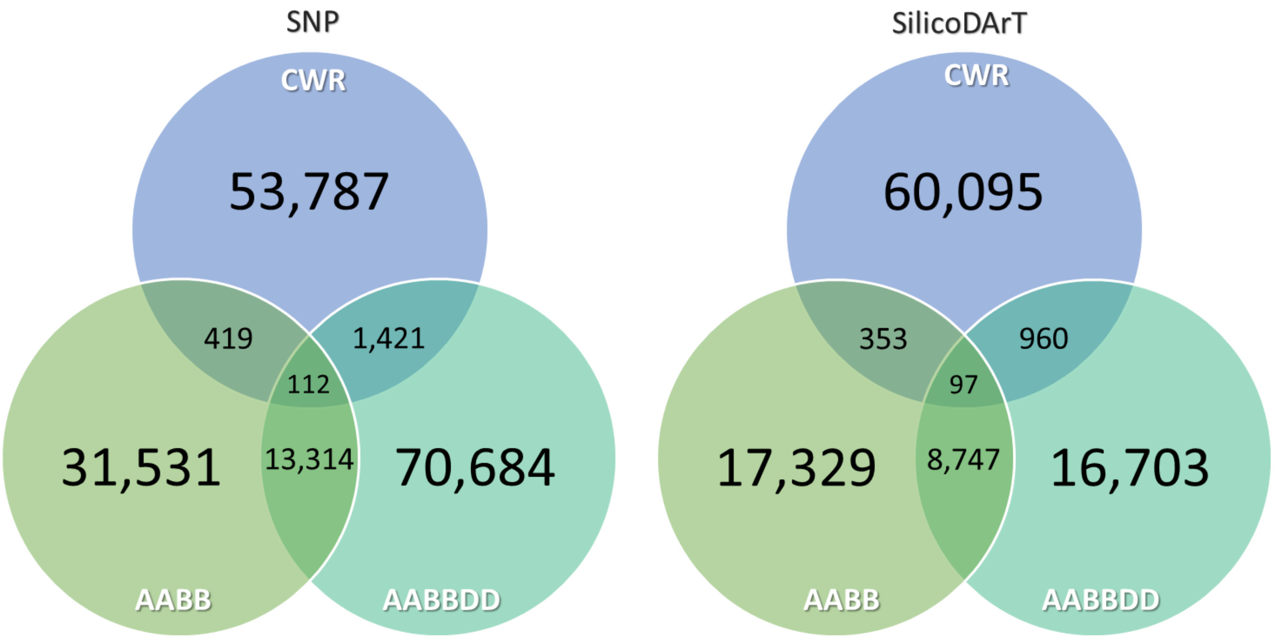

Supplementary Figure 1. Number of SNP and SilicoDArT marker.

Distribution of markers that passed quality filters across the three groups, CWR (2X, 4X and 6X), domesticated tetraploids (AABB) and domesticated hexaploids (AABBDD). The number of common SNP and SilicoDArT markers across data sets is very low between CWR with tetraploid and hexaploid, but higher between tetraploid and hexaploid.

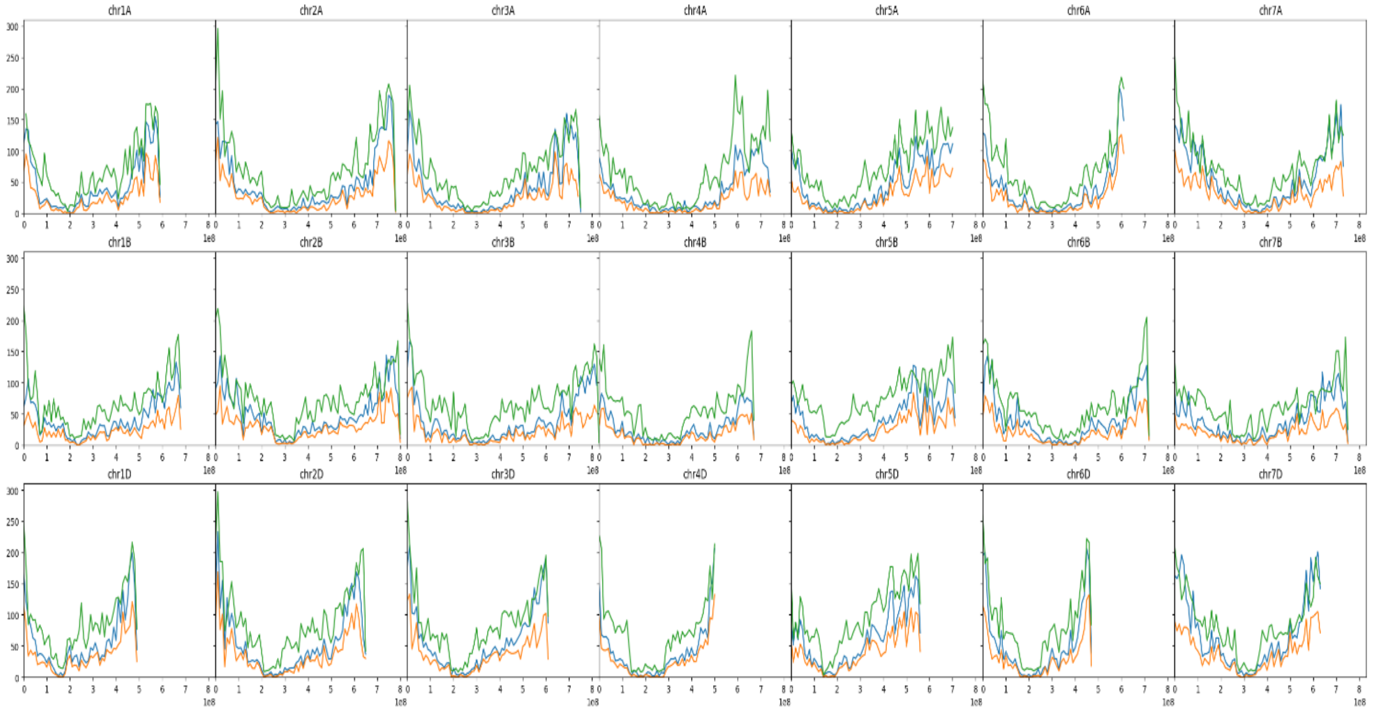

Supplementary Figure 2. Distribution of SNP marker density in hexaploids.

Distribution of 85,531 markers on 21 bread wheat chromosomes divided in 10MB window of the IWGSC RefSeq assembly v1.0. In green can be observed the number of genes; in blue the number of DArTseq markers and in orange the number of markers that overlap one transcript.

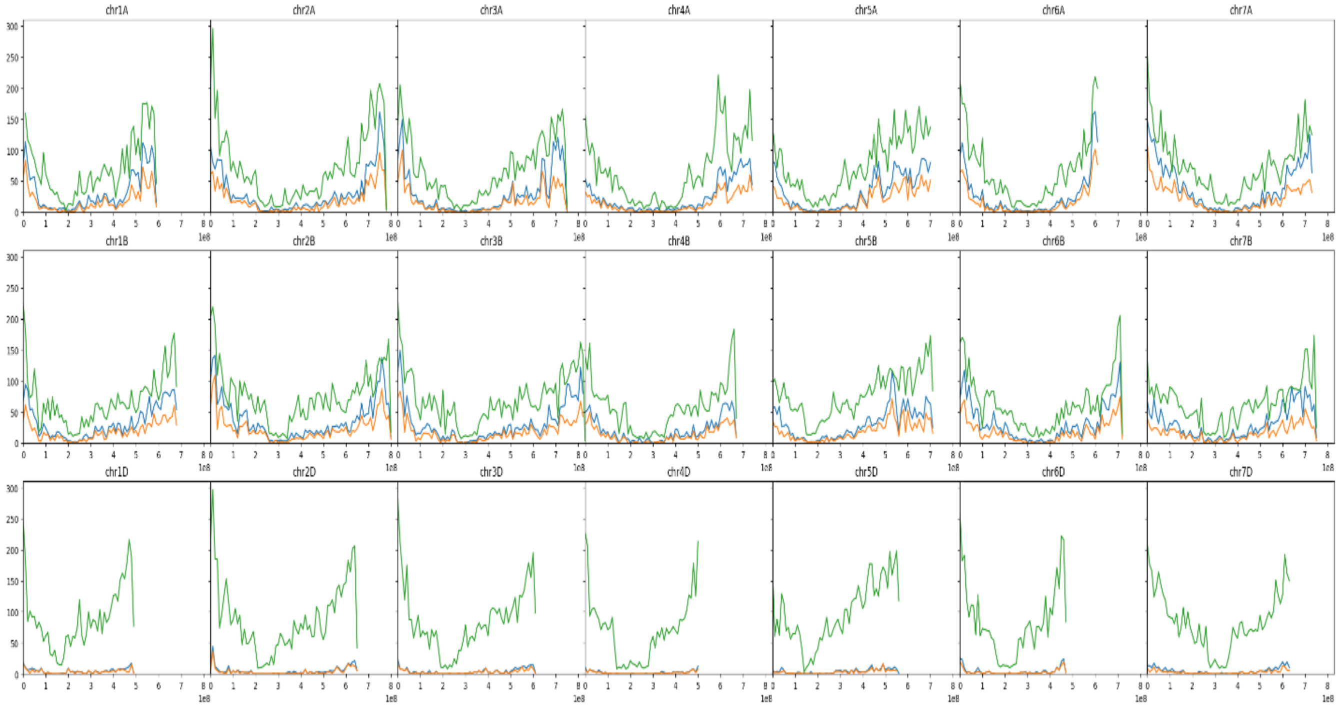

Supplementary Figure 3. Distribution of SNP marker density in tepraploids.

Distribution of 45,376 markers on 21 bread wheat chromosomes divided in 10MB window of the IWGSC RefSeq assembly v1.0. In green can be observed the number of genes; in blue the number of DArTseq markers and in orange the number of markers that overlap one transcript.

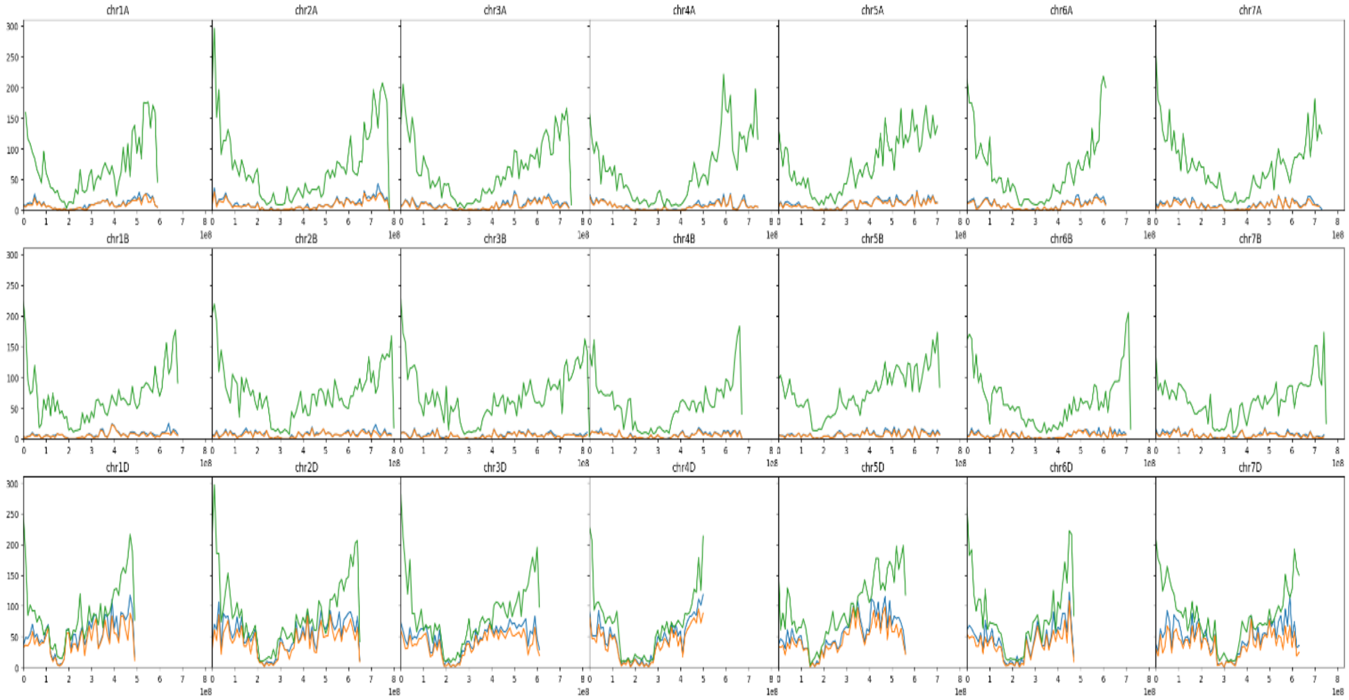

Supplementary Figure 4. Distribution of SNP marker density in CWR.

Distribution of 55,739 markers on 21 bread wheat chromosomes divided in 10MB window of the IWGSC RefSeq assembly v1.0. In green can be observed the number of genes; in blue the number of DArTseq markers and in orange the number of markers that overlap one transcript.

a

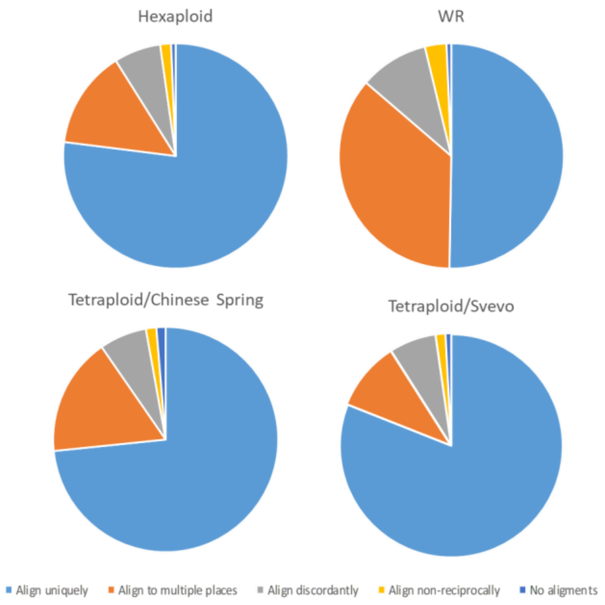

b

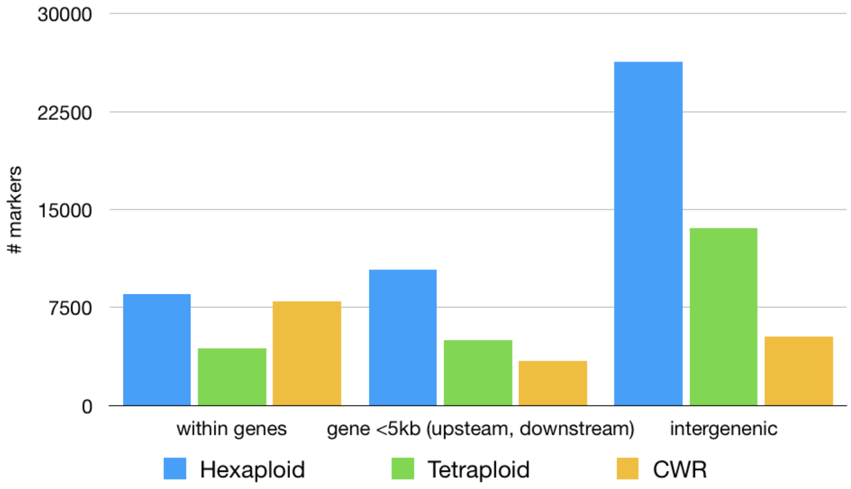

Supplementary Figure 5. Distribution of reciprocal markers alignment on genome references.

a) The markers were distributed in five categories of alignment: align uniquely (the markers mapped only in one location of the genome), mapped to multiple places (the markers mapped two or more location of the genome), mapped discordantly (each allele of the marker aligned to different locations of the genome), mapped non-reciprocally and no alignments. The most representatives are the markers aligned uniquely which represent 66,607 markers (77%) for the hexaploids, 30,806 markers (67.9%) and 31,181 markers (68.7%) for the tetraploids with the RefSeq v1.0 and Svevo genome respectively and 28,054 markers (50.3%) for the CWR. b) Distribution of markers mapping within genes, at <5kb (upstream, downstream) and in intergenic region fr the hexaploidy, tetraploid and CWR groups.

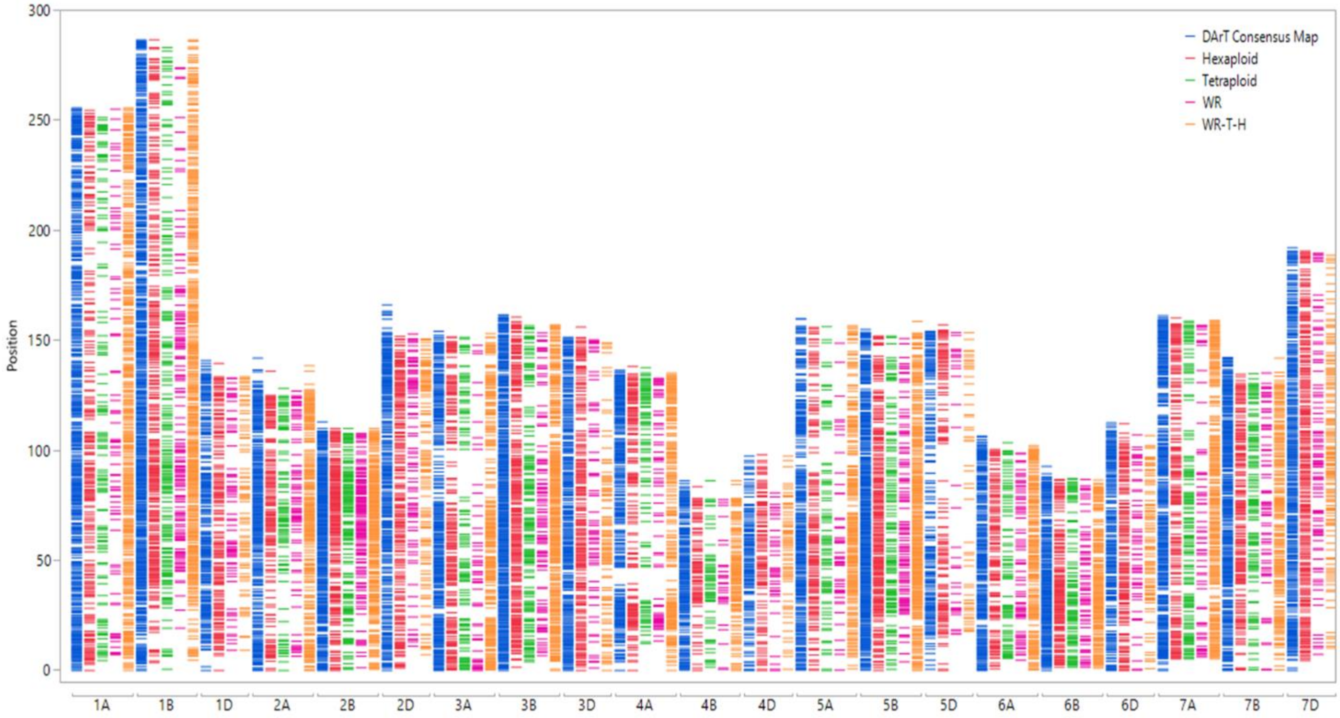

Supplementary Figure 6. Distribution of DArTseq markers on the DArT consensus map.

In blue we present the DArT genetic map (v4) including 105,122 markers distributed across the 21 chromosomes with an average of 5,006 markers per chromosome. We mapped a total of 44,501, 24,185 and 18,738 SNP markers representing 52.03%, 53.29% and 33.61% of the total numbers of SNP markers of the hexaploid (red), tetraploid (green) and wild relative (WR; pink) data sets respectively. Common markers shared among more than one of the three groups are shown in orange.

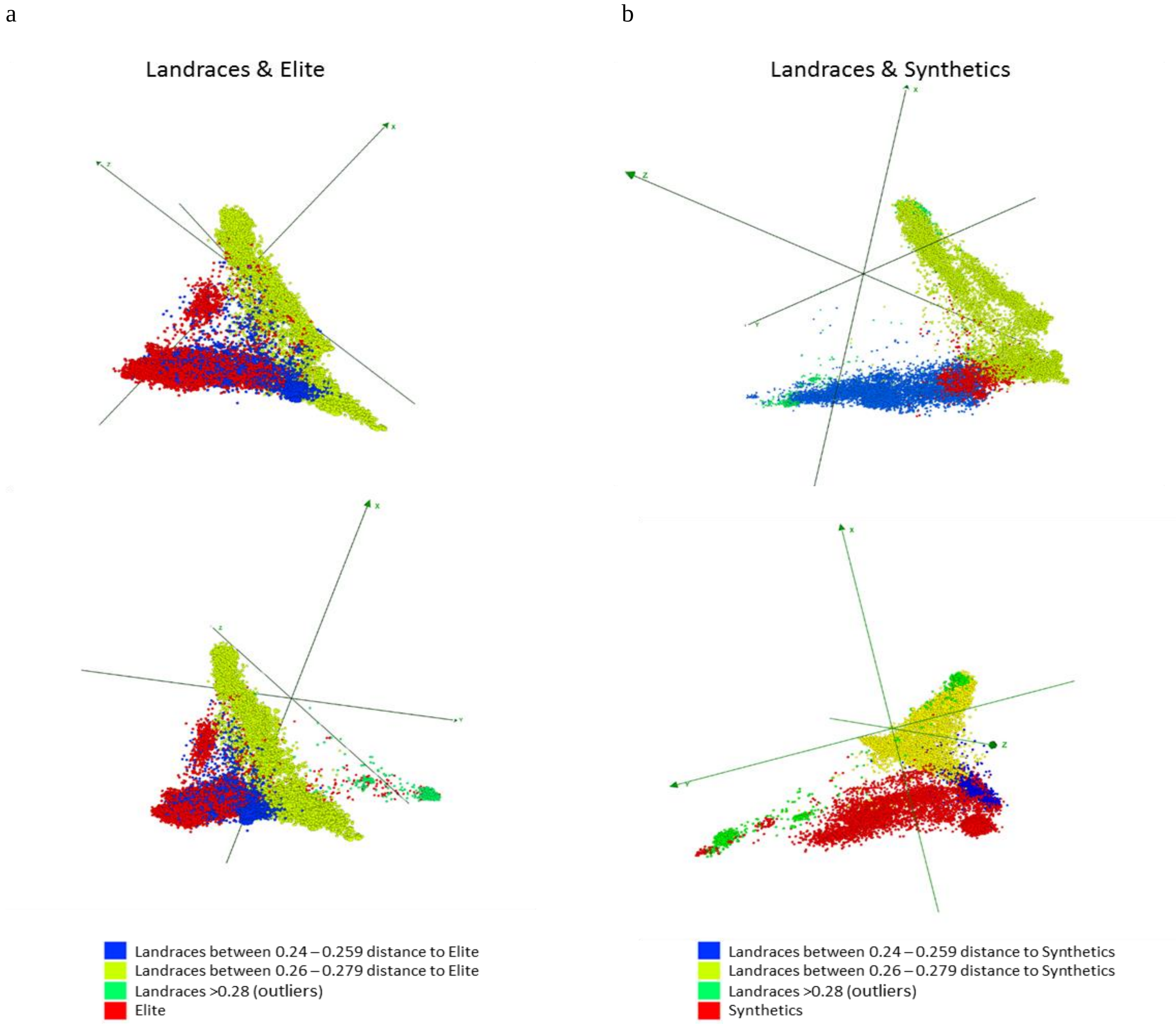

Supplementary Figure 7. MRD between landraces and Elites/Synthetics in hexaploid.

a) The Modified Roger Distance (MRD) between each of the 22,698 landraces and the allelic frequencies for the group of 11,792 breeding elite lines shows three distinct groups, 4,742 landraces (20.9%) genetically very close to breeding elite lines with a genetic distance between 0.240 and less than 0.260, 17,311 landraces (76.3%) between 0.260 and less than 0.280 and 645 (2.84%) more than 0.28 which represent the outliers identified as tetraploid. b) Similar analysis comparing landraces with the group of synthetic accessions revealed 1,621 landraces (7.14%) genetically very close to synthetics with MRD between 0.240 and less than 0.260, 19,284 landraces (84.95%) with MRD between 0.260 less than 0.280, and 3) 1,793 (7.90%) with MRD greater than 0.280. In both cases, it can be observed two angles of the same image.

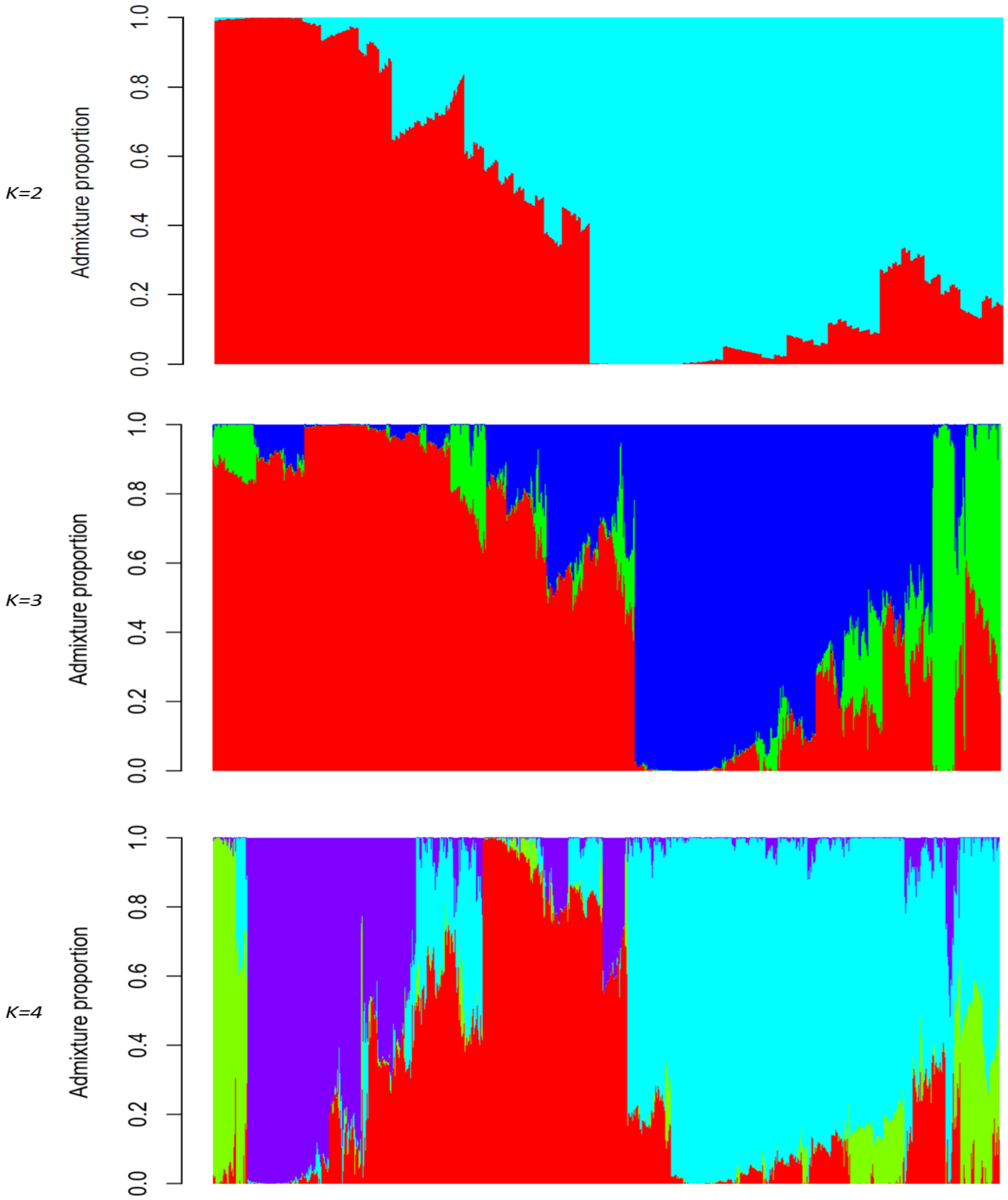

Supplementary Figure 8. ADMIXTURE analysis in hexaploid.

ADMIXTURE ancestry coefficients of a subset of 45,000 hexaploid samples in K=2, K=3 and k=4.

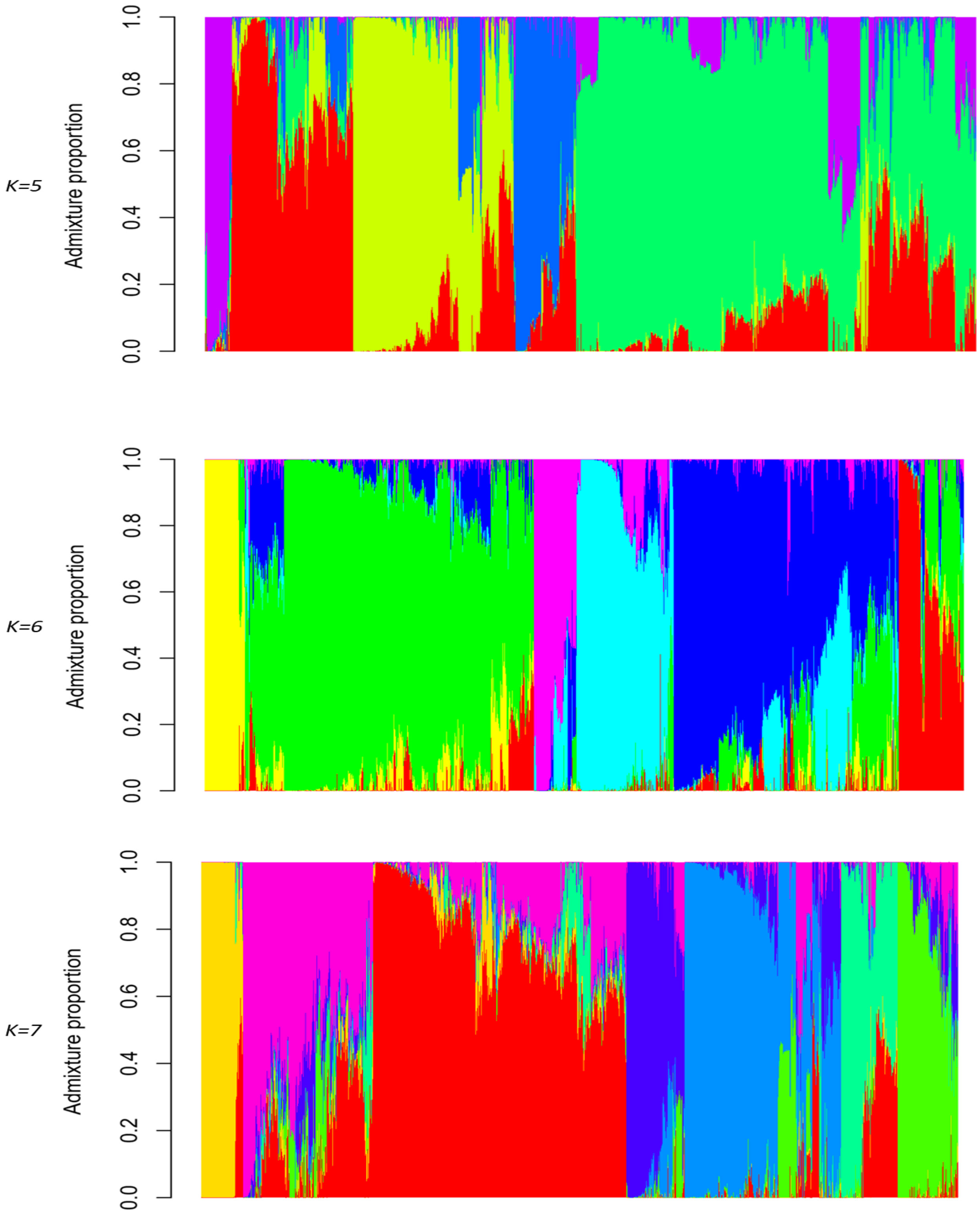

Supplementary Figure 9. ADMIXTURE analysis in hexaploid.

ADMIXTURE ancestry coefficients of a subset of 45,000 hexaploid samples in K=5, K=6 and k=7.

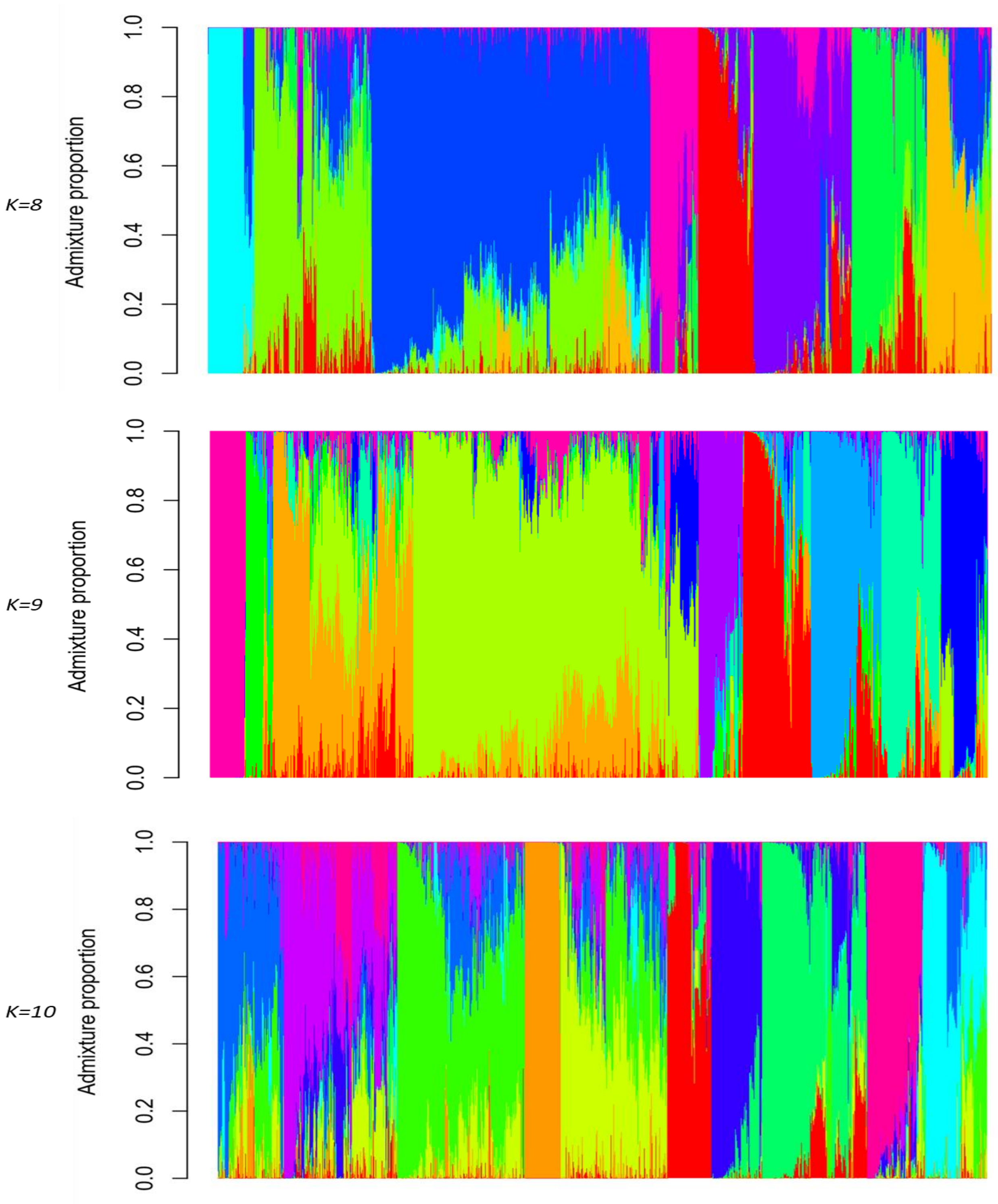

Supplementary Figure 10. ADMIXTURE analysis in hexaploid.

ADMIXTURE ancestry coefficients of a subset of 45,000 hexaploid samples in K=8, K=9 and k=10.

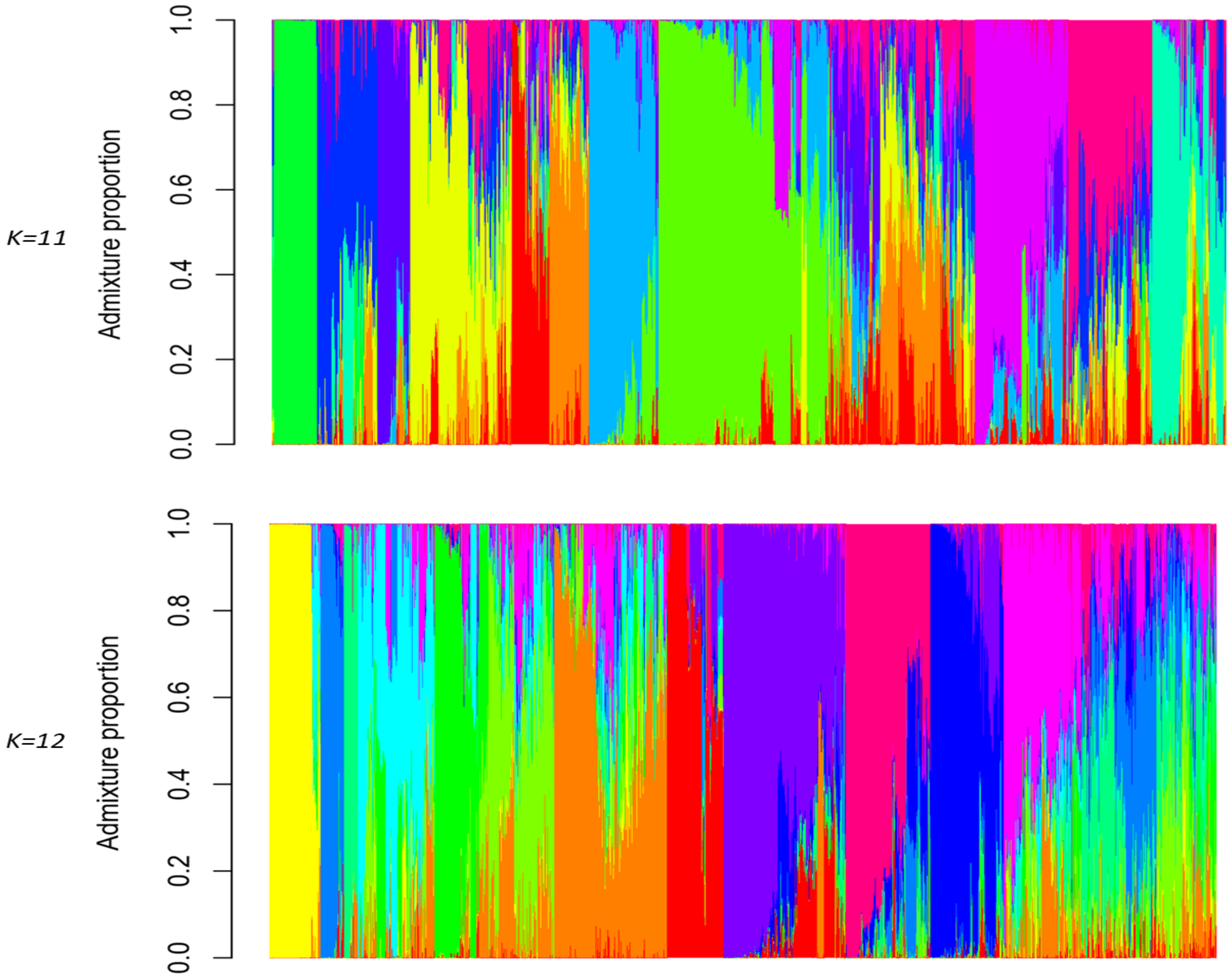

Supplementary Figure 11. ADMIXTURE analysis in hexaploid.

ADMIXTURE ancestry coefficients of a subset of 45,000 hexaploid samples in K=11 and k=12.

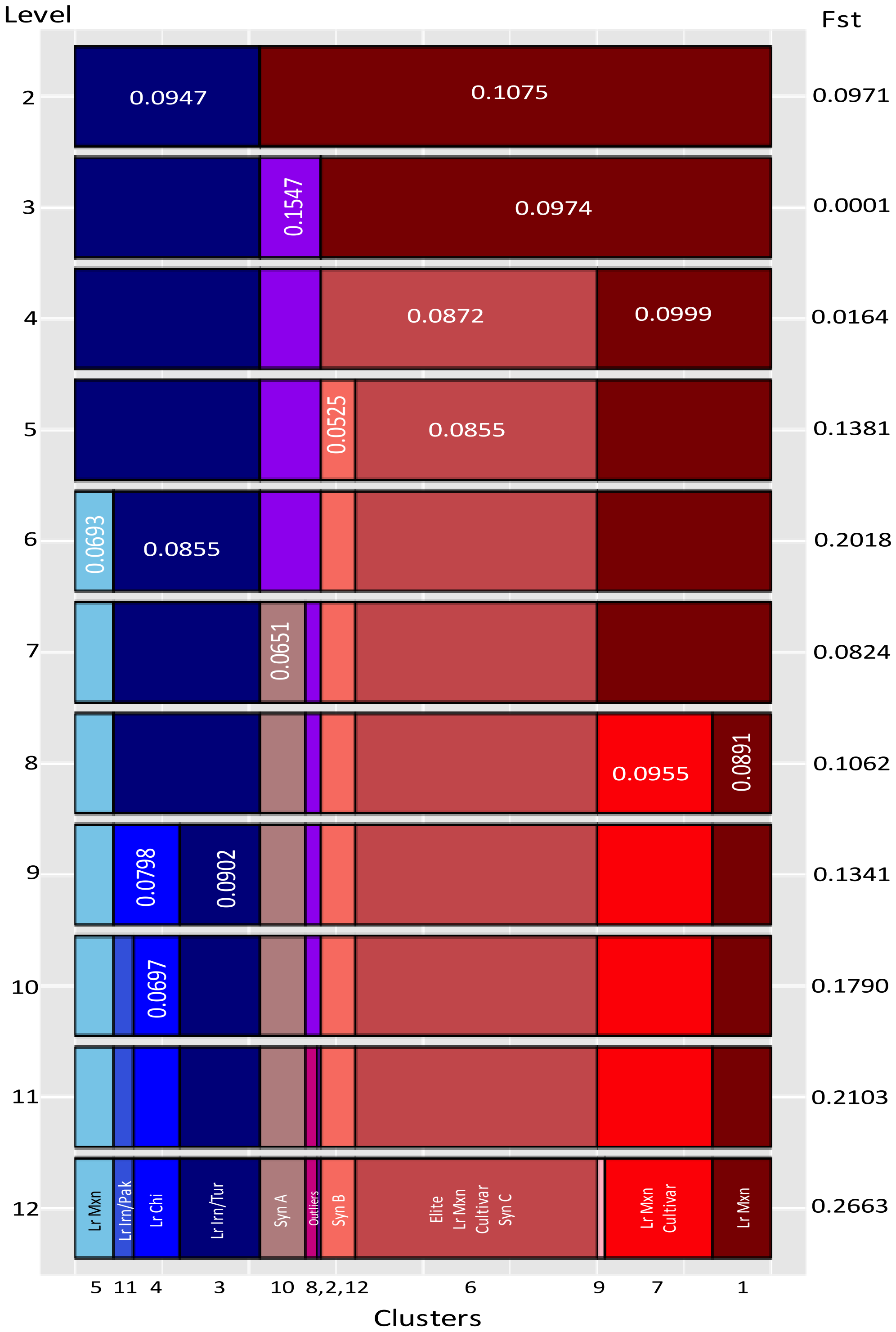

Supplementary Figure 12. Representation of 12 groups based on clusters analysis.

The size of the boxes is proportional to the number of accessions. In the right side are the Fixation index (Fst) values and inside the boxes the expected heterozygosity (He) of each group division and in the bottom the 12 clusters are identified with a brief description and the number which corresponds to figure 1B. Left numbers are the number of levels.

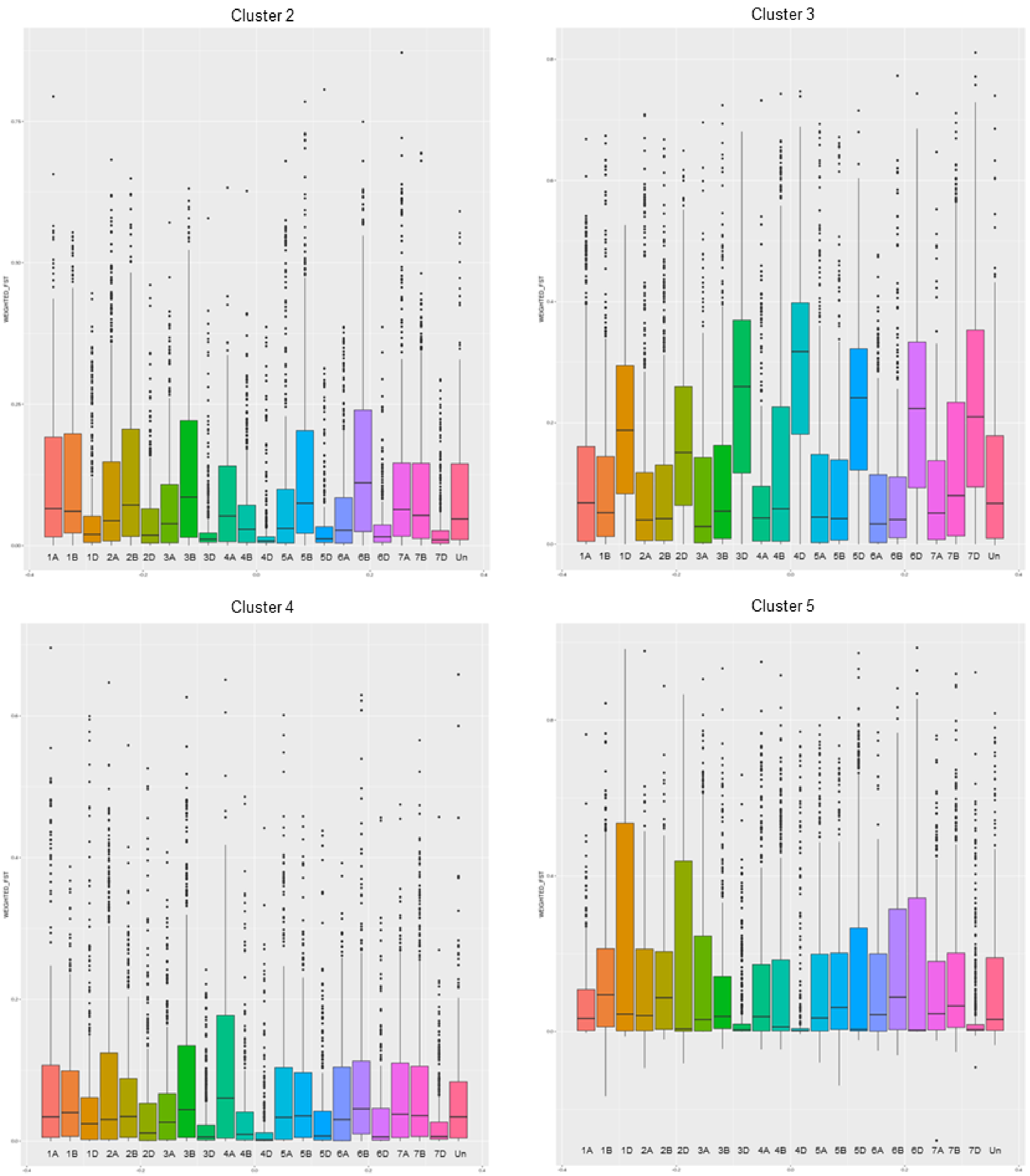

Supplementary Figure 13. Fst analysis per chromosome in hexaploid group.

Fst distributions across all chromosomes in the hexaploid group on cluster division 2, 3, 4 and 5.

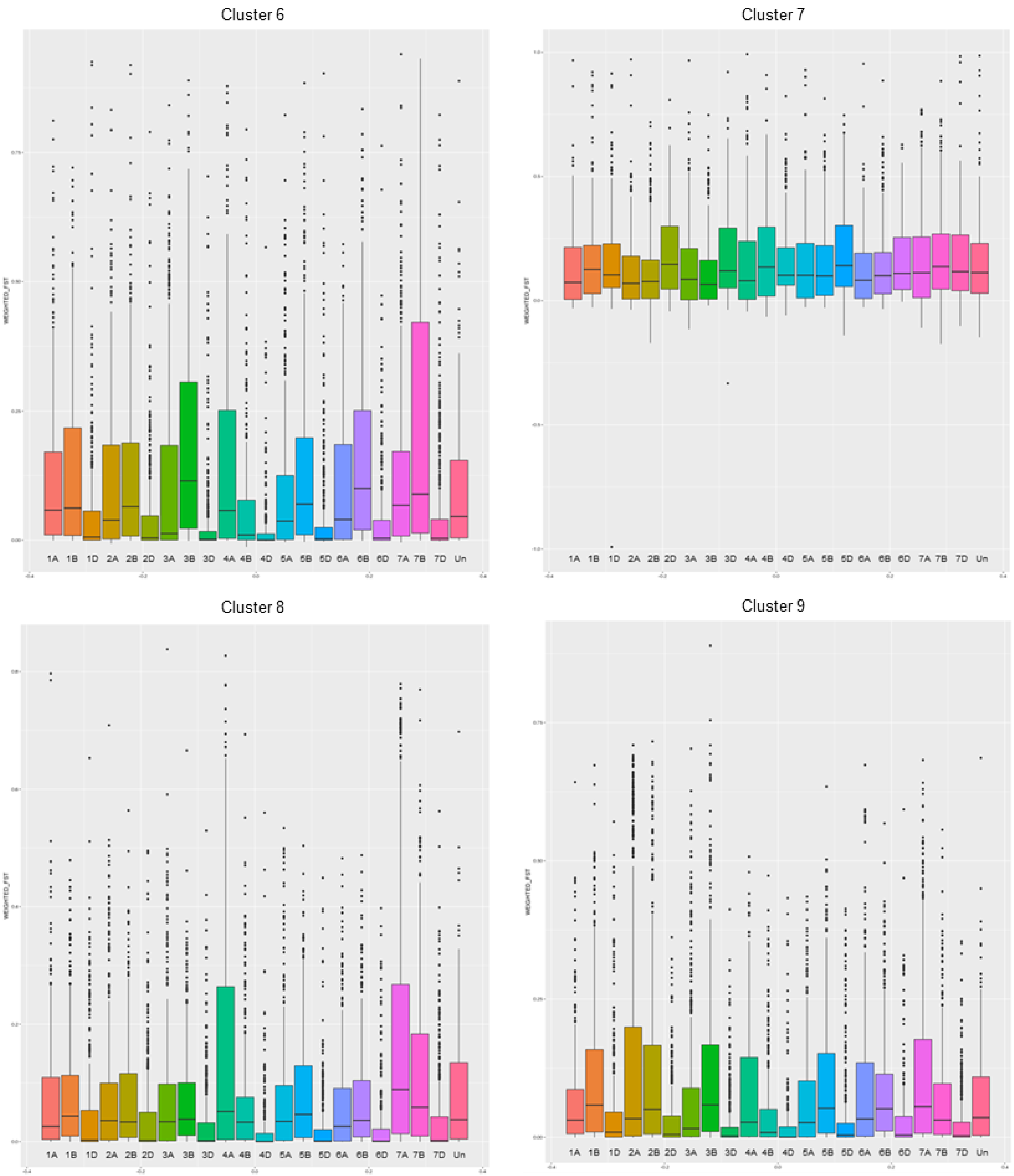

Supplementary Figure 14. Fst analysis per chromosome in hexaploid group.

Fst distributions across all chromosomes in the hexaploid group on cluster division 6, 7, 8 and 9.

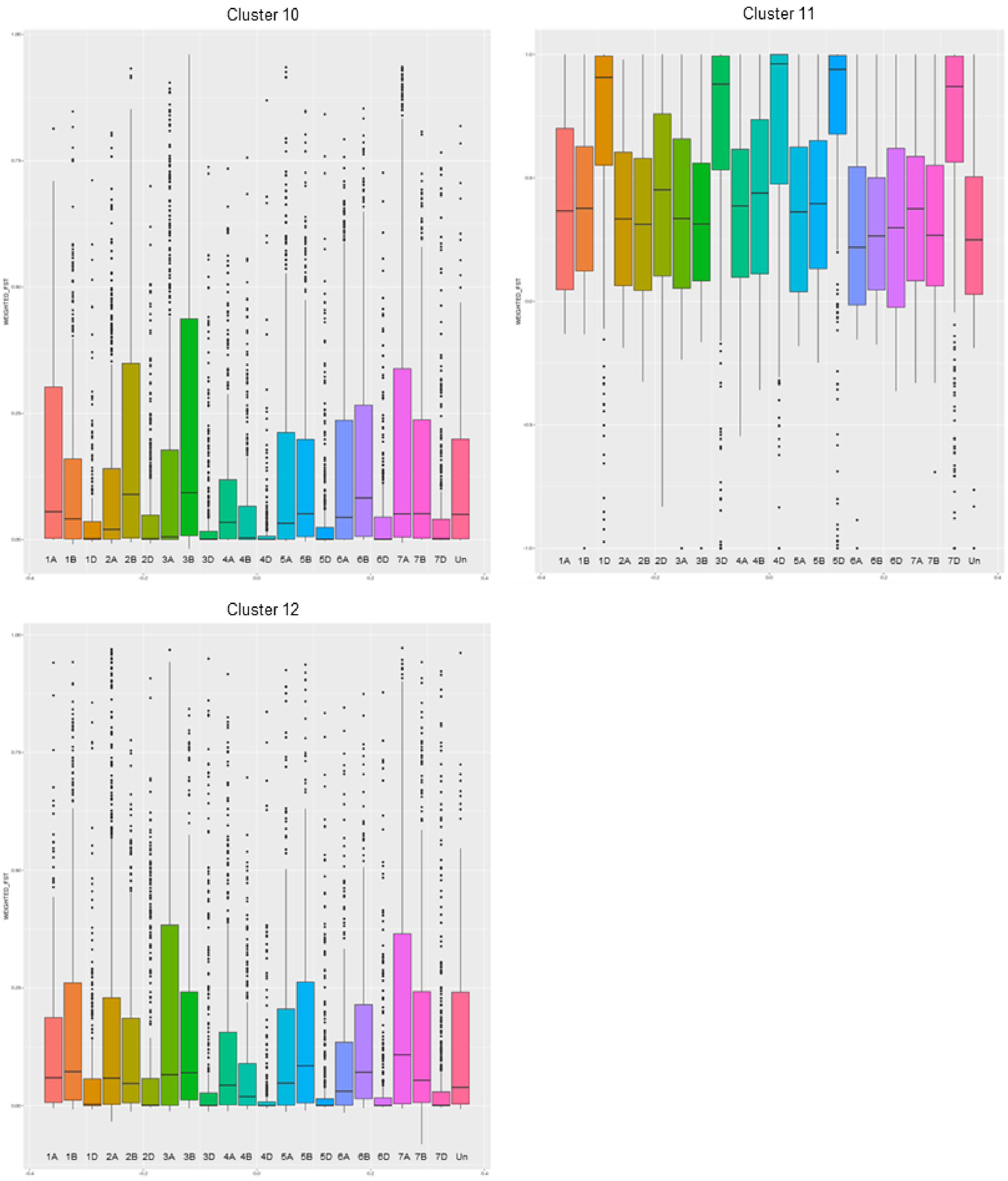

Supplementary Figure 15. Fst analysis per chromosome in hexaploid group.

Fst distributions across all chromosomes in the hexaploid group on cluster division 10, 11, 12.

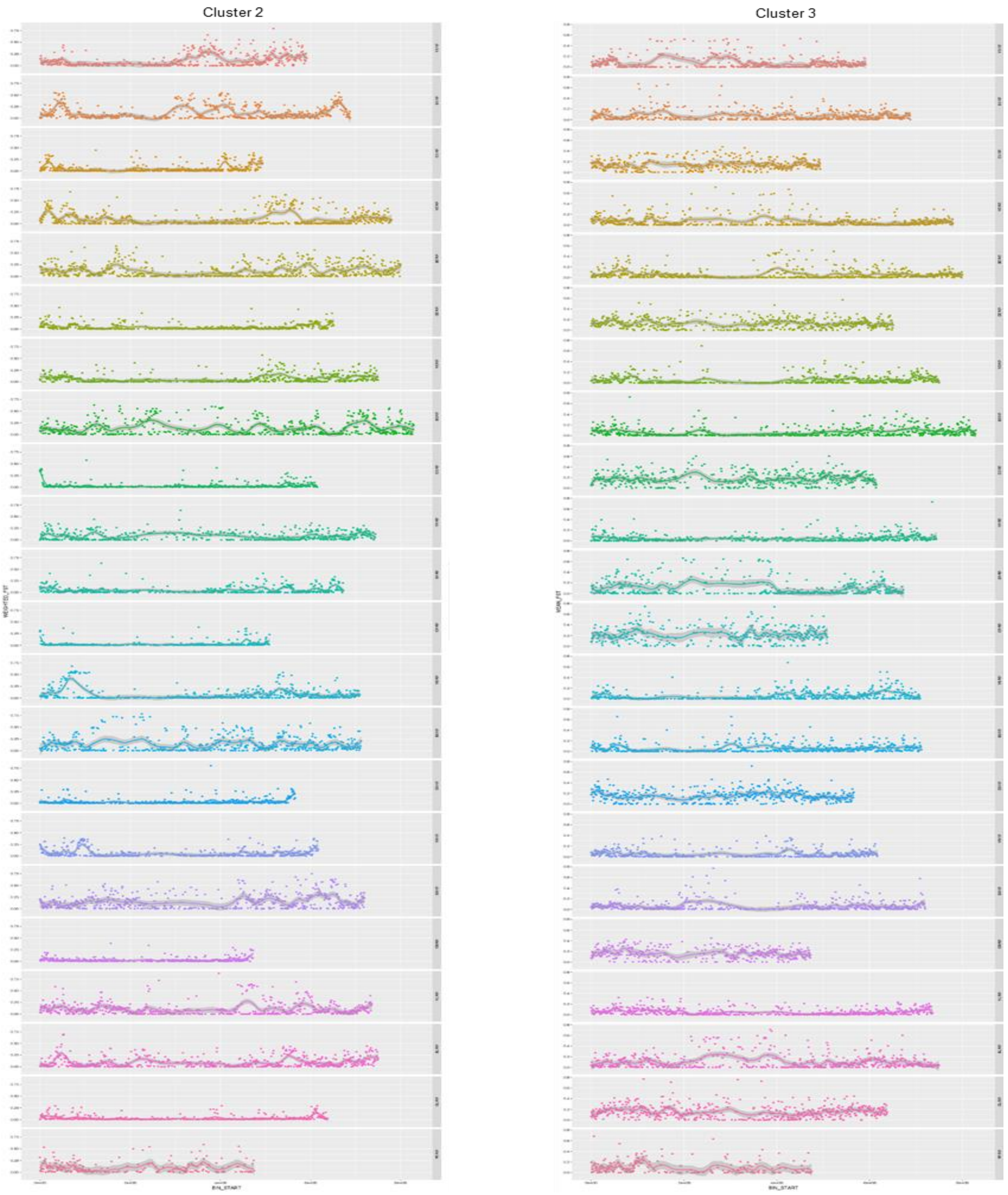

Supplementary Figure 16. Fst analysis inside chromosomes in hexaploid group.

Fst distributions across all chromosomes in the hexaploid group on cluster division 2 and 3.

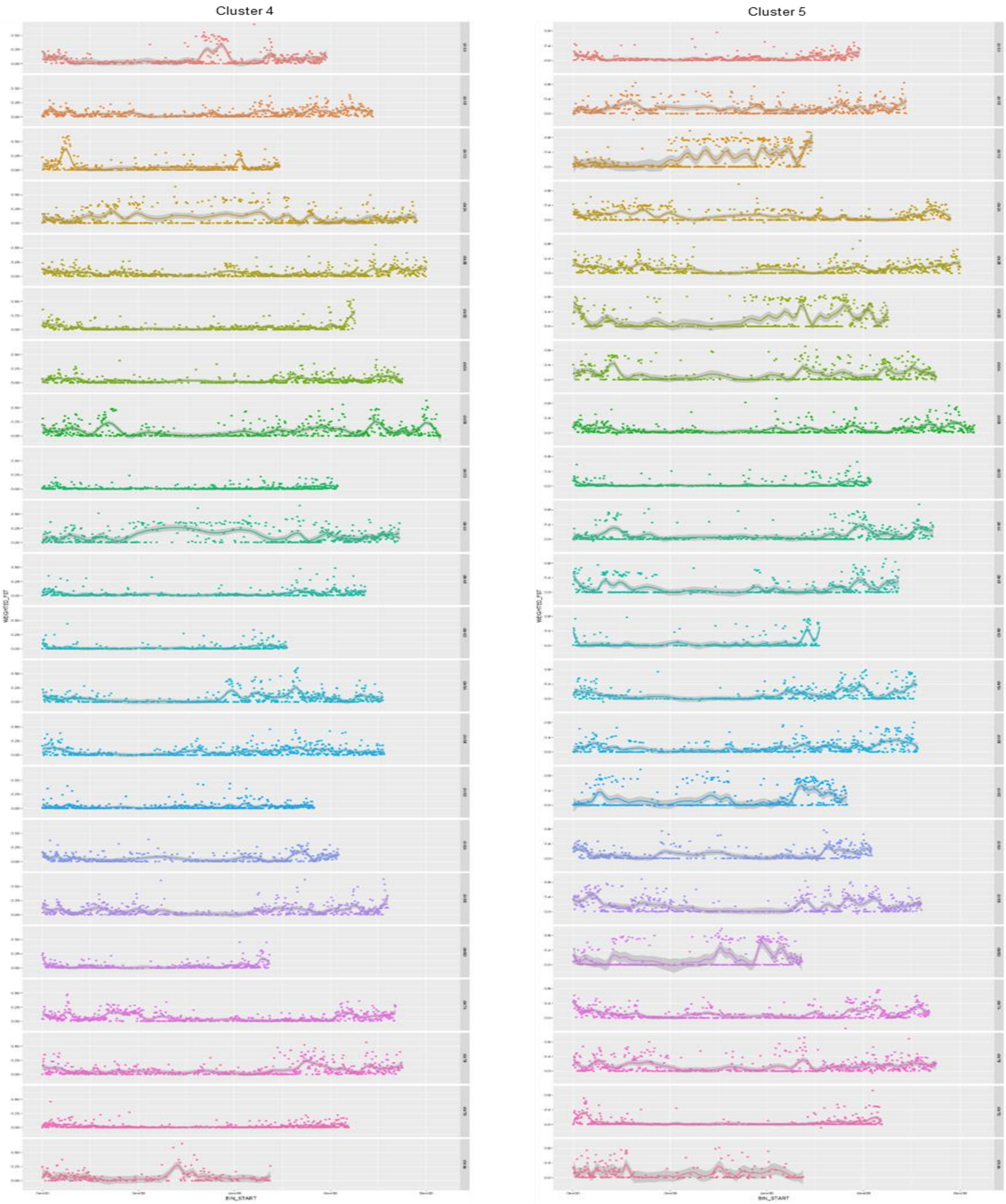

Supplementary Figure 17. Fst analysis inside chromosomes in hexaploid group.

Fst distributions across all chromosomes in the hexaploid group on cluster division 4 and 5.

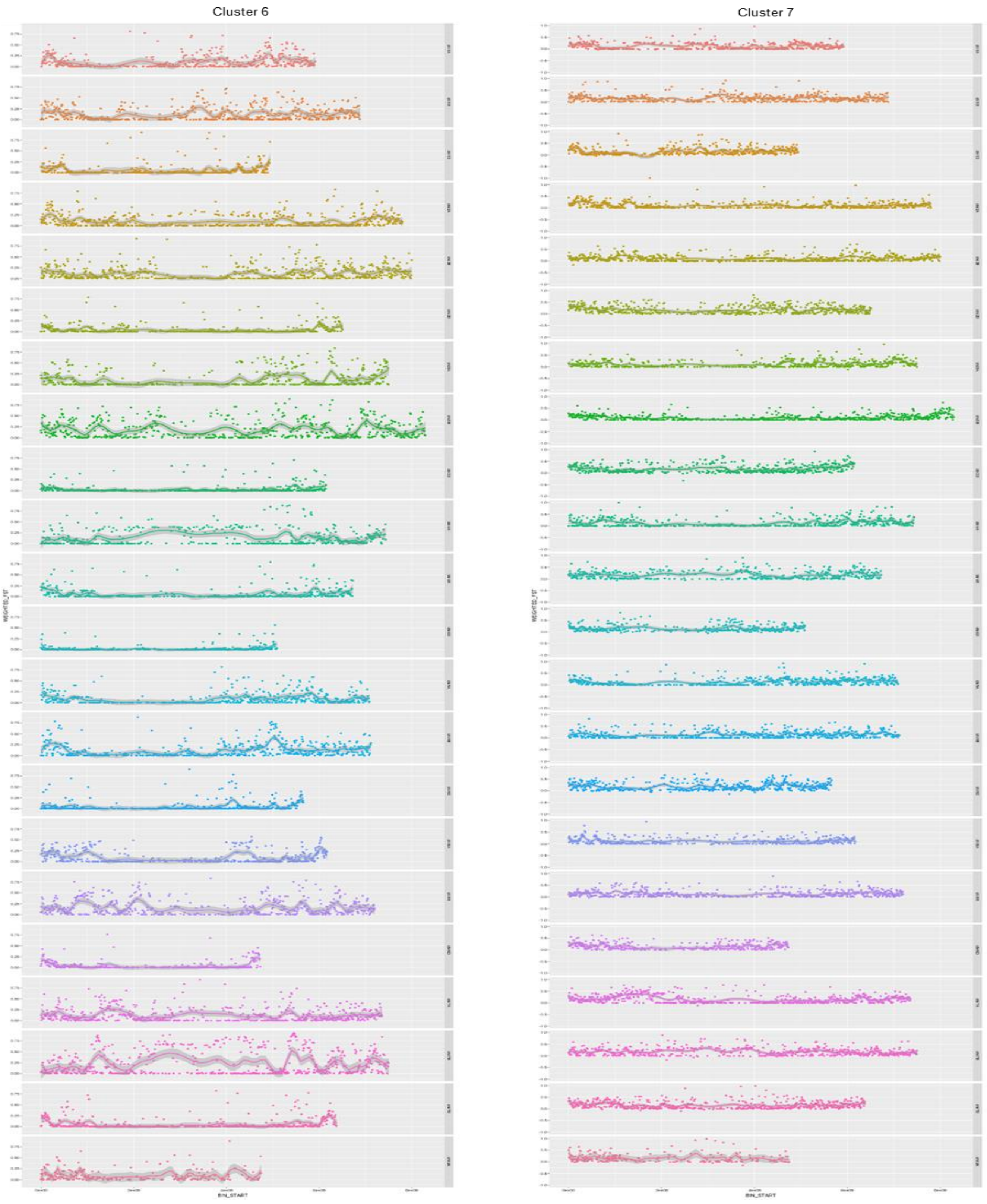

Supplementary Figure 18. Fst analysis inside chromosomes in hexaploid group.

Fst distributions across all chromosomes in the hexaploid group on cluster division 6 and 7.

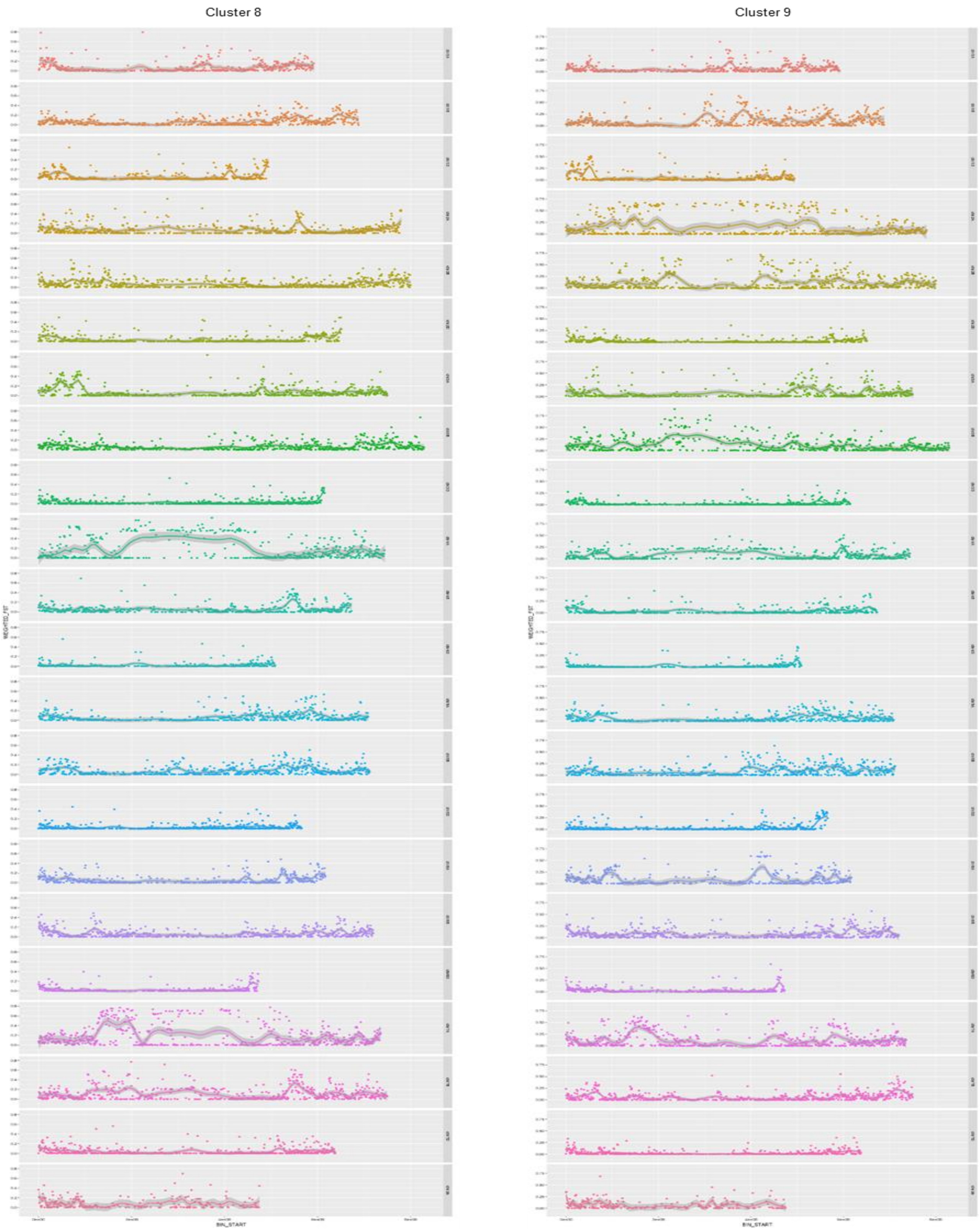

Supplementary Figure 19. Fst analysis inside chromosomes in hexaploid group.

Fst distributions across all chromosomes in the hexaploid group on cluster division 8 and 9.

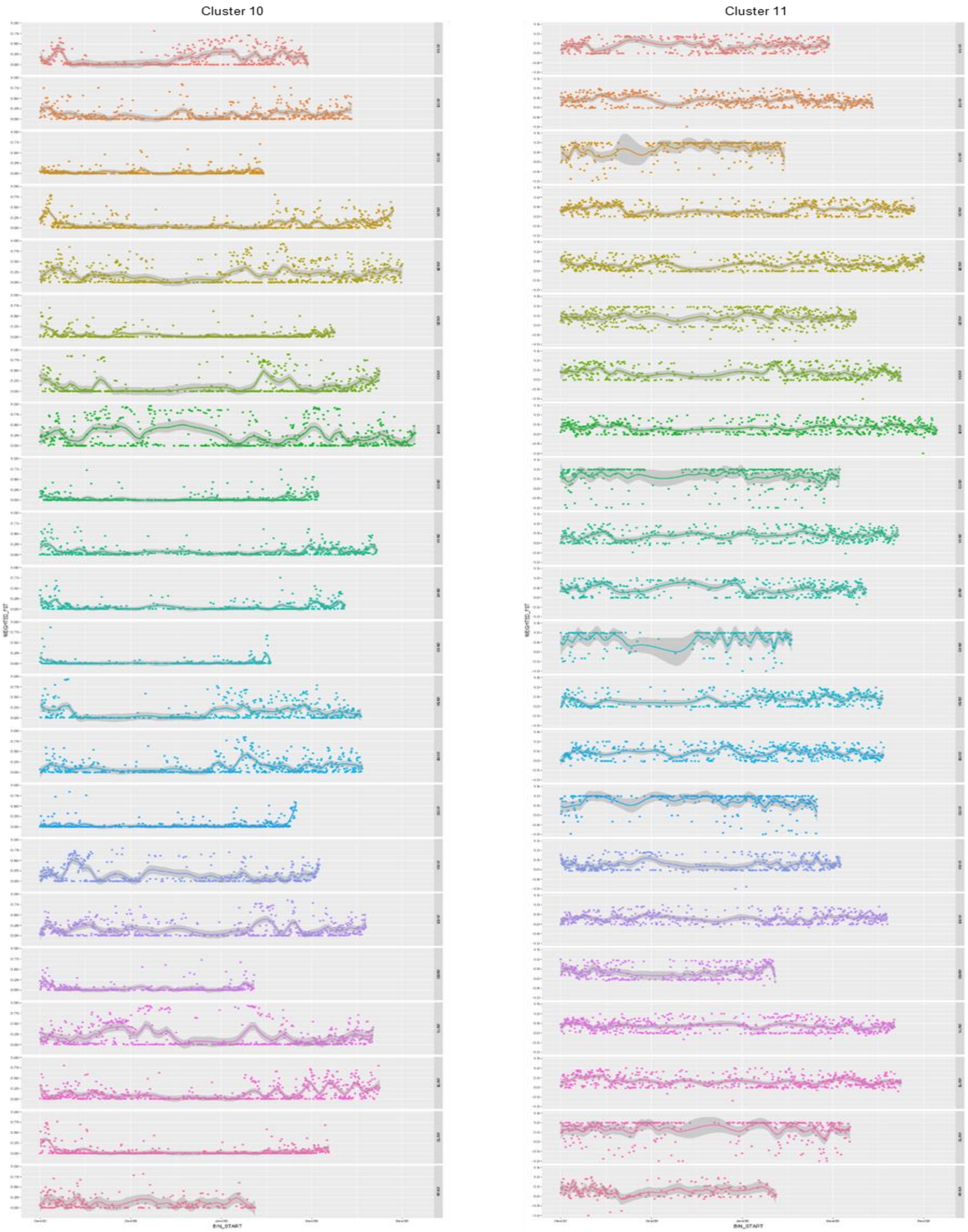

Supplementary Figure 20. Fst analysis inside chromosomes in hexaploid group.

Fst distributions across all chromosomes in the hexaploid group on cluster division 10 and 11.

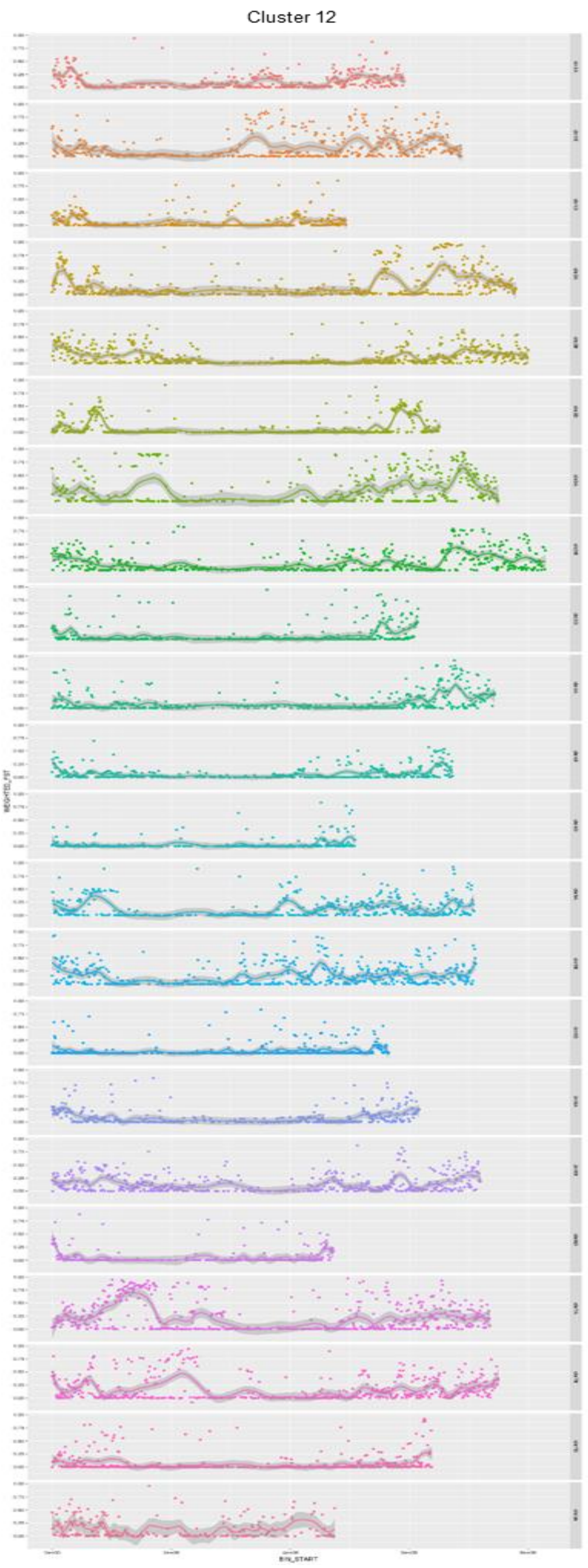

Supplementary Figure 21. Fst analysis inside chromosomes in hexaploid group.

Fst distributions across all chromosomes in the hexaploid group on cluster division 12.

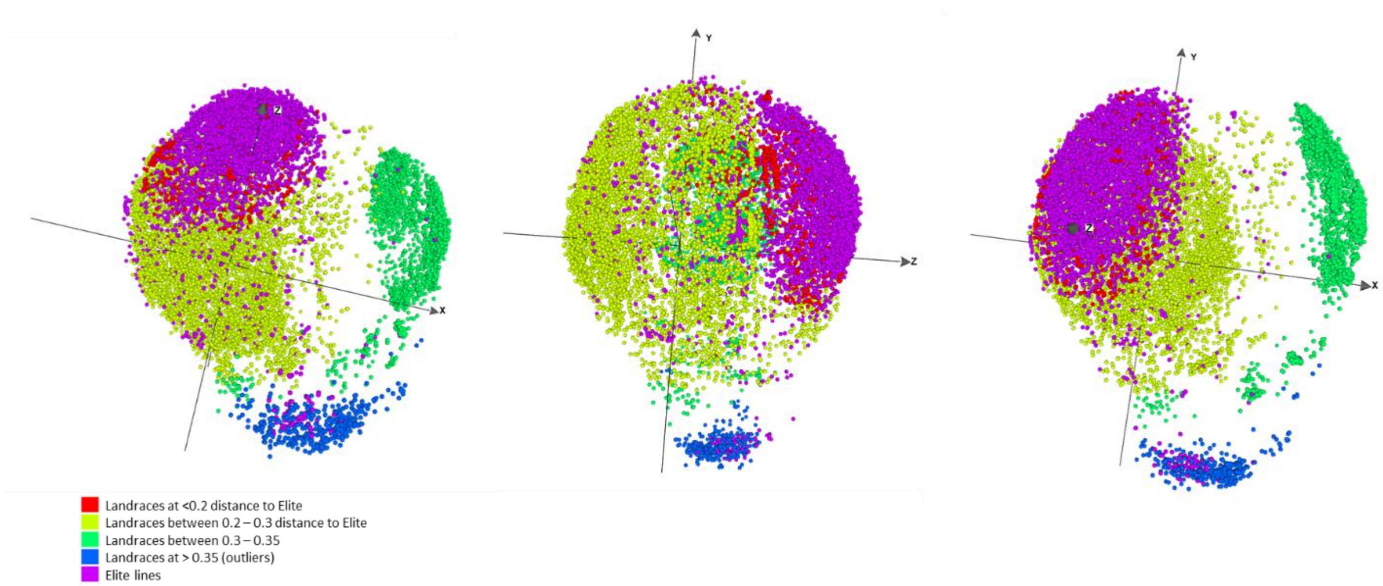

Supplementary Figure 22. MRD between landraces and elites/synthetics in tetraploid.

Different angles images of the MRD distance between each of the 10,801 landraces with the allelic frequencies of the group of 4,048 elite breeding lines shows four distinct groups, 837 landraces (7.8%) genetically very close to vast majority of the breeding elite lines with <0.2 distance, 6,029 landraces (55.8%) between 0.20 to 0.30 distance in which 29% of the accession are from Turkey and 10% from Iran, 3,554 (33%) between 0.30 and 0.35 distance in which 92% are form Ethiopia, and only 381 (3.5%) with more than 0.35 distance with the elite lines being 42% from Turkey.

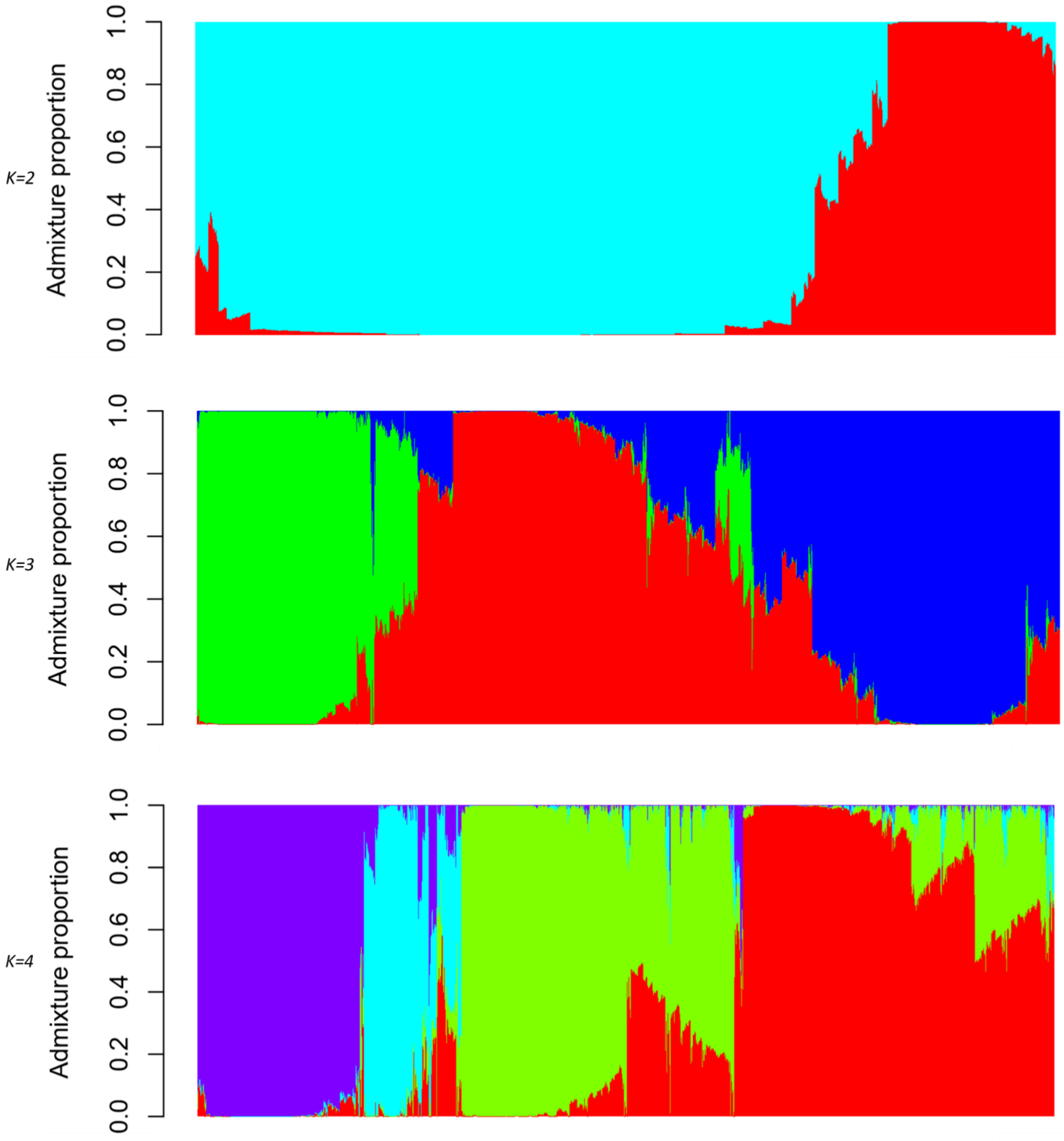

Supplementary Figure 23. ADMIXTURE analysis in tepraploid.

ADMIXTURE ancestry coefficients of a subset of tetraploid samples in K=2, K=3 and k=4.

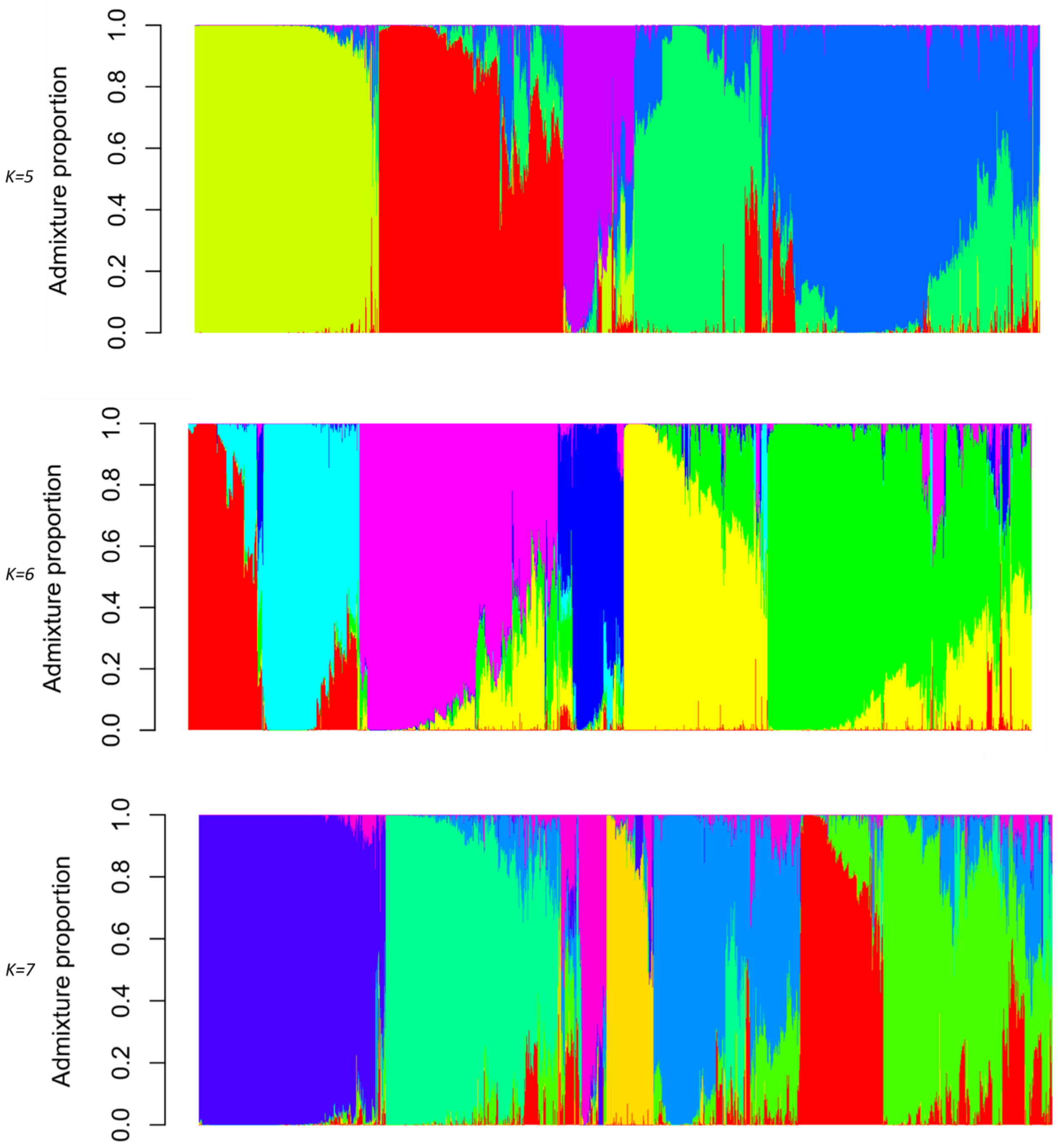

Supplementary Figure 24. ADMIXTURE analysis in tetraploid.

ADMIXTURE ancestry coefficients of a subset of tetraploid samples in K=5, K=6 and k=7.

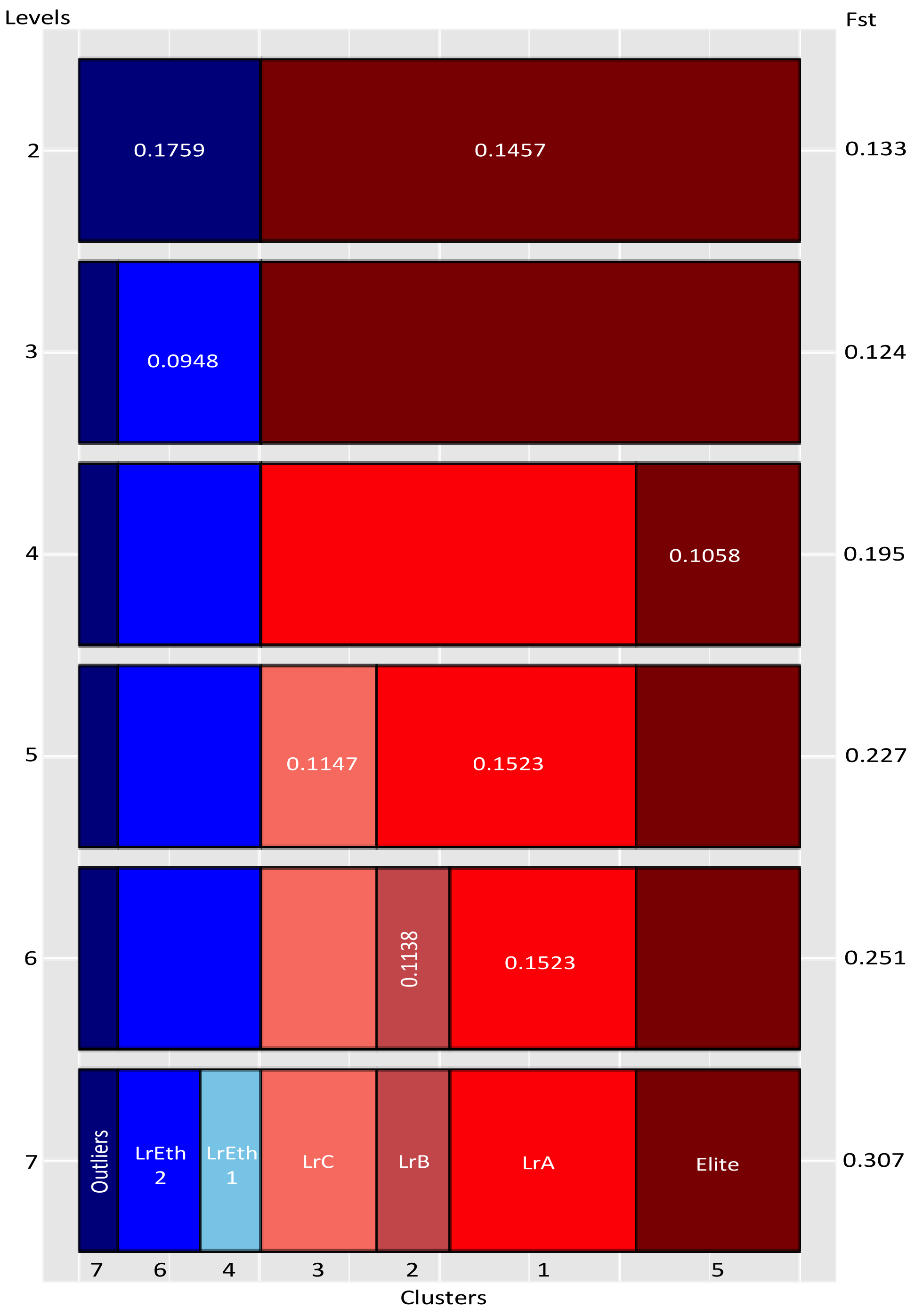

Supplementary Figure 25. Representation of the distribution of 7 groups based on clusters analysis. The size of the boxes is proportional to the number of accession. In the right side are the Fixation index (Fst) values and inside the boxes the expected heterocigocity (He) of each group and in the last boxes in group 7 are identified each group with a brief description and the number which correspond to Curlywhirly group.

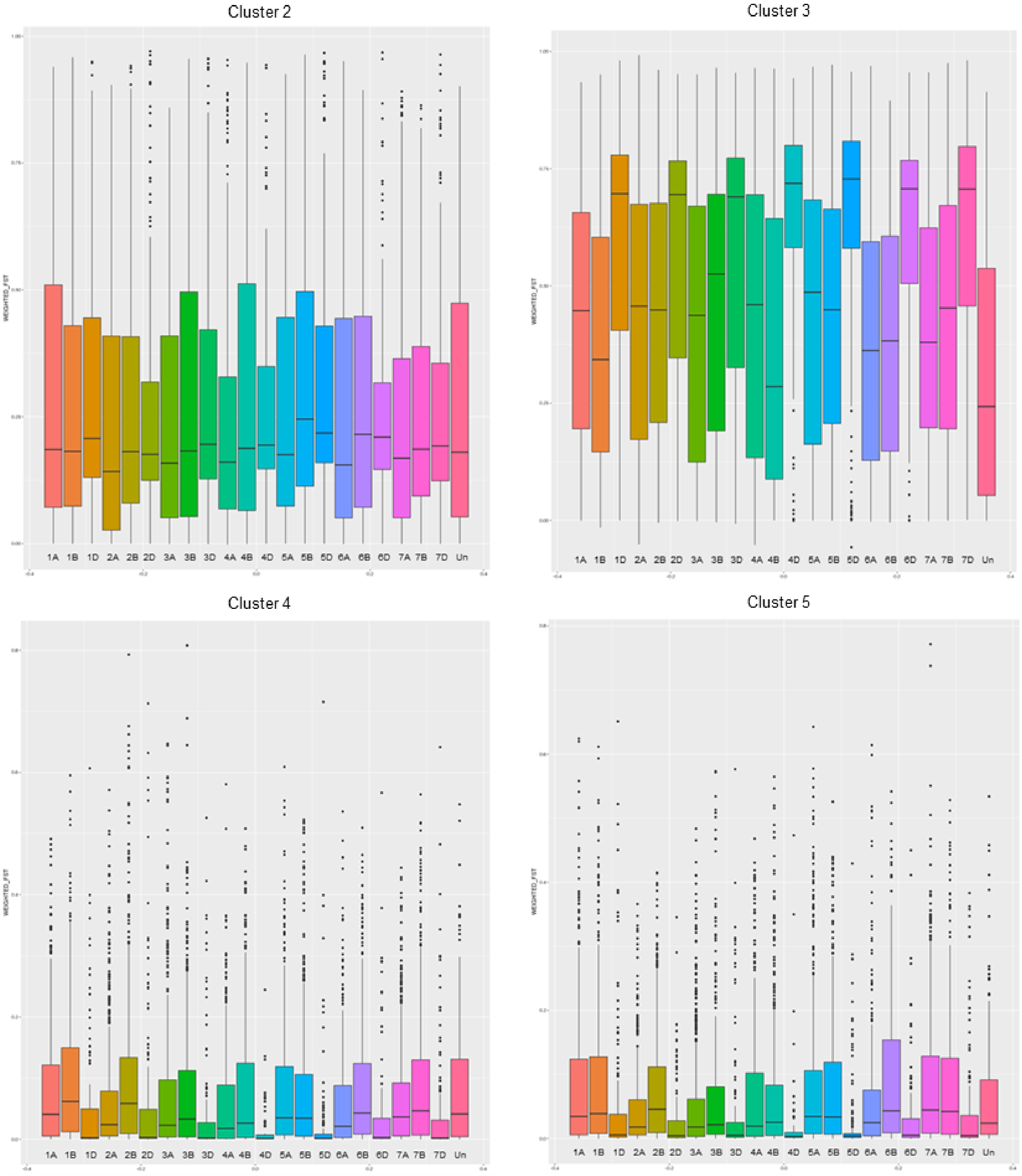

Supplementary Figure 26. Fst analysis per chromosome in tetraploid group.

Fst distributions across all chromosomes in the tetraploid group on cluster division 2, 3, 4 and 5.

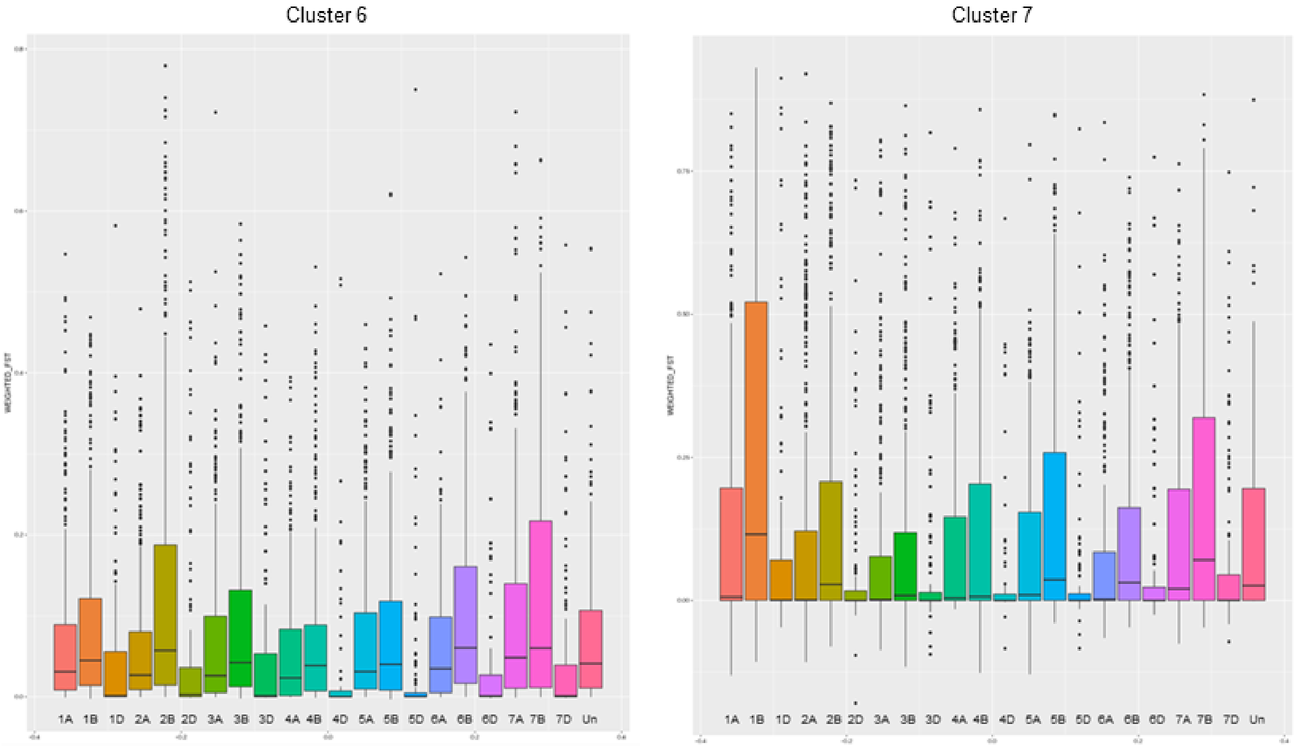

Supplementary Figure 27. Fst analysis per chromosome in tetraploid group.

Fst distributions across all chromosomes in the tetraploid group on cluster division 6 and 7.

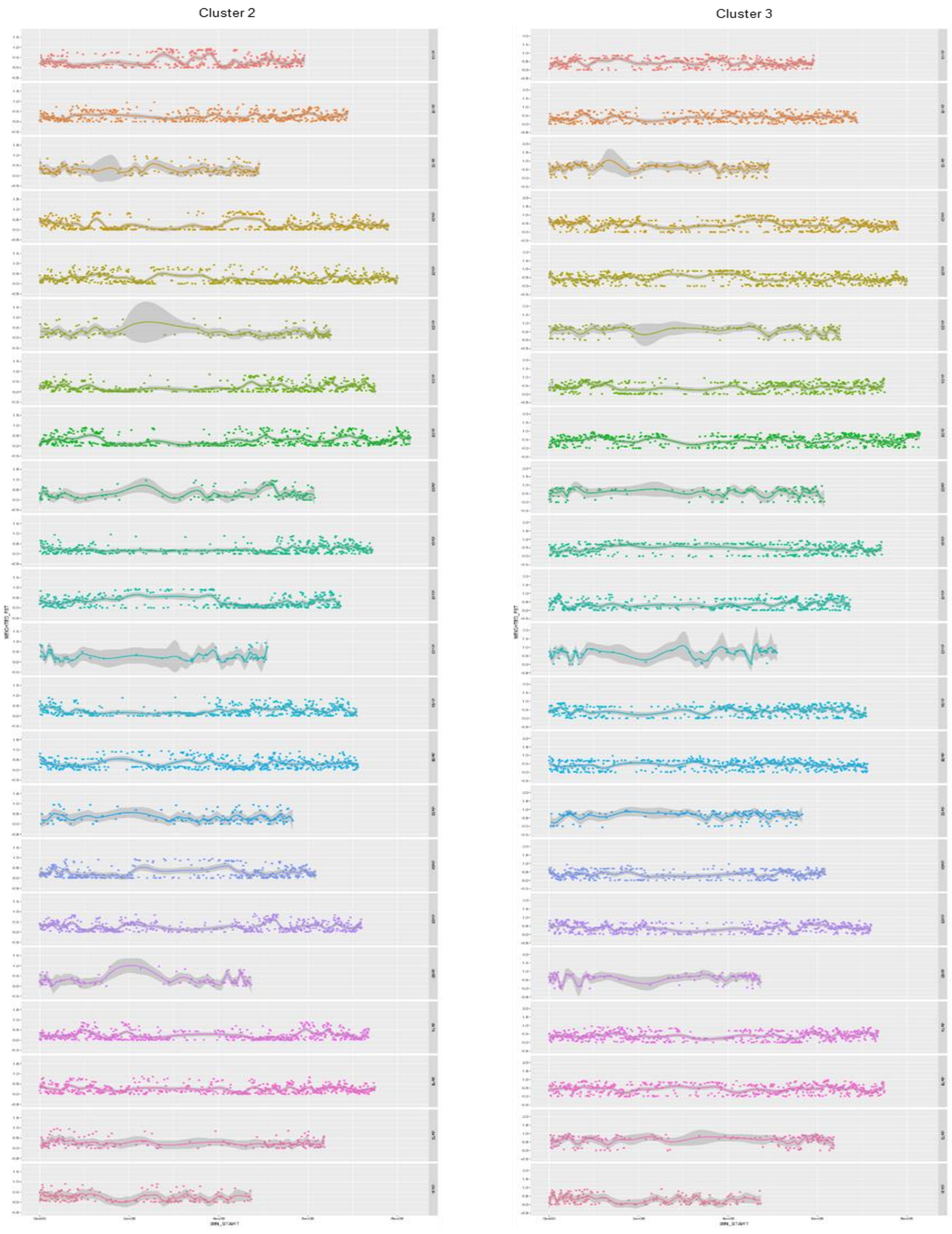

Supplementary Figure 28. Fst analysis inside chromosomes in tetraploid group.

Fst distributions across all chromosomes in the tetraploid group on cluster division 2 and 3.

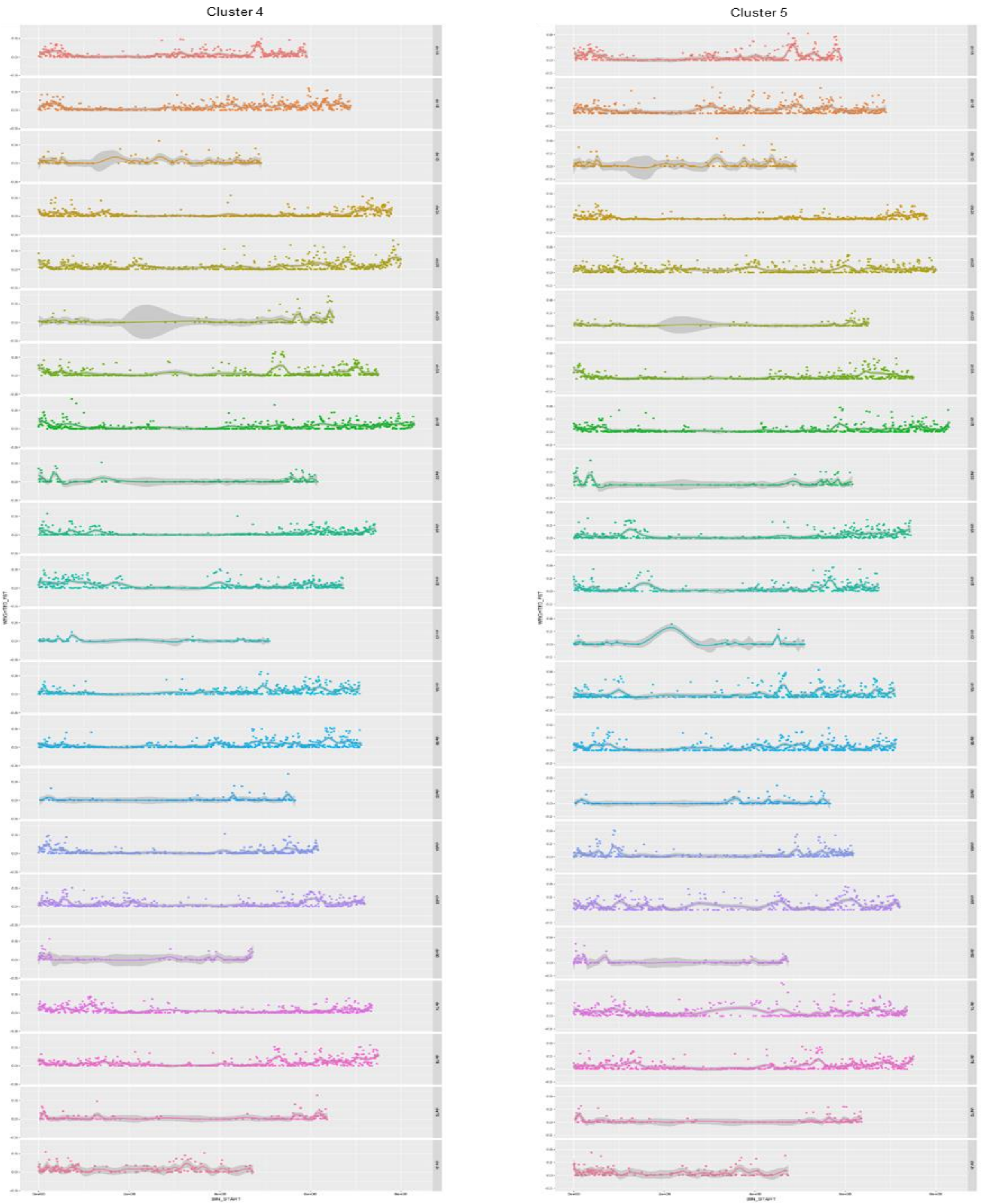

Supplementary Figure 29. Fst analysis inside chromosomes in tetraploid group.

Fst distributions across all chromosomes in the tetraploid group on cluster division 4 and 5.

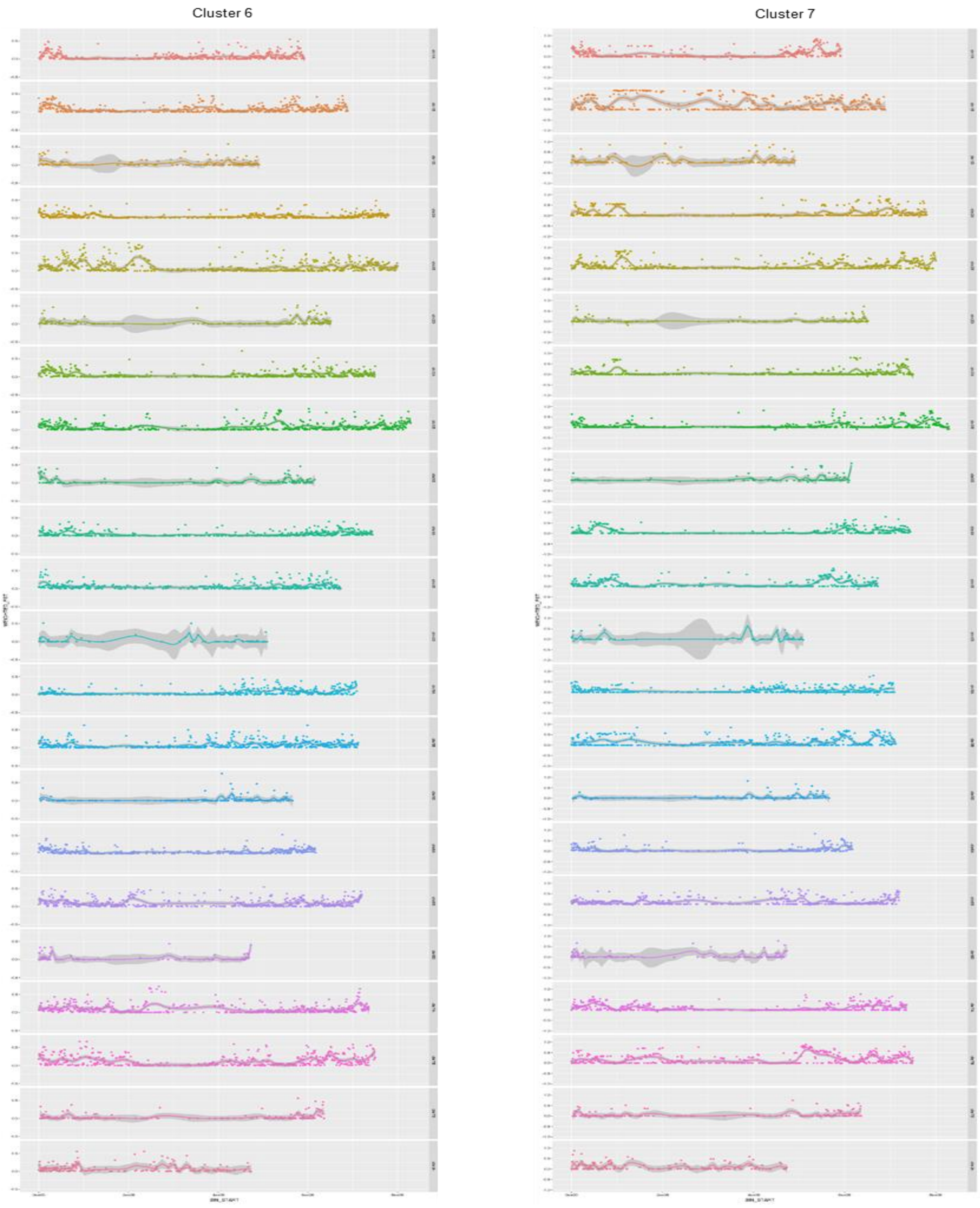

Supplementary Figure 30. Fst analysis inside chromosomes in tetraploid group.

Fst distributions across all chromosomes in the tetraploid group on cluster division 6 and 7.

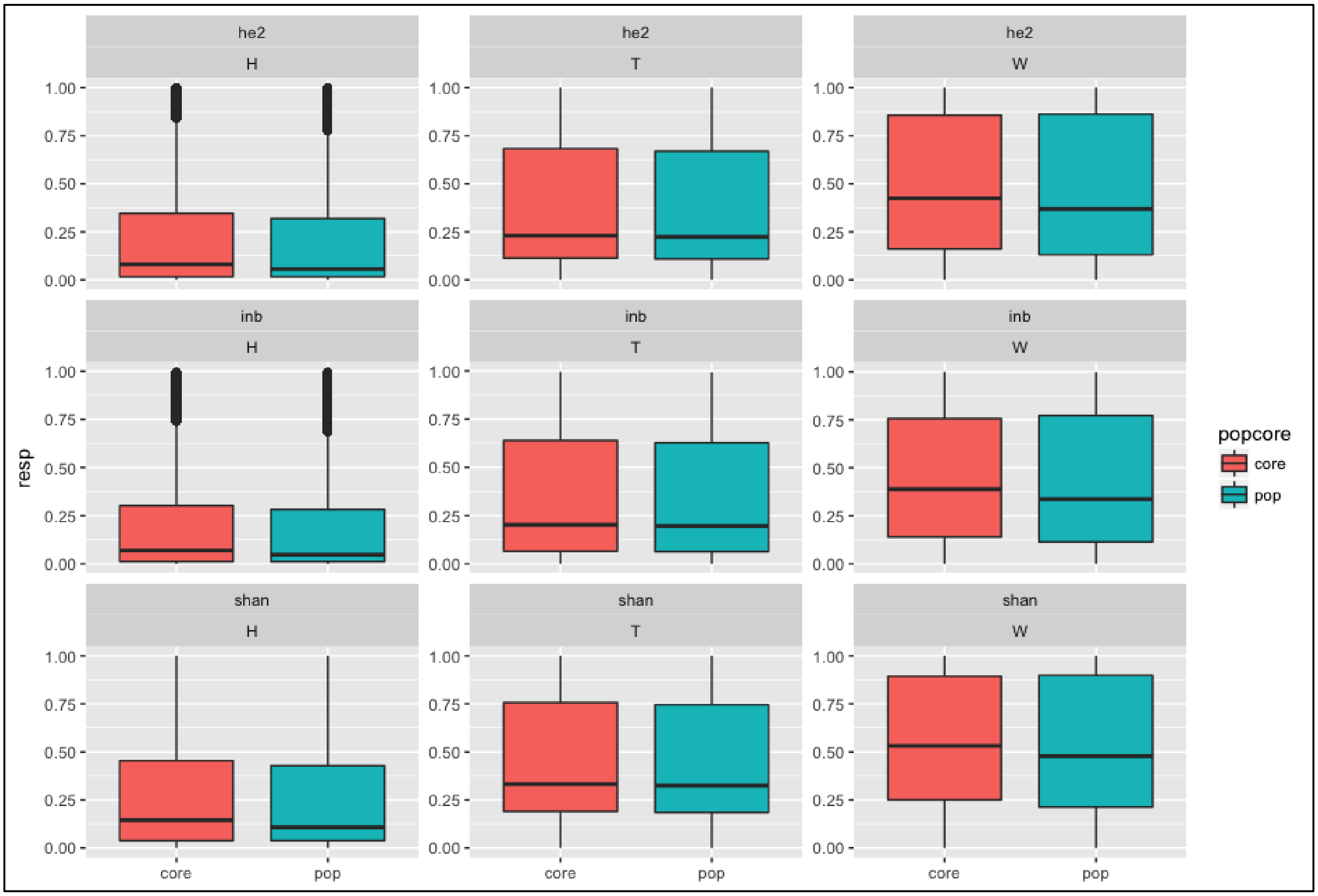

Supplementary Figure 31. Boxplots of diversity indexes.

Expected heterocigocity (He2), Inbreeding (inb) and Shanon index (shan) in the core subset (red) and in the complete population (blue) across the three biological groups, hexaploid (H), tetraploid (T) and CWR (W). Upper box = percentile 75, lower box= perceptible 25, line in the box = Median, lower line (whisker) = 1.5*(percentile 25), upper whisker = 1.5*(percentile 75), dots at top: outliers = values greater than the 1.5*(P75 whisker).

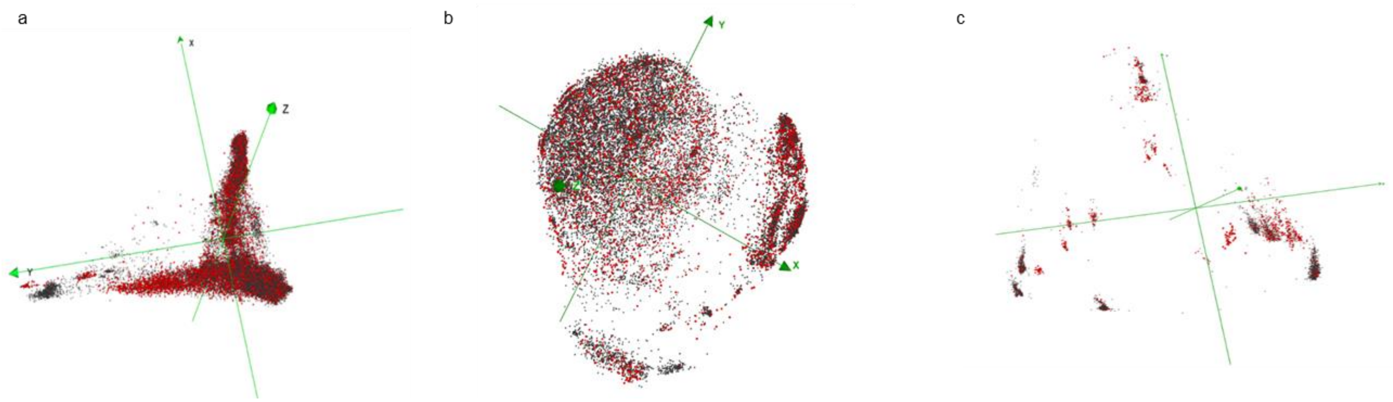

Supplementary Figure 32. Core subset of hexaploid, tetraploid and CWR.

Core sub set representing 20% of the population of hexaploid, tetraploids and CWR accessions. The same data points as in Figure 1 are shown. Samples are colored red if they are part of the core set, all other samples are shown in gray. a) core subset of 11,235 domesticated hexaploid accessions, b) core reference set of 3,762 domesticated tetraploid accessions, c) core reference set of 748 CWR accessions.

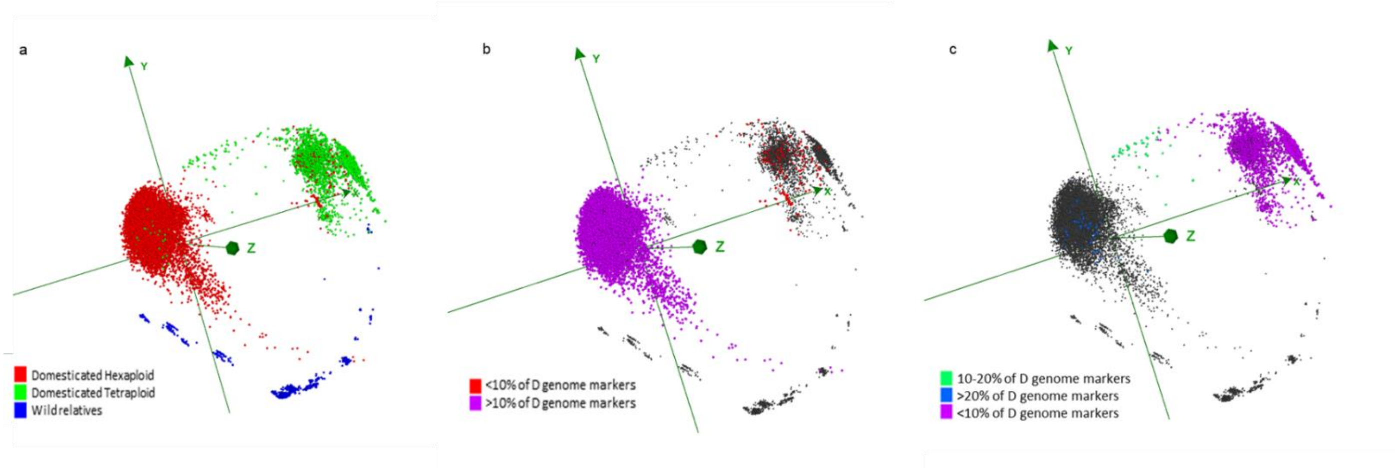

Supplementary Figure 33. Representation of the global diversity and outliers.

Multidimensional scaling representation of the global diversity analysis combining the three core subset using 41,717 SilicoDArT markers. a) 746 samples of Wilds in blue, 3,157 samples of tepraploids in green and 11,235 samples of hexaploids in red; b) MDS plot showing the hexaploids samples in purpure and 2.12% of the samples in red with potential miss-classification in their passport data which contain <10% of D genome markers (outliers); c) tepraploid samples in purpure and 4.37% of potential miss-classification in their passport information with 10 to 20% (green) and >20% (blue) of D genome markers (outliers).

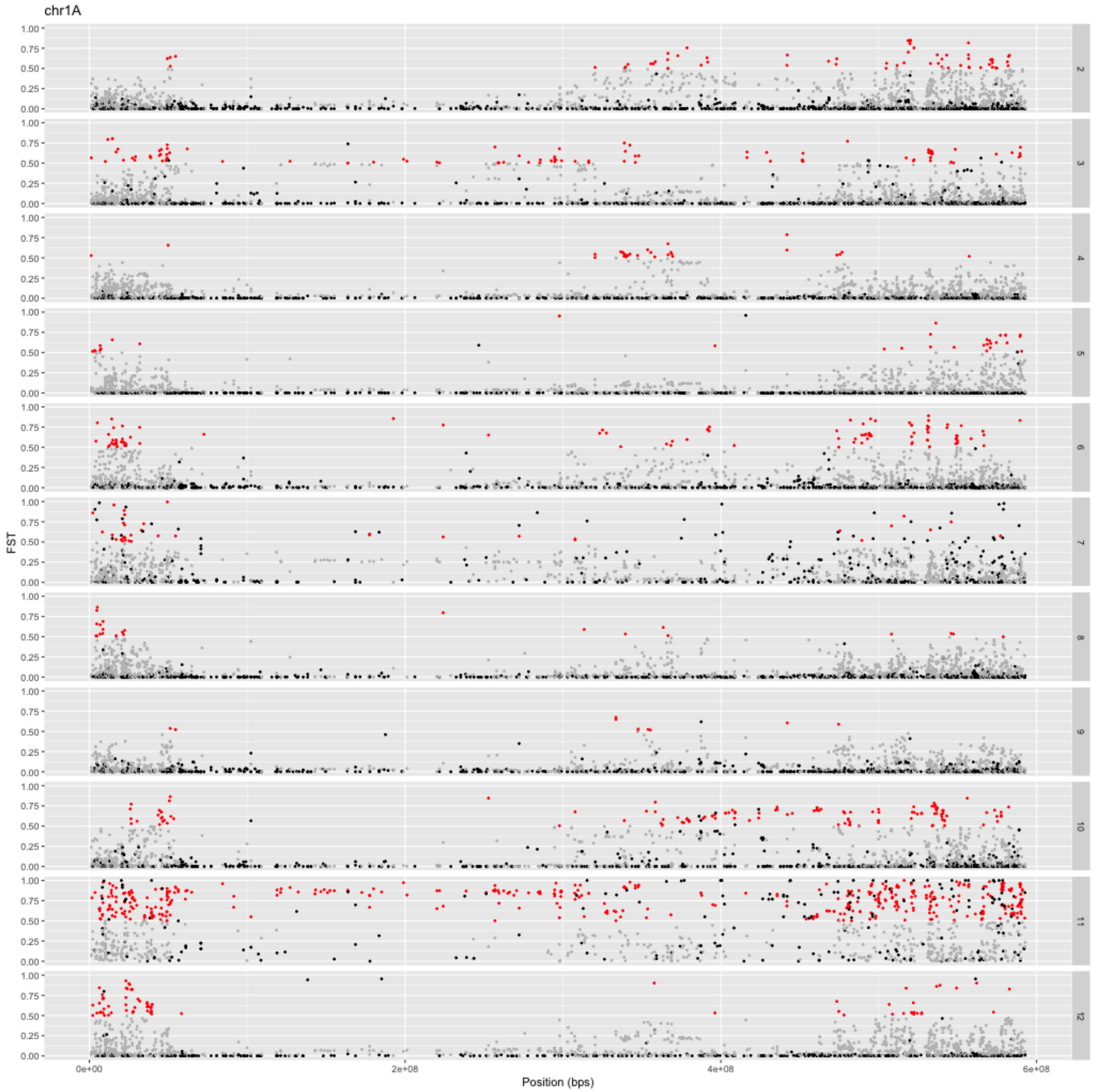

Supplementary Figure 34. Diversity patterns per cluster division in hexaploid.

Analysis of Fst values on a variant per variant basis on chromosome 1A across the 12 clusters divisions.

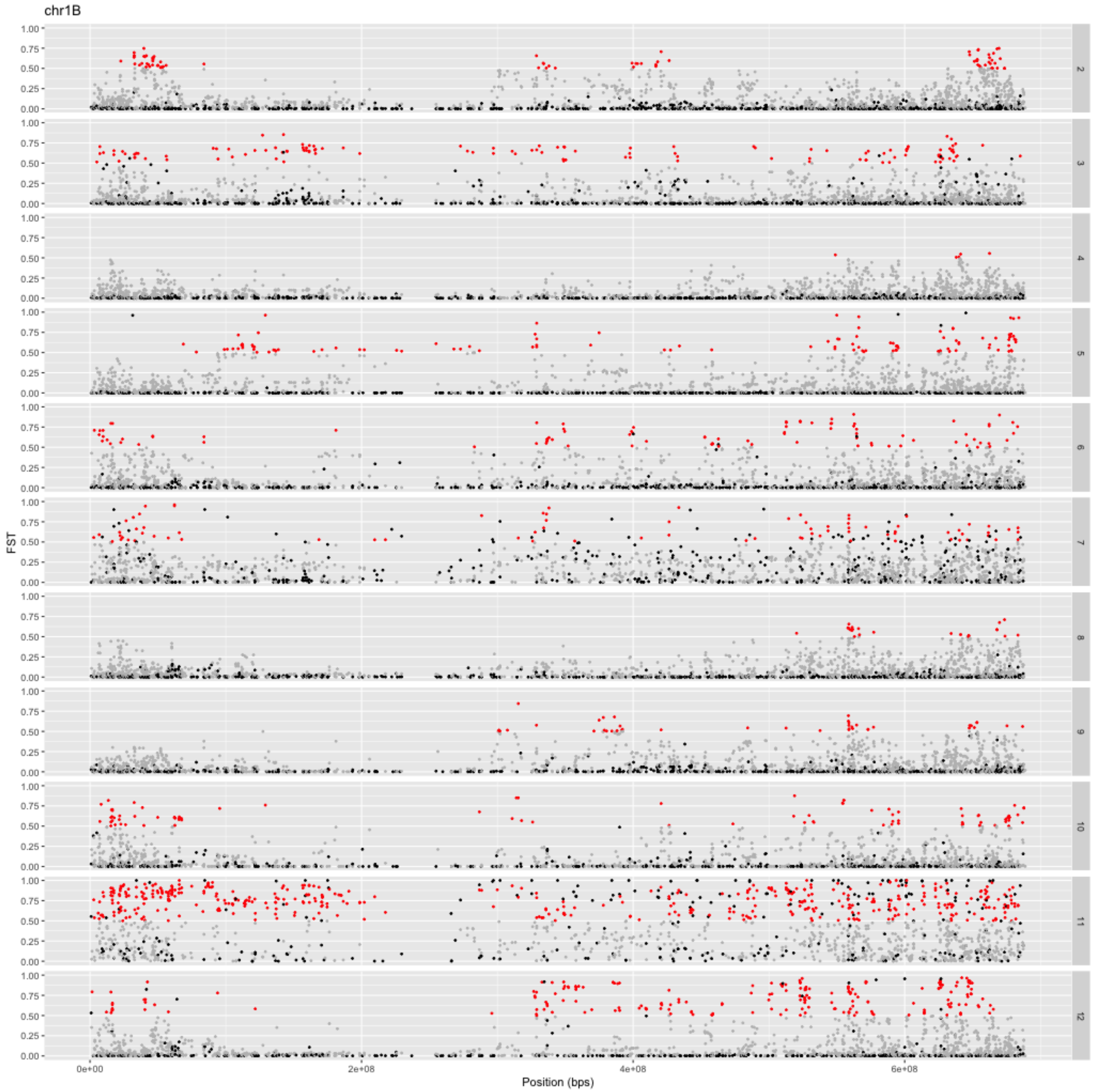

Supplementary Figure 35. Diversity patterns per cluster division in hexaploid.

Analysis of Fst values on a variant per variant basis on chromosome 1B across the 12 clusters divisions.

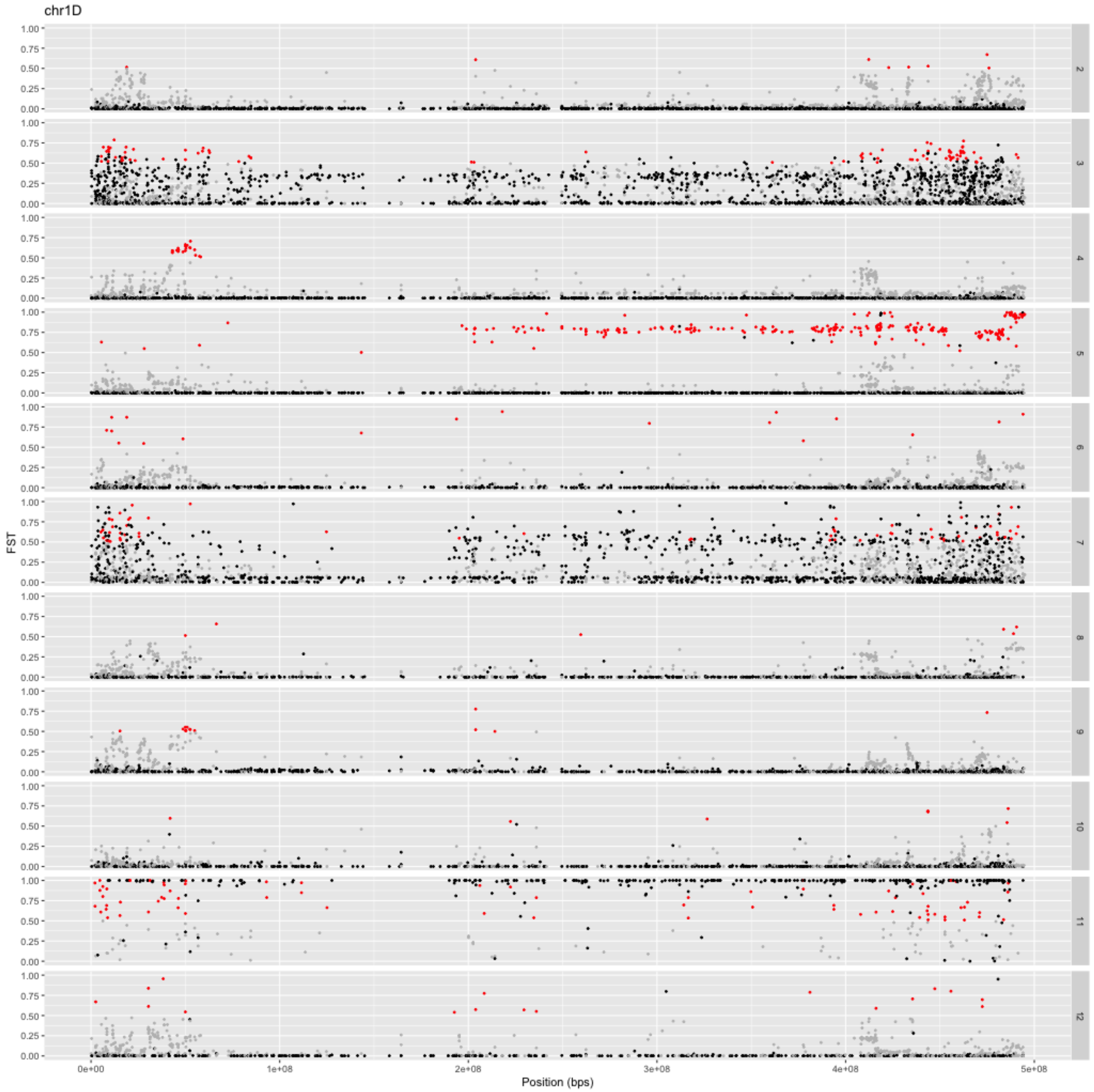

Supplementary Figure 36. Diversity patterns per cluster division in hexaploid.

Analysis of Fst values on a variant per variant basis on chromosome 1D across the 12 clusters divisions.

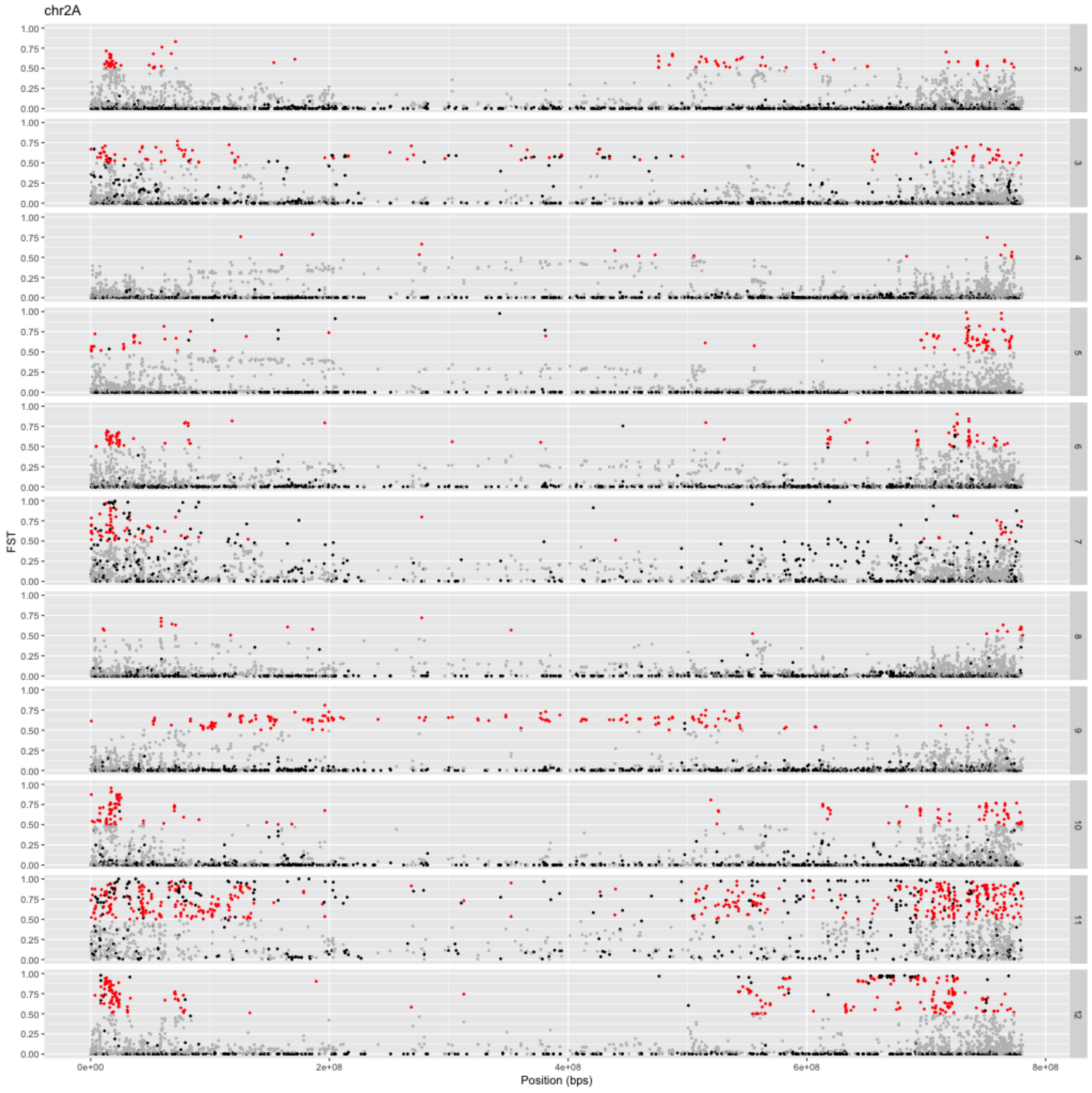

Supplementary Figure 37. Diversity patterns per cluster division in hexaploid.

Analysis of Fst values on a variant per variant basis on chromosome 2A across the 12 clusters divisions.

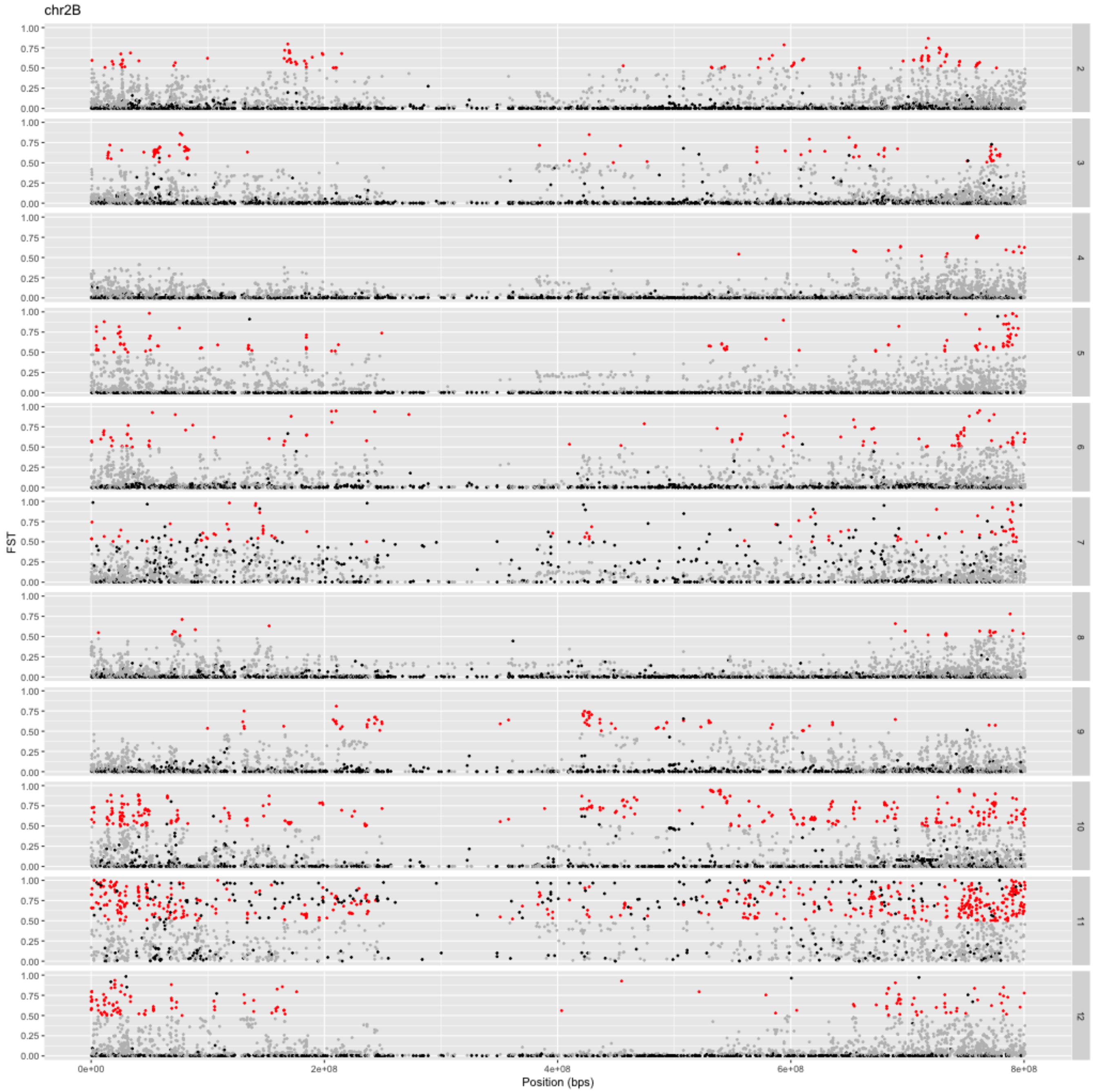

Supplementary Figure 38. Diversity patterns per cluster division in hexaploid.

Analysis of Fst values on a variant per variant basis on chromosome 2B across the 12 clusters divisions.

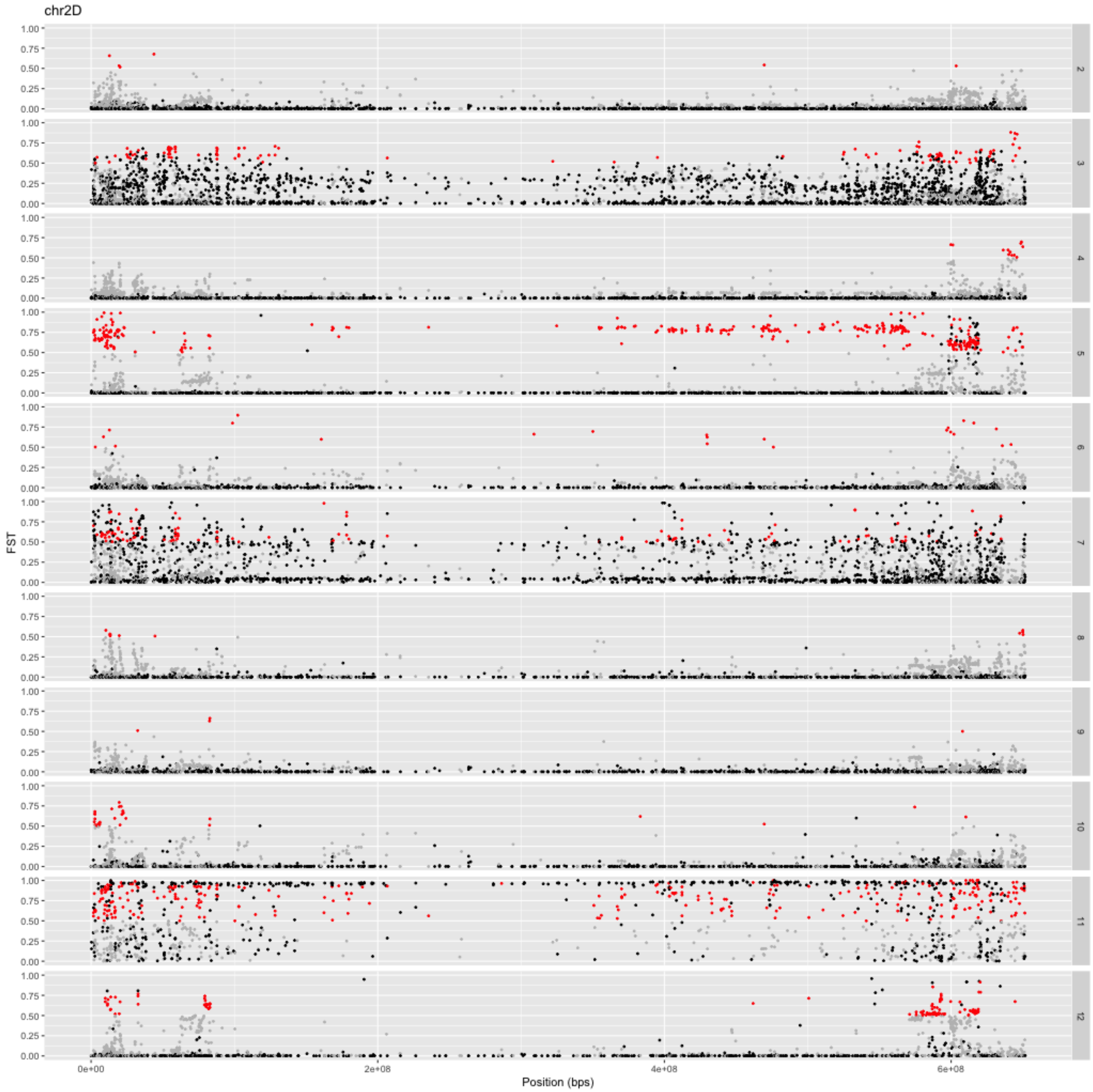

Supplementary Figure 39. Diversity patterns per cluster division in hexaploid.

Analysis of Fst values on a variant per variant basis on chromosome 2D across the 12 clusters divisions.

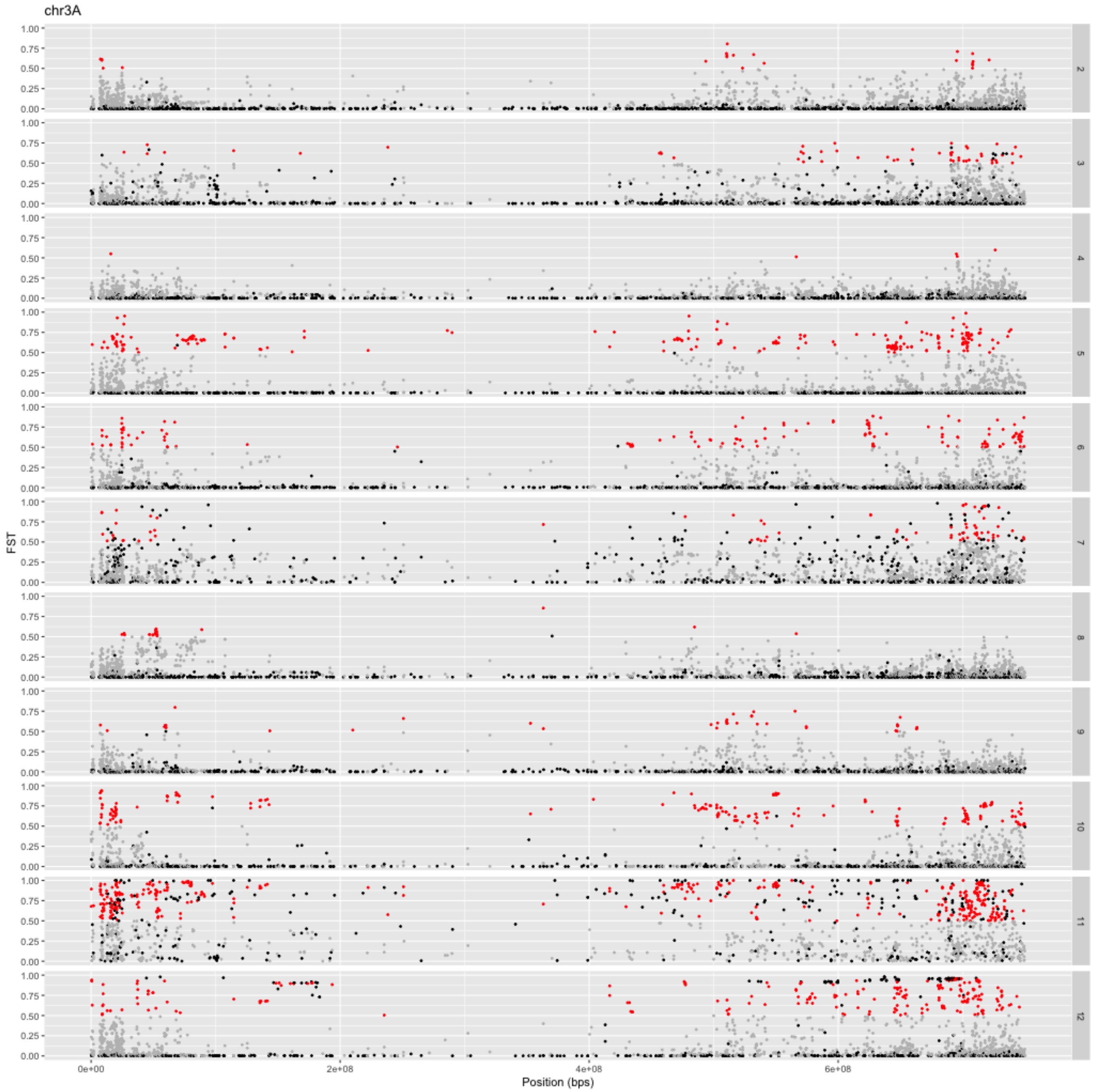

Supplementary Figure 40. Diversity patterns per cluster division in hexaploid.

Analysis of Fst values on a variant per variant basis on chromosome 3A across the 12 clusters divisions.

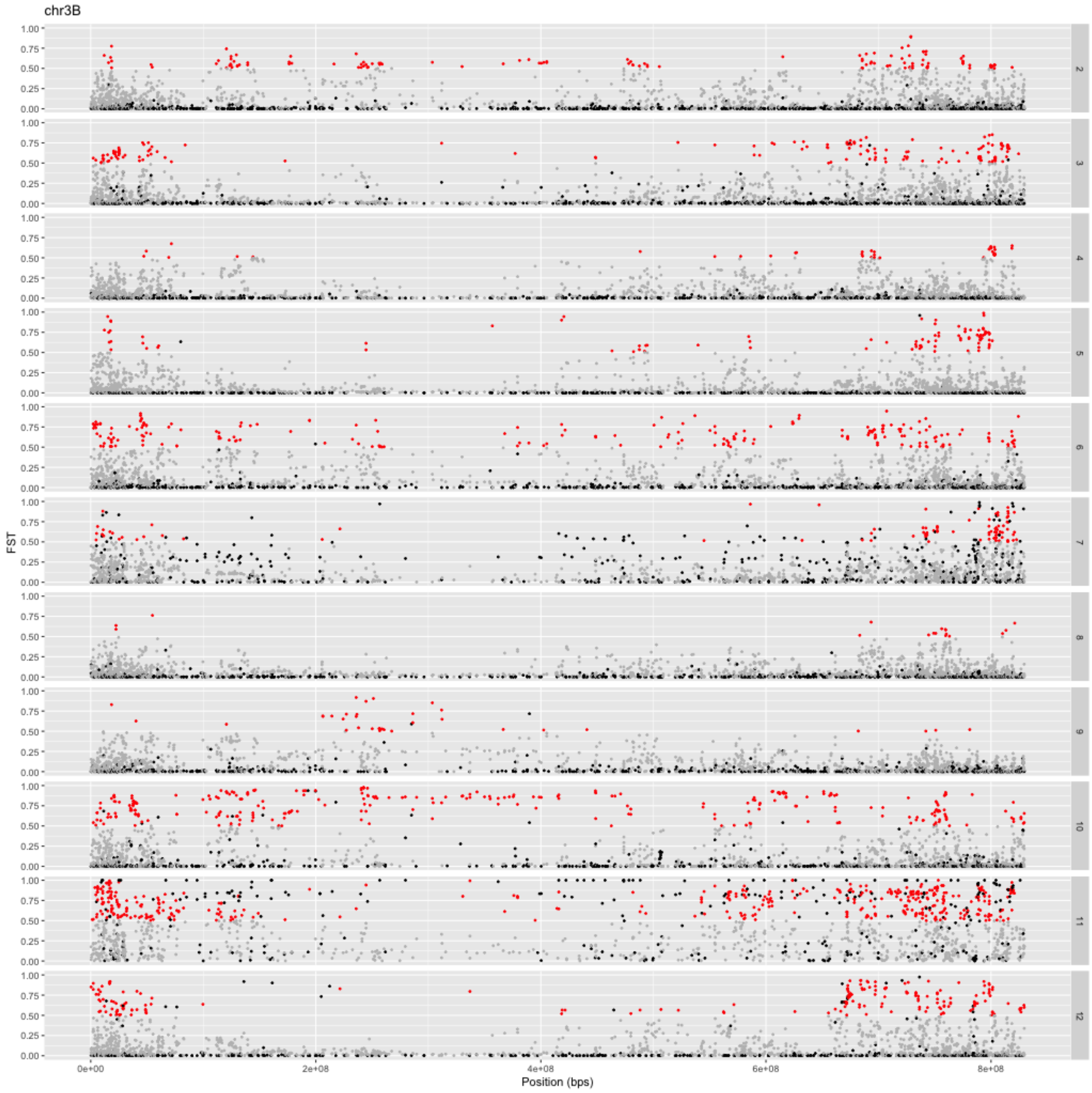

Supplementary Figure 41. Diversity patterns per cluster division in hexaploid.

Analysis of Fst values on a variant per variant basis on chromosome 3B across the 12 clusters divisions.

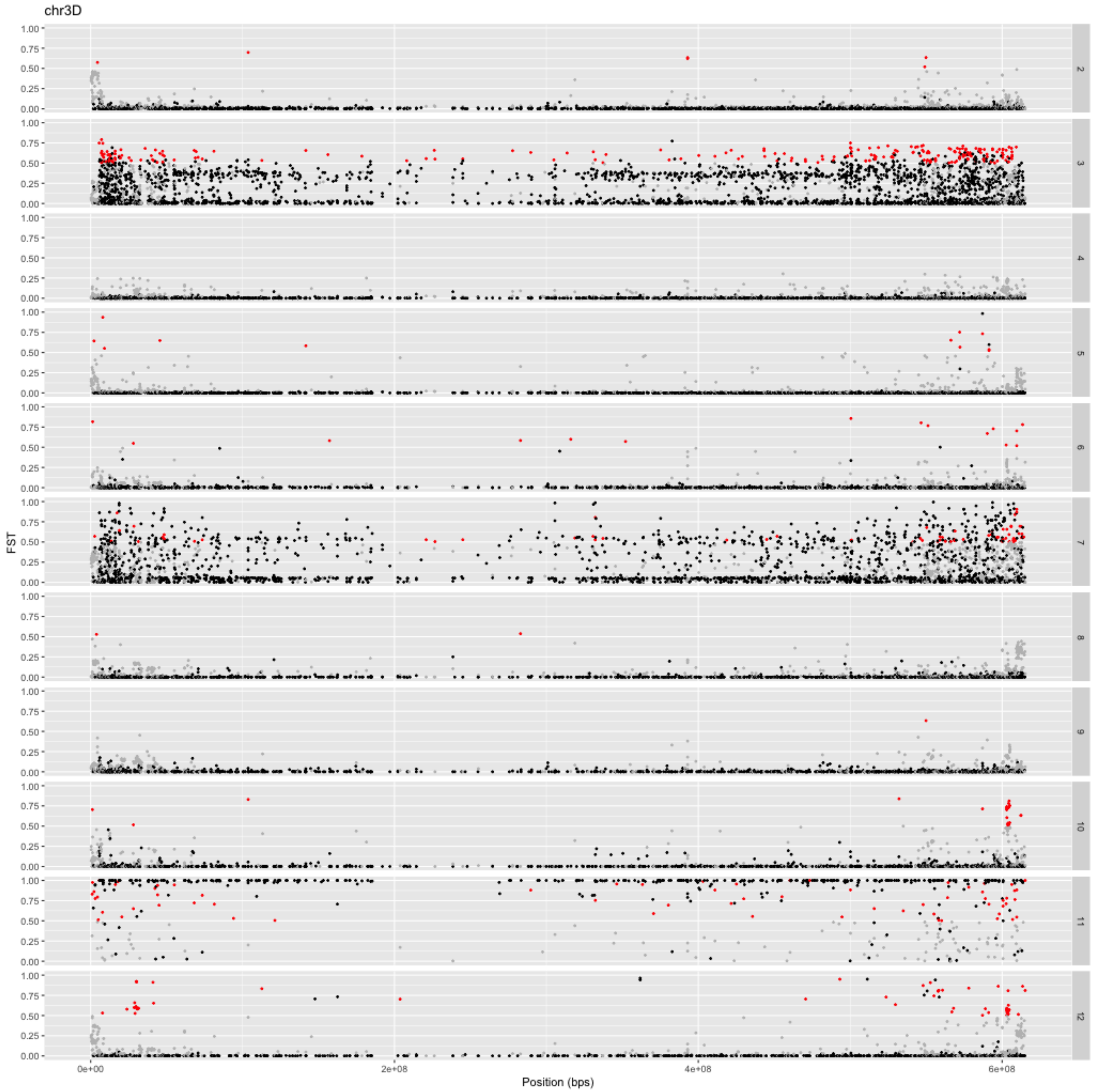

Supplementary Figure 42. Diversity patterns per cluster division in hexaploid.

Analysis of Fst values on a variant per variant basis on chromosome 3D across the 12 clusters divisions.

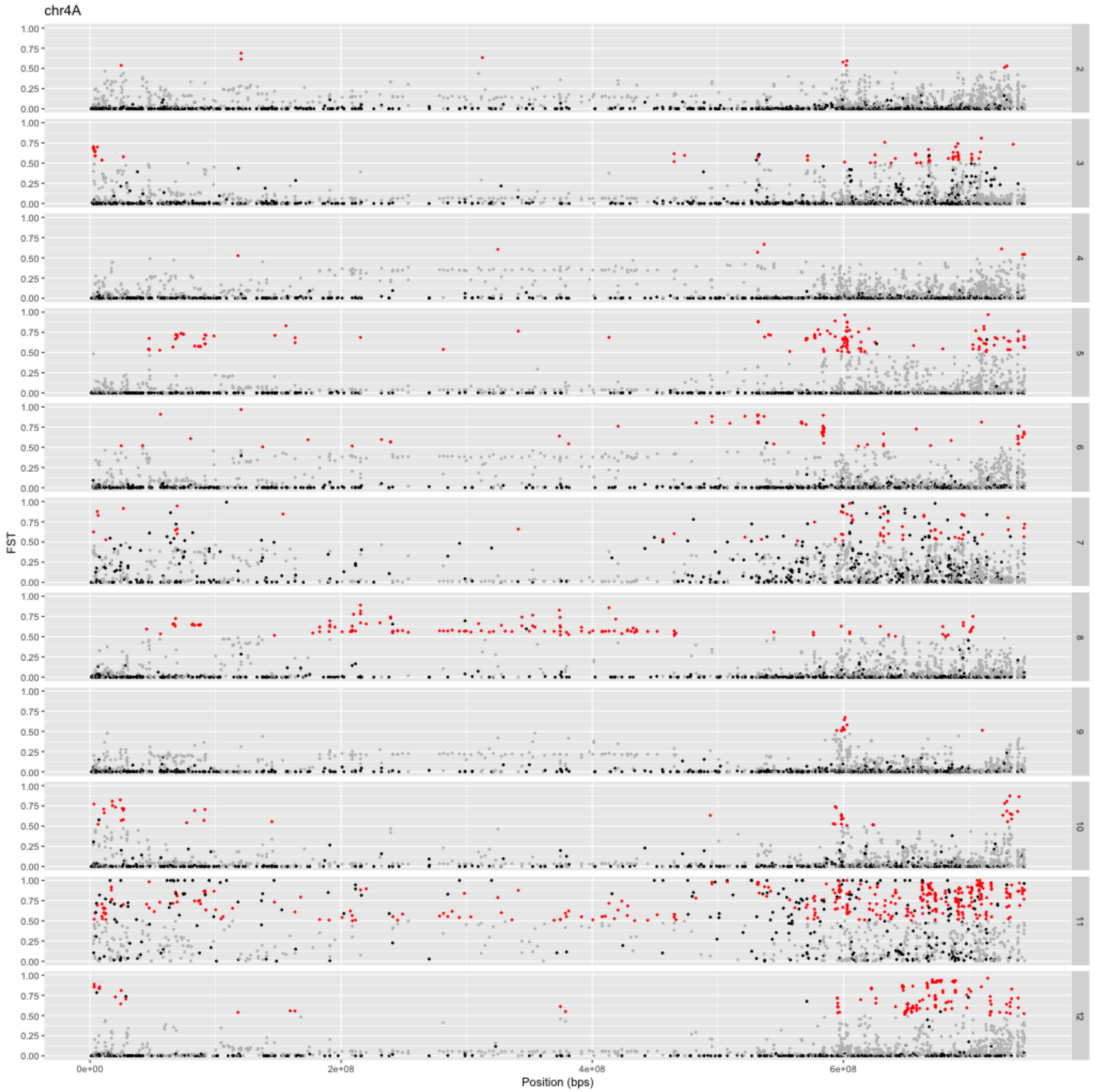

Supplementary Figure 43. Diversity patterns per cluster division in hexaploid.

Analysis of Fst values on a variant per variant basis on chromosome 4A across the 12 clusters divisions.

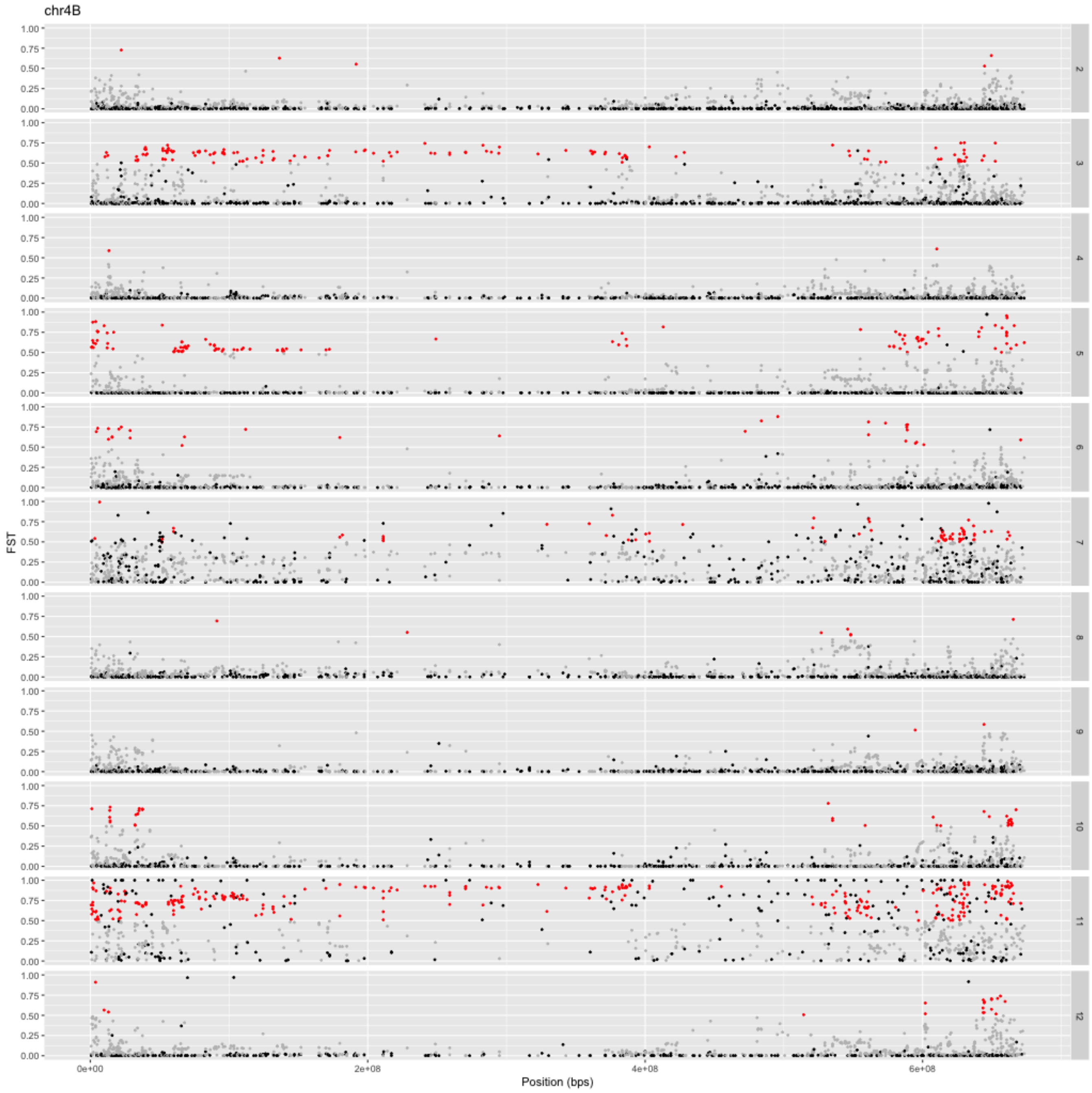

Supplementary Figure 44. Diversity patterns per cluster division in hexaploid.

Analysis of Fst values on a variant per variant basis on chromosome 4B across the 12 clusters divisions.

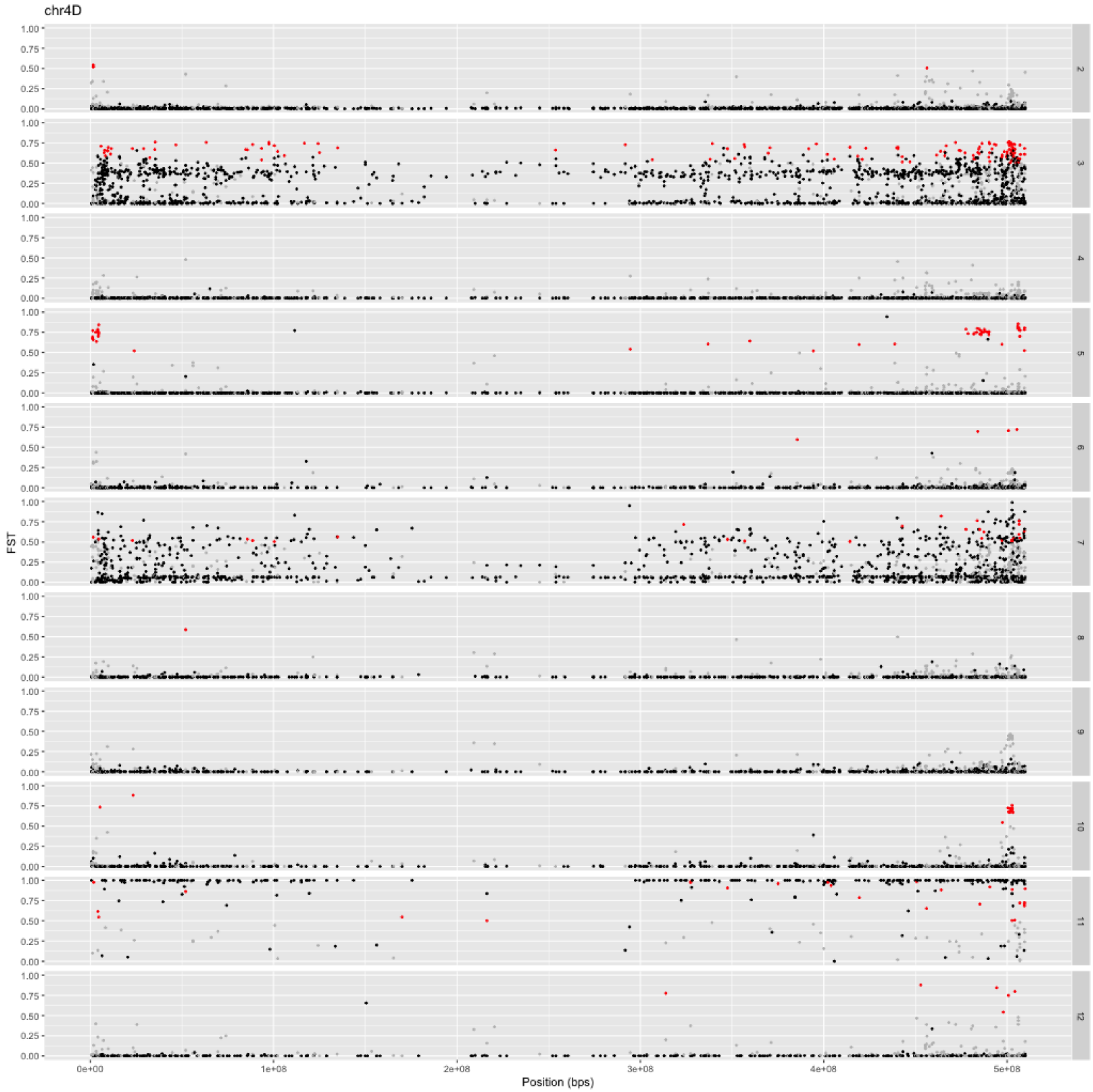

Supplementary Figure 45. Diversity patterns per cluster division in hexaploid.

Analysis of Fst values on a variant per variant basis on chromosome 4D across the 12 clusters divisions.

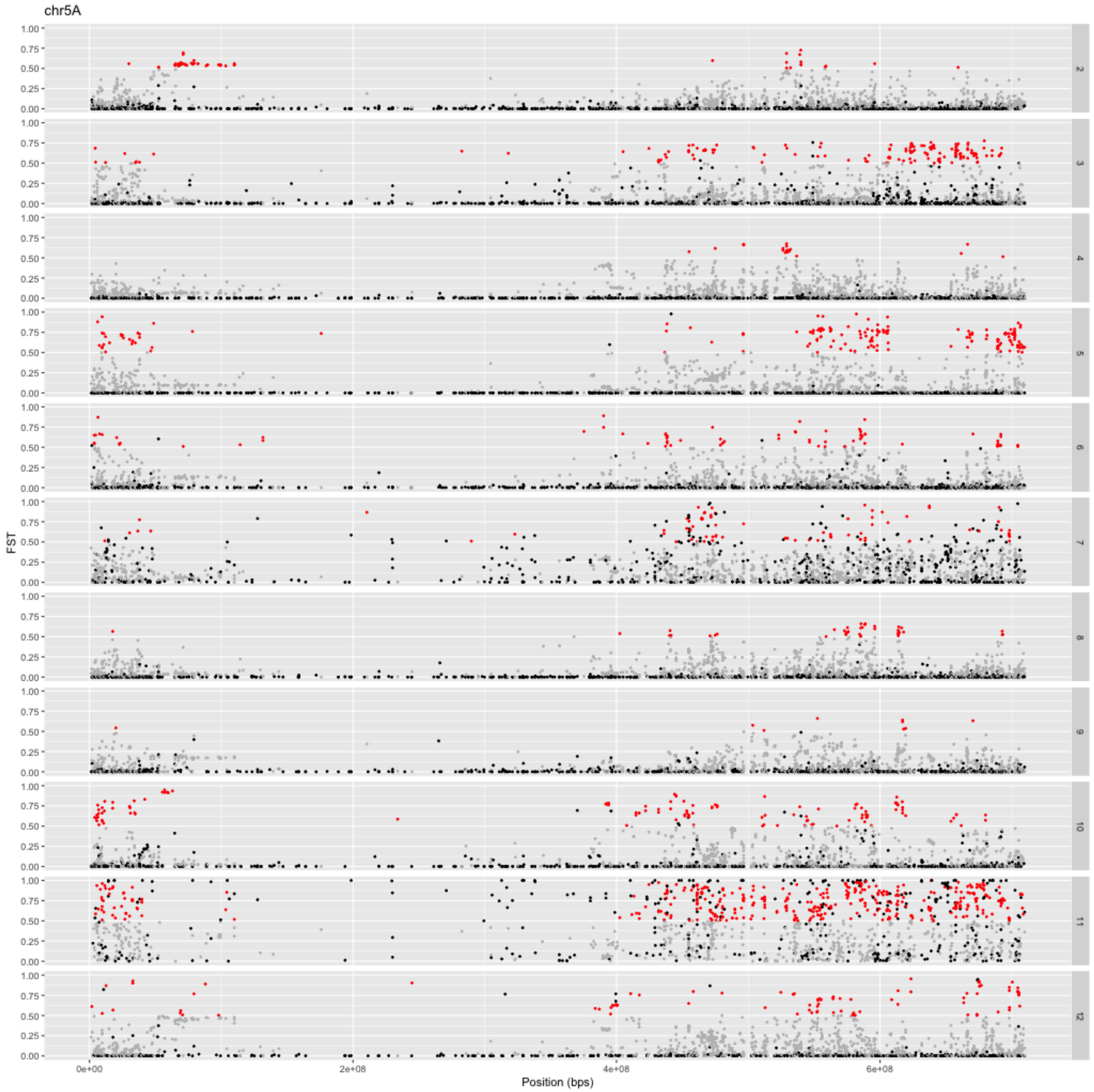

Supplementary Figure 46. Diversity patterns per cluster division in hexaploid.

Analysis of Fst values on a variant per variant basis on chromosome 5A across the 12 clusters divisions.

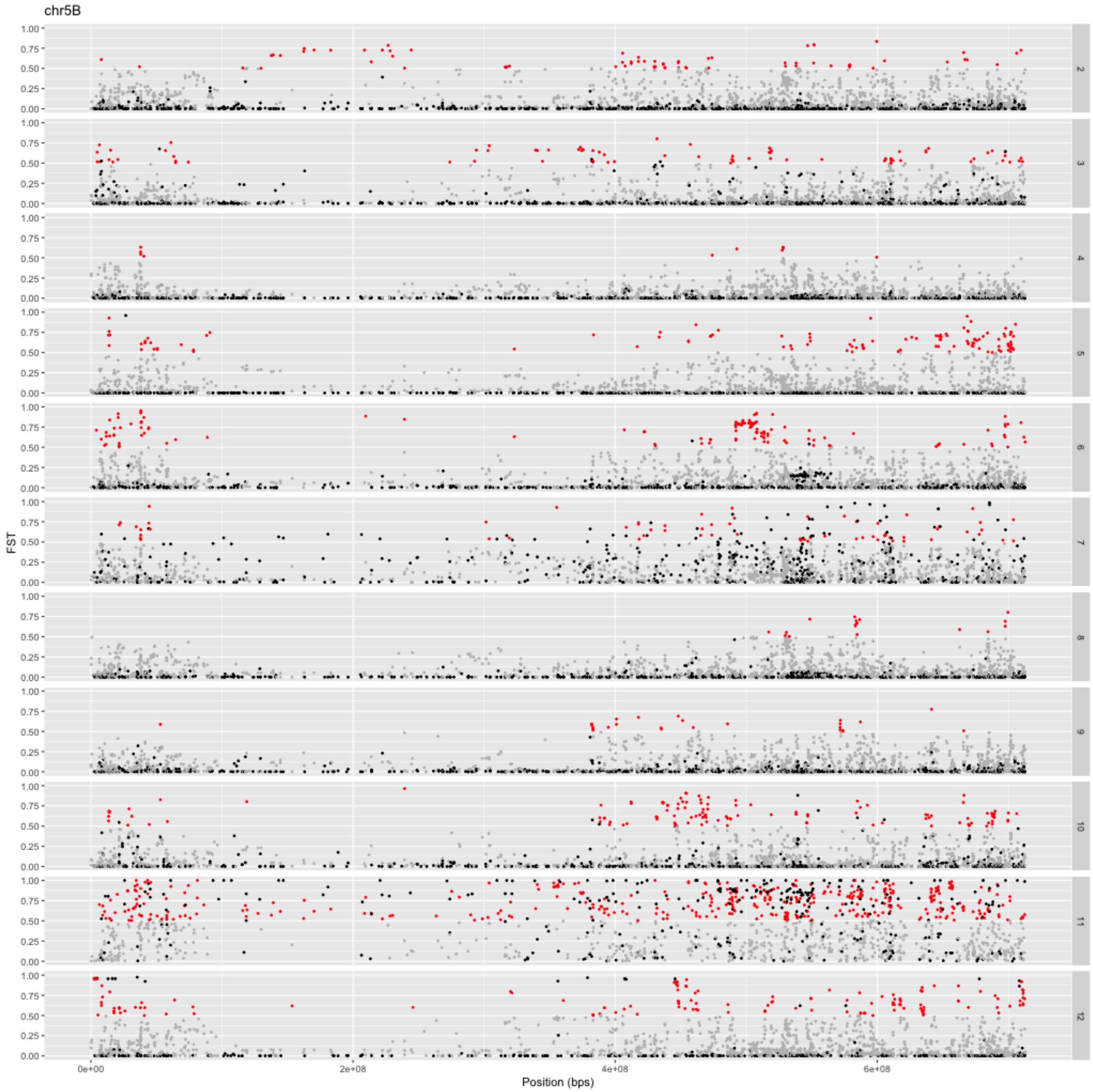

Supplementary Figure 47. Diversity patterns per cluster division in hexaploid.

Analysis of Fst values on a variant per variant basis on chromosome 5B across the 12 clusters divisions.

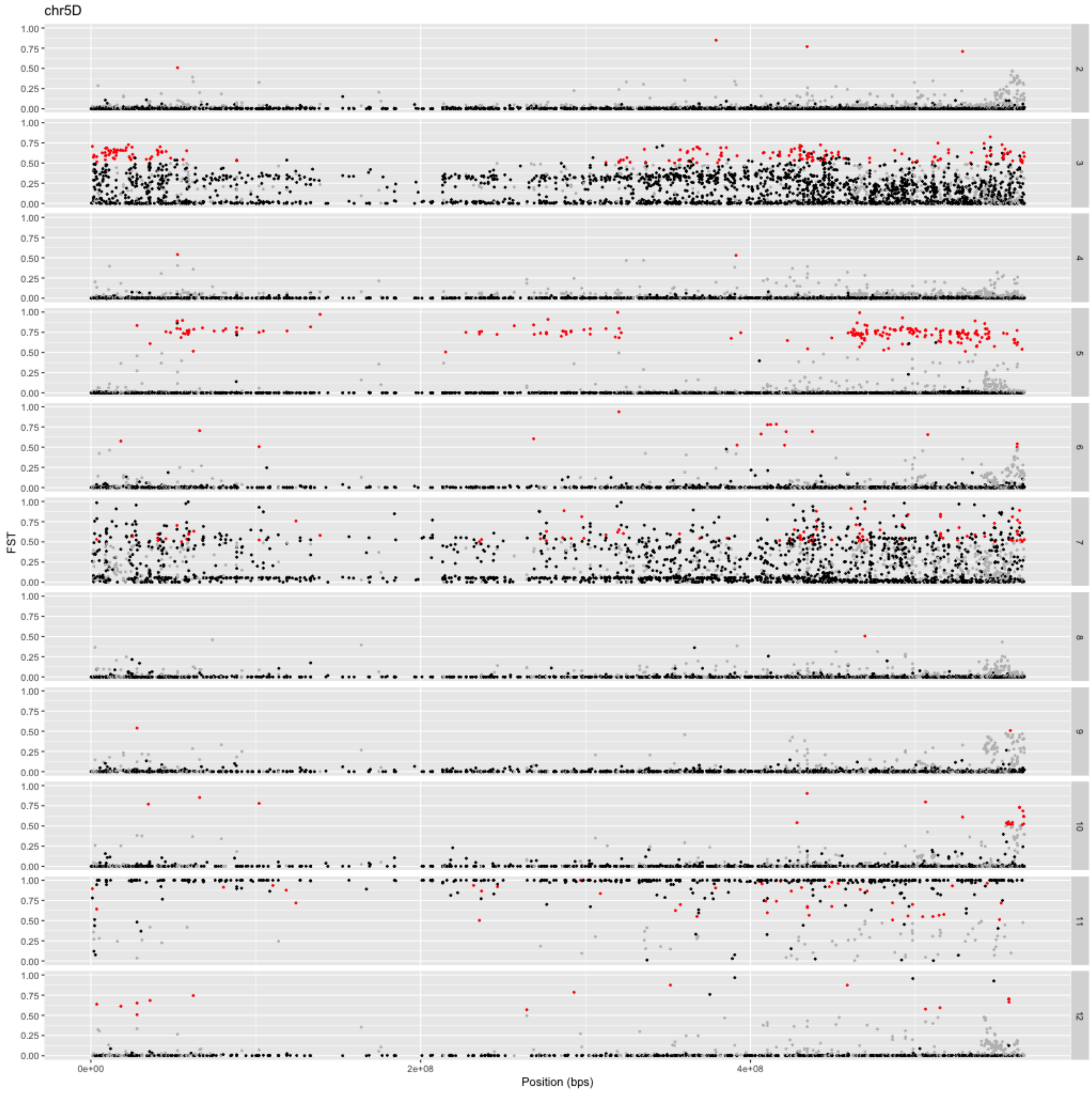

Supplementary Figure 48. Diversity patterns per cluster division in hexaploid.

Analysis of Fst values on a variant per variant basis on chromosome 5D across the 12 clusters divisions.

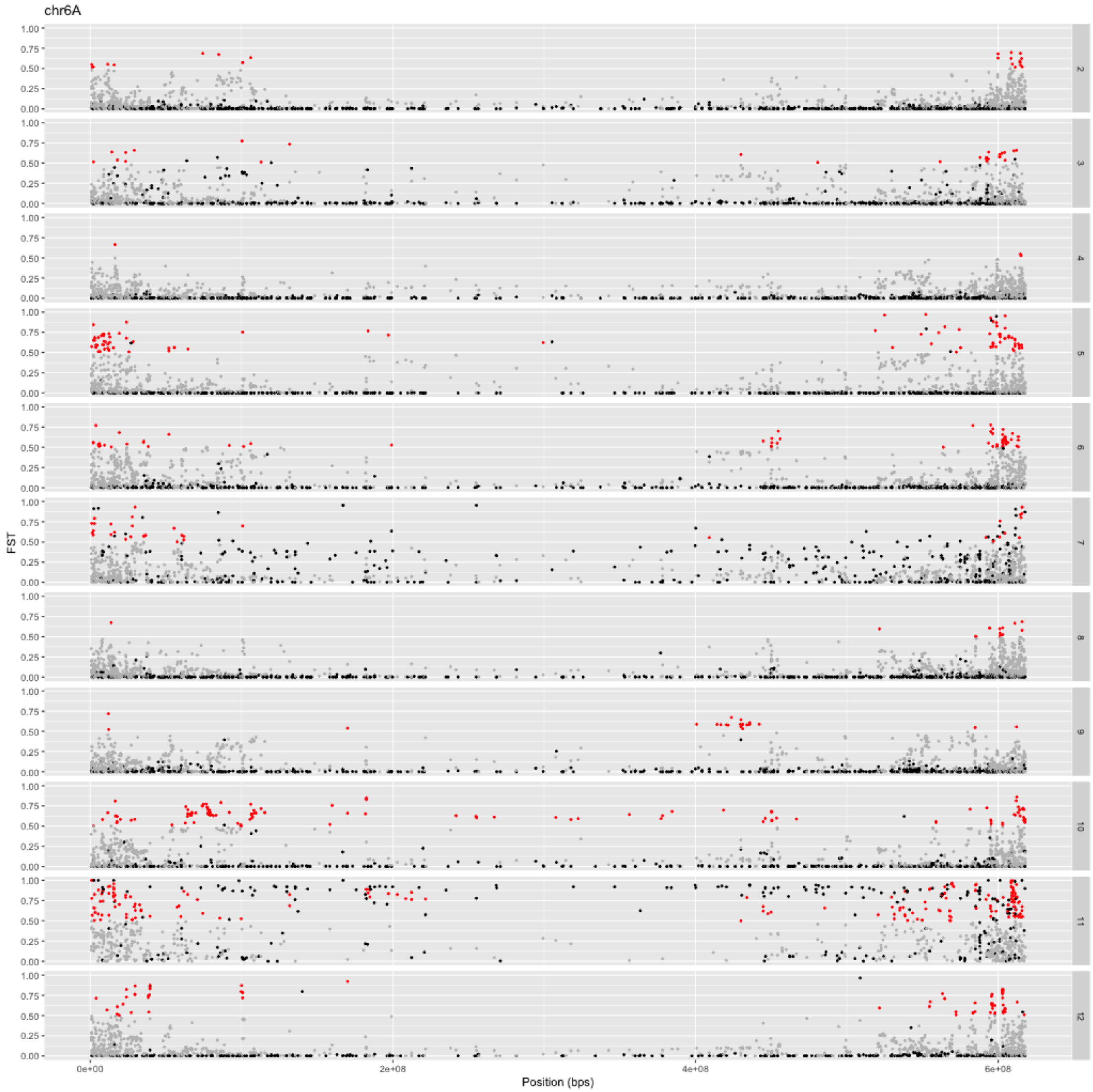

Supplementary Figure 49. Diversity patterns per cluster division in hexaploid.

Analysis of Fst values on a variant per variant basis on chromosome 6A across the 12 clusters divisions.

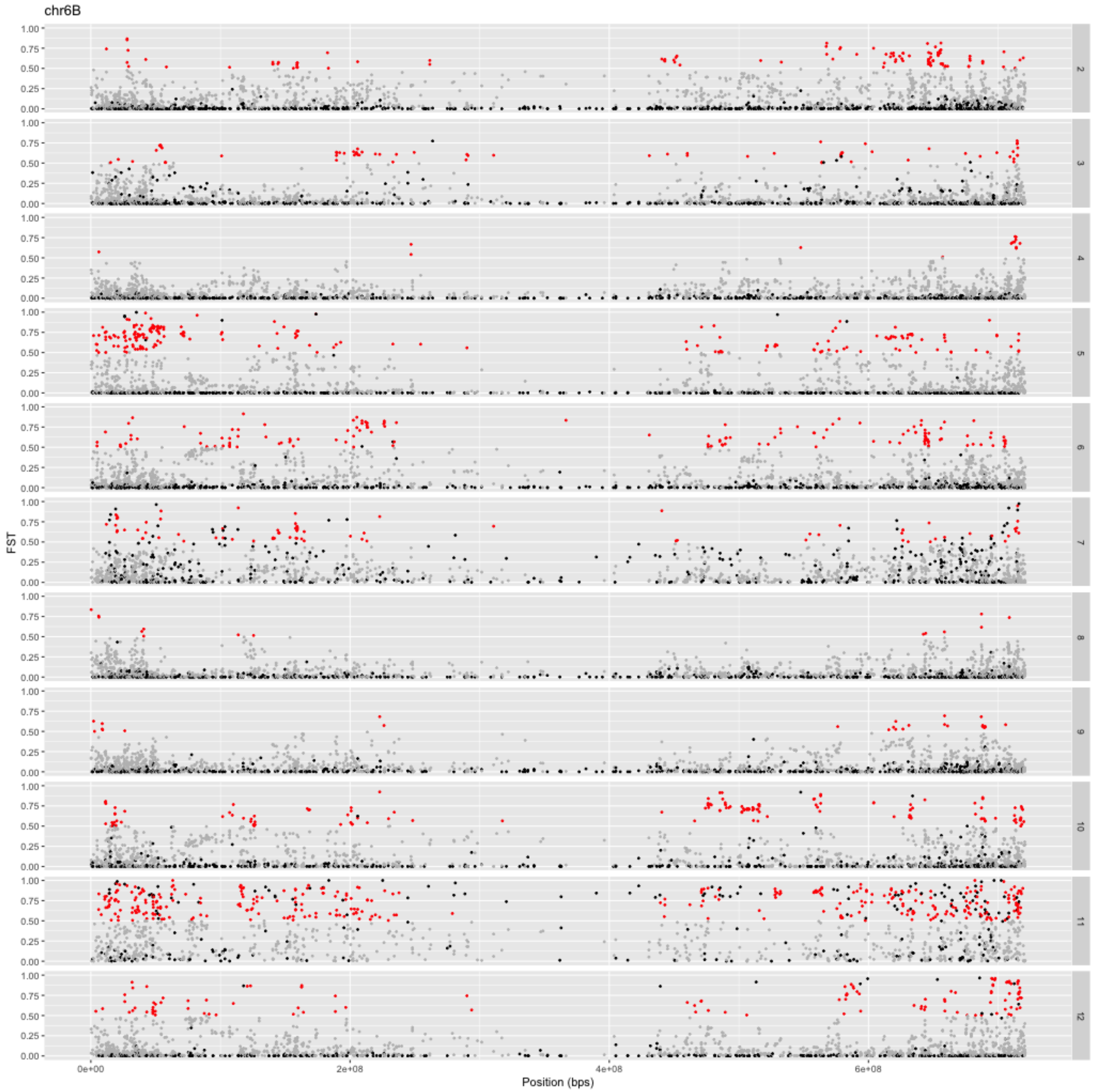

Supplementary Figure 50. Diversity patterns per cluster division in hexaploid.

Analysis of Fst values on a variant per variant basis on chromosome 6B across the 12 clusters divisions.

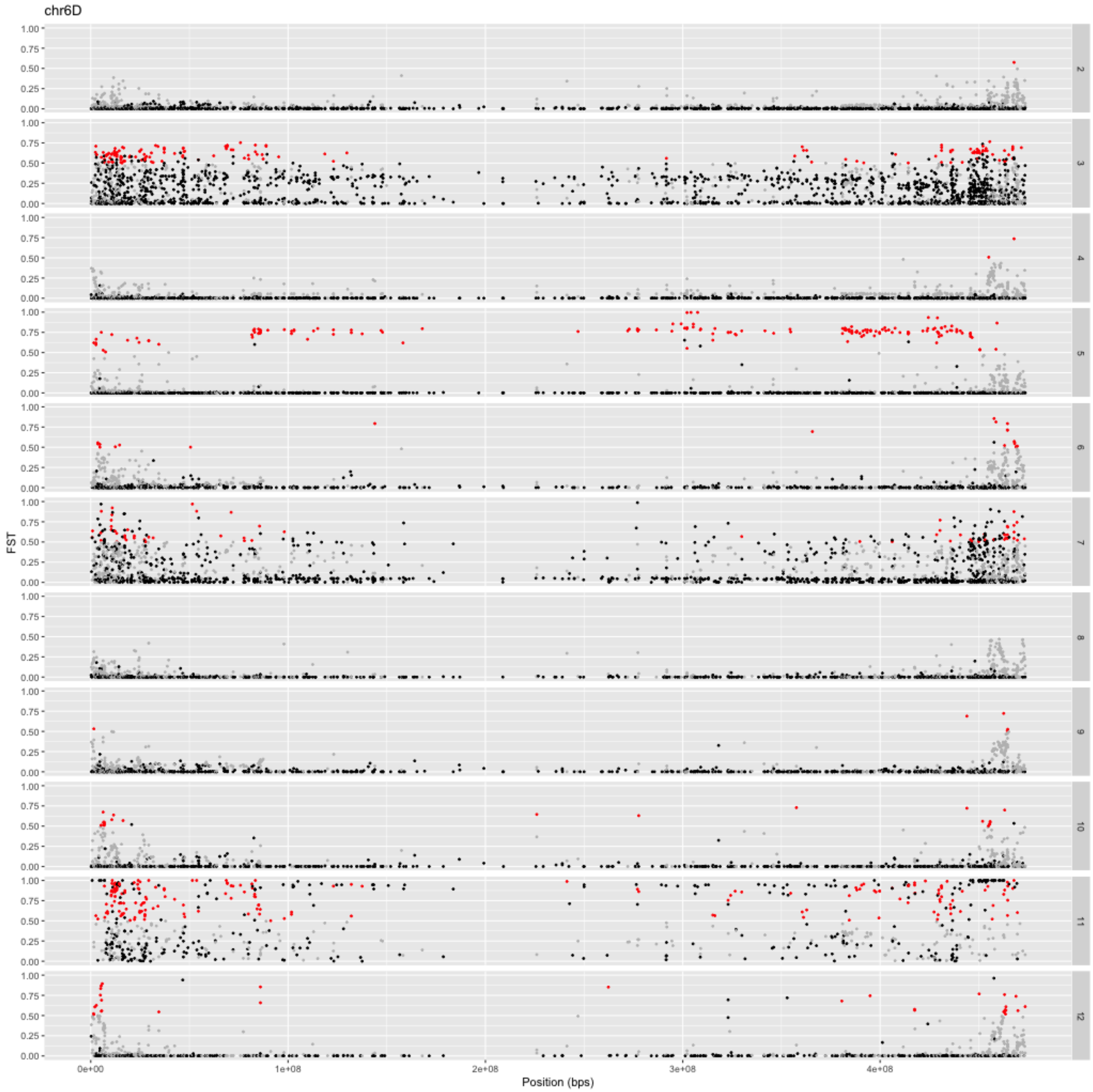

Supplementary Figure 51. Diversity patterns per cluster division in hexaploid.

Analysis of Fst values on a variant per variant basis on chromosome 6D across the 12 clusters divisions.

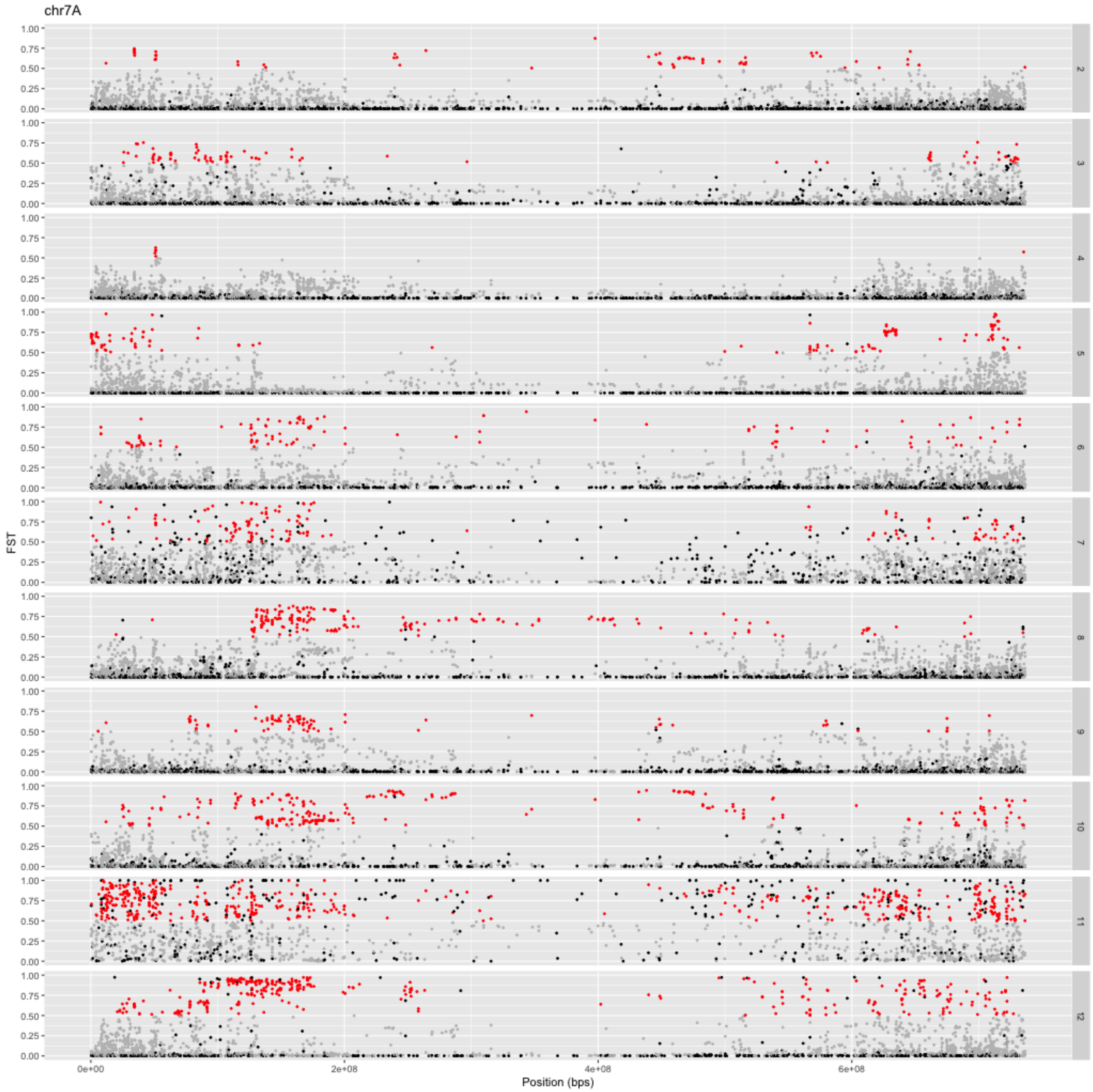

Supplementary Figure 52. Diversity patterns per cluster division in hexaploid.

Analysis of Fst values on a variant per variant basis on chromosome 7A across the 12 clusters divisions.

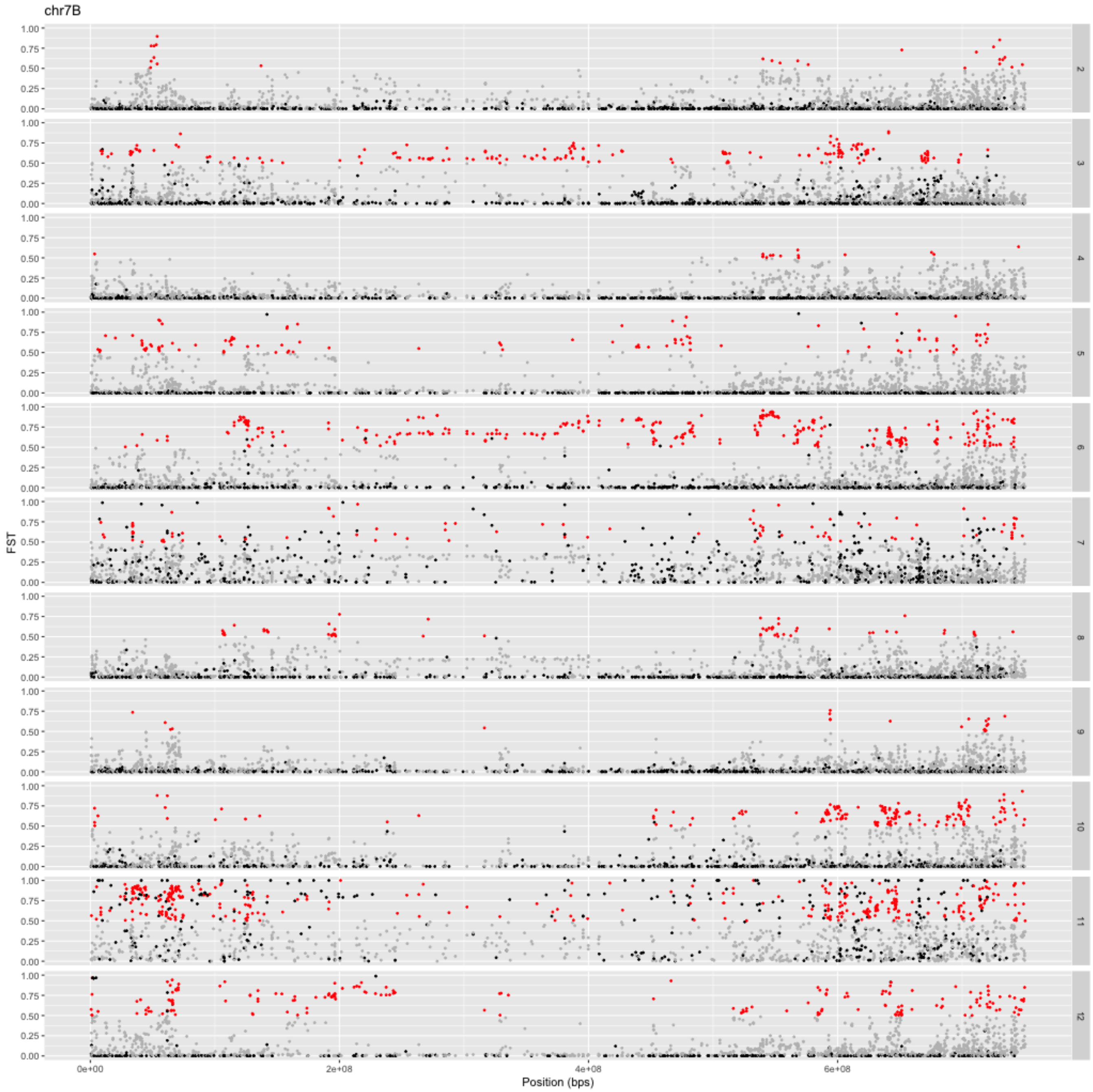

Supplementary Figure 53. Diversity patterns per cluster division in hexaploid.

Analysis of Fst values on a variant per variant basis on chromosome 7B across the 12 clusters divisions.

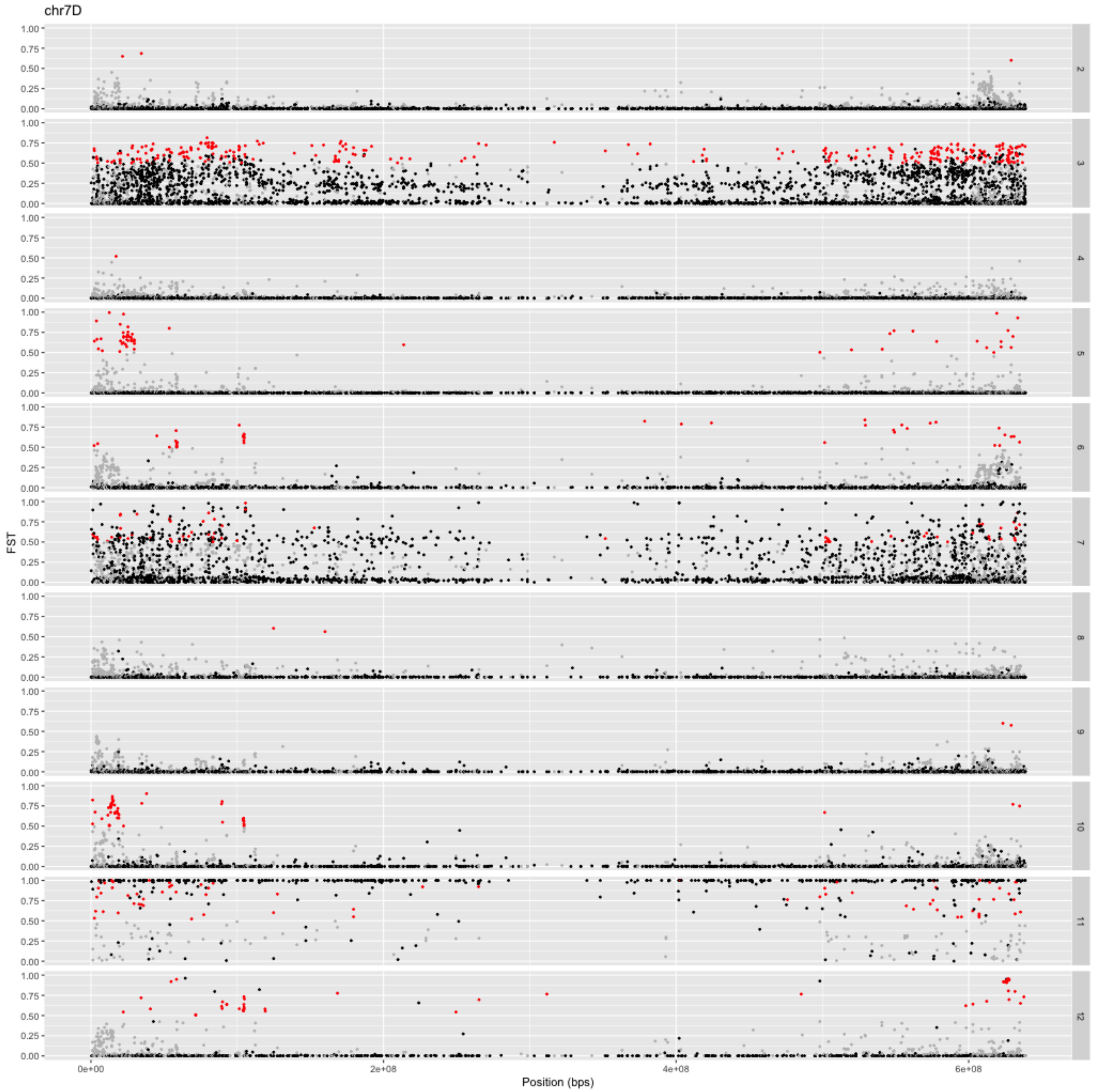

Supplementary Figure 54. Diversity patterns per cluster division in hexaploid.

Analysis of Fst values on a variant per variant basis on chromosome 7D across the 12 clusters divisions.

Supplementary Table 1. Distribution of markers on genome references.

Chrom	# of genes in Refseq v1.0	Markers uniquely aligned in hexaploid	# markers overlapping genes	Markers uniquely aligned in tepraploid	# markers overlapping genes	Markers uniquely aligned in CWR	# markers overlapping genes	Markers uniquely aligned in tepraploid (Svevo)
1A	10790	2747	1296	1593	875	609	359	1738
1B	12768	2886	1092	1977	813	477	249	2158
1D	10486	2876	1825	337	158	2682	2084	
2A	13618	3611	1311	2046	861	820	330	2346
2B	15661	3971	1619	2686	1229	631	335	2955
2D	13428	3616	1697	452	137	3449	2009	
3A	12823	3166	1025	1906	728	677	272	2065
3B	15203	3278	1083	2245	855	487	234	2484
3D	12084	3595	1774	341	137	2896	1888	
4A	12499	2529	707	1512	438	536	242	1607
4B	10185	1814	708	1239	509	427	206	1345
4D	8432	1994	1086	191	59	2307	1591	
5A	13018	3263	940	2020	637	736	346	2112
5B	13851	2984	923	2049	718	533	269	2247
5D	12341	3479	1758	356	127	3145	2083	
6A	10453	2323	966	1421	648	558	312	1573
6B	13000	2896	1156	2015	897	466	233	2200
6D	9312	2463	1633	325	130	2108	1669	
7A	13829	4147	1743	2608	1287	667	329	2767
7B	13390	3001	1033	2142	783	487	274	2331
7D	13041	4370	1990	479	149	3102	1824	
Unk		1058	724	866	346	254	213	1253
Total	260212	66067	28089	30806	12521	28054	17351	31181

Distribution of the uniquely aligned SNP and SilicoDArT markers generated for the hexaploid, tepraploid and CWR on the genome references Chinese spring IWGSC RefSeq assembly v1.0 and Durum wheat genome reference (Svevo). In the Refseq v1.0 we also aligned the markers on the annotation genes resulting in the number of markers located within genes and in close proximity to genes (within 10Kb).

Supplementary Table 2. Distribution of markers on genetic map.

Chrom	# of markers in DArT genetic map	SNPs hexaploid	SNPs tepraploid	SNPs CWR	SilicoDArT hexaploid	SilicoDArT tepraploid	SilicoDArT CWR
1A	4460	2152	1493	946	984	892	654
1B	9060	3948	2426	2333	1844	1794	1588
1D	2702	1221		607	592		752
2A	5782	2722	1826	1083	1377	1178	922
2B	12265	5176	3278	2957	2547	2490	2443
2D	4674	1750		1019	1290		1545
3A	4513	2189	1518	598	1037	1019	603
3B	8356	3349	2276	1287	1841	1963	1128
3D	3308	1466		578	510		964
4A	5281	1912	1335	732	1239	1065	635
4B	2312	970	756	311	538	594	275
4D	1081	569		318	125		422
5A	3448	1783	1243	543	874	707	506
5B	7050	3076	2052	1246	1978	1787	1229
5D	1460	638		221	298		408
6A	5134	1942	1325	837	1151	1031	868
6B	6159	2250	1597	716	1549	1449	790
6D	2652	1240		517	424		884
7A	5864	2457	1765	637	1487	1249	702
7B	5709	1813	1295	576	1422	1493	613
7D	3852	1878		676	464		1091
Total	105122	44501	24185	18738	23571	18711	19022

Distribution of the uniquely aligned SNP and SilicoDArT markers generated for the hexaploid, tepraploid and CWR on DArT consensus maps v4.

Supplementary Table 3. Taxonomy of hexaploid.

Taxonomy	# of samples
Triticum aestivum L. aestivum	55879
Triticum aestivum L. sphaerococcum	13
Triticum aestivum L. compactum	15
Triticum aestivum L. spelta	22
Triticum aestivum L. macha	10
Triticum hybr.	122
x Triticosecale spp.	141
x Aegilotriticum spp.	140

Distribution of accessions in the 8 domesticated hexaploid species included in the analysis.

Supplementary Table 4. Taxonomy of tetraploid.

Taxonomy	# of samples
T. durum Desf.	14,705
T. dicoccum Schrank ex Schubler	83
T. carthlicum Nevski in Kom	116
T. turanicum Jakubz.	11
T. turgidum L.	57
T. karamyschevii Nevski	2
T. polonicum L.	5
T. aethiopicum Jakubz	2,822
Undetermined spp.	1,145

Distribution of accessions in the 8 domesticated tetraploid species included in the analysis.

Supplementary Table 5. Taxonomy of crop wild relatives.

Section	Diploid Species	Genome	# Acc.	# outliers	Tetraploid Species	Genome	# Acc.	# outliers	Hexaploid Species	Genome	# Acc.	# outliers
Aegilops L.	*Ae. umbellulata Zhuk.*	U	70	7	*Ae. biuncialis Vis.*	UM	311	20				
					Ae. columnaris Zhuk.	UM	76	6				
					Ae. geniculata Roth	MU	394	7				
					Ae. kotschyi Boiss.	SU	63	2				
					Ae. neglecta Req. ex Bertol.	UM	49	3	*Ae. neglecta Req. ex Bertol.*	UMN	77	4
					Ae. peregrina (Hackel) Maire et Weiller	SU	186	7				
					Ae. triuncialis L.	UC	631	30				
Comopyrum (Jaub. et Spach) Zhuk.	*Ae. comosa Sibth. et Sm.*	M	25	7								
	Ae. uniaristata Vis.	N	5									
Cylindropyron (Jaub. et Spach) Zhuk.	*Ae. markgrafii (Greuter) Hammer*	C	49	7	*Ae. cylindrica Host*	DC	330	21				
Sitopsis (Jaub. et Spach) Zhuk	*Ae. bicornis (Forssk.) Jaub. et Sp.*	S^b	16	3								
	Ae. longissima Schweinf. et Muschl.	S^l	23	0								
	Ae. sharonensis Eig	S^{sh}	2									
	Ae. searsii Feldman et Kislev ex Hammer	S^s	34	8								
	Ae. speltoides Tausch	S	124	21								
Vertebrata Zhuk. emend. Kihara	*Ae. tauschii Coss.*	D	952	22	*Ae. crassa Boiss. (4x)*	DM	27	4	*Ae. crassa Boiss. (6x)*	DDM	57	5
					Ae. ventricosa Tausch	DN	42	4	*Ae. vavilovii (Zhuk.) Chennav.*	DMS	65	4
									Ae. juvenalis (Thell.) Eig	DMU	14	1
Subgenus Amblyopyrum	*Ae. mutica Boiss.*	T	10	1								
Triticum	*T. boeoticum Boiss.*	A^b	19	2	*T. dicoccoides (Körn. ex Asch. et Graebner) Schweinf.*	BBA^uA^u	15	0	*T. zhukovskyi Menabde et Ericzjan*	$GGA^uA^uA^bA^b$	2	1
	T. urartu Tumanian ex Gandilyan	A^u	26	2	*T. araraticum Jakubz*	GGA^uA^u	13	2				

CWR taxa investigated. Genomic formulas of tetraploids and hexaploids are cited as “female x male parent.” Underlining indicates modification of the same genome as present in the diploid species. Genome (G) symbols are according to Kimber and Tsunewaki (1988). #Acc, number of accessions considered for analysis. #outliers, number of accessions not considered. nd – not determined

Supplementary Table 6. GWAS for grain protein content and sedimentation traits.

Trait	CloneID	Chromosome	Position	P.value	maf	FDR	REF	ALT	Gene_Name
GPC	103182316	2A	775734046	9.74E-07	0.278275	3.33E-03	A	T	TraesCS2A01G584300-TraesCS2A01G584400-E7
GPC	103059177	2D	33241377	1.75E-06	0.106751	3.82E-03	C	T	TraesCS2D01G077800
GPC	102497386	3B	737534352	1.33E-06	0.297605	3.43E-03	T	A	TraesCS3B01G491200-TraesCS3B01G491300-E4
GPC	103063052	4A	597887286	1.46E-08	0.106319	4.49E-04	T	G	TraesCS4A01G299600
GPC	103063051	4B	9947172	4.11E-08	0.207732	6.32E-04	C	T	TraesCS4B01G014000
GPC	101550822	5A	552869163	1.27E-06	0.35704	3.43E-03	G	A	TraesCS5A01G350100-TraesCS5A01G350200-E10
GPC	103136391	5A	592515888	9.83E-06	0.4221	1.51E-02	A	G	TraesCS5A01G398700
GPC	102959599	5A	661222304	3.74E-07	0.340883	3.33E-03	A	G	TraesCS5A01G492400
GPC	102378423	5B	531550184	4.61E-06	0.355309	7.47E-03	G	A	TraesCS5B01G351000
GPC	103011120	5B	533748239	4.44E-06	0.306117	7.47E-03	C	G	TraesCS5B01G354800
GPC	102895868	5D	437451309	1.45E-06	0.341171	3.43E-03	C	G	TraesCS5D01G356100-TraesCS5D01G356200-E1
GPC	102437914	5D	557778049	9.69E-07	0.289094	3.33E-03	T	C	TraesCS5D01G552200
GPC	103233603	5D	559393264	1.41E-06	0.359492	3.43E-03	A	G	TraesCS5D01G555700-TraesCS5D01G555800-E1
GPC	102434870	6A	74845686	4.62E-06	0.220716	7.47E-03	T	G	TraesCS6A01G106700-TraesCS6A01G106800-E1
GPC	102489895	6A	602970478	1.86E-06	0.350548	3.82E-03	A	G	TraesCS6A01G384600-TraesCS6A01G384700-E1
GPC	102302737	6B	50914126	2.03E-06	0.341604	3.90E-03	A	G	TraesCS6B01G074100-TraesCS6B01G074200-E1
GPC	102723096	7A	4966351	5.31E-07	0.478217	3.33E-03	C	G	TraesCS7A01G011200
GPC	103204957	7B	679452742	9.67E-07	0.24221	3.33E-03	T	C	TraesCS7B01G410100-TraesCS7B01G410200-E1
SDS	103017094	1A	118442761	4.93E-08	0.117426	7.29E-05	T	C	TraesCS1A01G114200-TraesCS1A01G114300-E1
SDS	103164307	1B	17593163	2.80E-08	0.135747	5.38E-05	T	C	TraesCS1B01G036800-TraesCS1B01G036900-E1
SDS	103135096	1B	39766159	1.07E-08	0.111945	3.62E-05	G	C	TraesCS1B01G057500-TraesCS1B01G057600-E1
SDS	102815651	1B	57520154	1.27E-09	0.113531	9.75E-06	G	C	TraesCS1B01G073700-TraesCS1B01G073800-E1
SDS	102963755	1B	117118137	3.83E-08	0.114686	6.92E-05	G	C	TraesCS1B01G105100-TraesCS1B01G105200-E8
SDS	103142774	1B	121517174	4.68E-08	0.108771	7.29E-05	G	T	TraesCS1B01G109400-TraesCS1B01G109500-E1
SDS	102985679	1B	131171433	4.15E-09	0.113099	2.12E-05	T	C	TraesCS1B01G112800-TraesCS1B01G112900-E1
SDS	103102710	1B	136364939	2.28E-10	0.123918	3.51E-06	T	C	TraesCS1B01G115900-TraesCS1B01G116000-E2
SDS	103177447	1B	138573230	2.36E-09	0.110502	1.45E-05	A	G	TraesCS1B01G118300
SDS	103113016	1B	145574681	1.18E-08	0.110069	3.62E-05	A	C	TraesCS1B01G122000-TraesCS1B01G122100-E1
SDS	102731575	1B	160207738	2.28E-08	0.11281	4.67E-05	C	A	TraesCS1B01G129700-TraesCS1B01G129800-E3
SDS	102884002	1B	170843101	4.76E-08	0.113531	7.29E-05	C	T	TraesCS1B01G1344
SDS	102792770	1B	183927289	7.43E-10	0.114253	7.62E-06	A	G	TraesCS1B01G139600-TraesCS1B01G139700-E1
SDS	103139652	1B	189030863	1.52E-08	0.113387	3.90E-05	C	T	TraesCS1B01G141500-TraesCS1B01G141600-E1
SDS	103020085	1D	66381523	2.26E-08	0.136324	4.67E-05	G	A	TraesCS1D01G083400-TraesCS1D01G083500-E1
SDS	102915312	1D	91239214	7.79E-09	0.060877	3.42E-05	C	T	TraesCS1D01G102700
SDS	103123896	1D	93129941	9.19E-09	0.112377	3.53E-05	G	T	TraesCS1D01G103800

SDS	103124125	1D	111837904	2.05E-08	0.059146	4.67E-05	C	G	TraesCS1D01G116000
SDS	102943363	2A	691480242	1.38E-08	0.060012	3.86E-05	A	G	TraesCS2A01G441600

List of QTL identify in the Genome-wide association analysis for Gran protein content and Sedimentation traits. It was performed using an iterative usage of fixed and random model circulating probability implemented by R software with correction of kinship, including the first three PCA values as fixed effects. The test was adjusted for multiple comparison in terms of P-value cut off determination and FDR calculation.
