# Supplementary material for: Diversity analysis of 80,000 wheat accessions reveals consequences and opportunities of selection footprints
Source: Nat Commun. 2020 Sep 11;11:4572. doi: 10.1038/s41467-020-18404-w (PMC7486412; doi:10.1038/s41467-020-18404-w)
Supplement: Supplementary file 3 — Reporting Summary [file 41467_2020_18404_MOESM3_ESM.pdf]

nature research

Corresponding author(s): Carolina Sansaloni

Last updated by author(s): 08/01/2020

Reporting Summary

Nature Research wishes to improve the reproducibility of the work that we publish. This form provides structure for consistency and transparency in reporting. For further information on Nature Research policies, see Authors & Referees and the Editorial Policy Checklist .

Statistics

For all statistical analyses, confirm that the following items are present in the figure legend, table legend, main text, or Methods section.

n/a	Confirmed	
☐	☒	The exact sample size (*n*) for each experimental group/condition, given as a discrete number and unit of measurement
☐	☒	A statement on whether measurements were taken from distinct samples or whether the same sample was measured repeatedly
☐	☒	The statistical test(s) used AND whether they are one- or two-sided<br>*Only common tests should be described solely by name; describe more complex techniques in the Methods section.*
☐	☒	A description of all covariates tested
☒	☐	A description of any assumptions or corrections, such as tests of normality and adjustment for multiple comparisons
☐	☒	A full description of the statistical parameters including central tendency (e.g. means) or other basic estimates (e.g. regression coefficient) AND variation (e.g. standard deviation) or associated estimates of uncertainty (e.g. confidence intervals)
☐	☒	For null hypothesis testing, the test statistic (e.g. *F*, *t*, *r*) with confidence intervals, effect sizes, degrees of freedom and *P* value noted<br>*Give P values as exact values whenever suitable.*
☒	☐	For Bayesian analysis, information on the choice of priors and Markov chain Monte Carlo settings
☐	☒	For hierarchical and complex designs, identification of the appropriate level for tests and full reporting of outcomes
☒	☐	Estimates of effect sizes (e.g. Cohen's *d*, Pearson's *r*), indicating how they were calculated

Our web collection on statistics for biologists contains articles on many of the points above.

Software and code

Policy information about availability of computer code

Data collection	No software was used
Data analysis	The CurlyWhirly visualization tool is described and available for download at https://ics.hutton.ac.uk/curlywhirly/ and the underlying code are available at: https://github.com/cropgeeks/curlywhirly<br>The Flapjack analysis and visualization tool is described and available for download at https://ics.hutton.ac.uk/flapjack/ and the underlying code are available at: https://github.com/cropgeeks/flapjack<br>Free R software environment used for the diversity and clustering analysis are available at: https://github.com/jfranco1951/Genetic-Diversity.

For manuscripts utilizing custom algorithms or software that are central to the research but not yet described in published literature, software must be made available to editors/reviewers. We strongly encourage code deposition in a community repository (e.g. GitHub). See the Nature Research guidelines for submitting code & software for further information.

Data

Policy information about availability of data

All manuscripts must include a data availability statement. This statement should provide the following information, where applicable:

- Accession codes, unique identifiers, or web links for publicly available datasets
- A list of figures that have associated raw data
- A description of any restrictions on data availability

IIn this study, we used visualization tools and interactive data repository to handle the large data volumes generated. Data supporting the findings of this work is available in the CIMMYT Dataverse repository with the identifier: http://hdl.handle.net/11529/10548030 and Supplementary Information files. We also stored the same data into Germinate [http://germinate.cimmyt.org/wheat/], a plant genetic resources database that offers facilities to store both standard collection information and passport data along with more advanced data types such as genotypic data. The data can be explored in an interactive way allowing users to select

subset of information using different filtering criteria such as country of origin, geolocation regions, biological status, etc. The data sets created in Flapjack or Germinate can be visualized using CurlyWhirly (https://ics.hutton.ac.uk/curlywhirly), a tool that we hereby release for use by the research community. The source data underlying Figures 1, 2, 3. 4 and 5 are provided as a Source Data file. All biological material is available at the CIMMYT and ICARDA germplasm bank upon on-line request at https://www.cimmyt.org/resources/seed-request/ and https://www.icarda.org/. Records for all germplasm accessions with DOIs included in this study can also be accessed through the Global Information System of the International Treaty on Plant Genetic Resources for Food and Agriculture at https://ssl.fao.org/glis/. Data publicly available used in this manuscript: IWGSC RefSeq v1.0 https://wheat-urgi.versailles.inra.fr/Seq-Repository/Assemblies, Durum wheat genome (cv. Svevo) https://www.interomics.eu/durum-wheat-genome.

Field-specific reporting

Please select the one below that is the best fit for your research. If you are not sure, read the appropriate sections before making your selection.

☒ Life sciences ☐ Behavioural & social sciences ☐ Ecological, evolutionary & environmental sciences

For a reference copy of the document with all sections, see nature.com/documents/nr-reporting-summary-flat.pdf

Life sciences study design

All studies must disclose on these points even when the disclosure is negative.

Sample size	We explored a total of 79,191 wheat samples. The samples size was not calculated, it was based on the availability of accession from the Germplasm Bank collection.
Data exclusions	No data was excluded from the analysis
Replication	20 % of the samples are always genotype as replicates and the results are compared and used to calculate the reproducibility of the data. We only consider in this study data with more than 99% of similarity.
Randomization	The study was divided in three groups, Crop wild relatives (including all wheat wild species), domesticated tetraploid and domesticated hexaploid.
Blinding	For the GWAS analysis, phenotypic data were collected without knowledge of passport records or genetic data.

Reporting for specific materials, systems and methods

We require information from authors about some types of materials, experimental systems and methods used in many studies. Here, indicate whether each material, system or method listed is relevant to your study. If you are not sure if a list item applies to your research, read the appropriate section before selecting a response.

Materials & experimental systems

n/a	Involved in the study
☒	☐ Antibodies
☒	☐ Eukaryotic cell lines
☒	☐ Palaeontology
☒	☐ Animals and other organisms
☒	☐ Human research participants
☒	☐ Clinical data

Methods

n/a	Involved in the study
☒	☐ ChIP-seq
☒	☐ Flow cytometry
☒	☐ MRI-based neuroimaging
