# Supplementary material for: Diversity analysis of 80,000 wheat accessions reveals consequences and opportunities of selection footprints
Source: Nat Commun. 2020 Sep 11;11:4572. doi: 10.1038/s41467-020-18404-w (PMC7486412; doi:10.1038/s41467-020-18404-w)
Supplement: Supplementary file 4 — Description of Additional Supplementary Files [file 41467_2020_18404_MOESM4_ESM.pdf]

Description of Additional Supplementary Files

Supplementary Data 1
Passport information of germplasm accessions included in this study. The file is divided in three sheets, hexaploid, tetraploid and CWR material.

Supplementary Data 2
List of markers ID, sequence of the marker, physical genome position and annotation of genes in the wheat reference genome. The file is divided in three sheets, hexaploid, tetraploid and CWR.

Supplementary Data 3
Genetic consensus map v4 including 105,122 markers divided in 21 chromosomes and the sequence of each marker.

Supplementary Data 4
Inputfile_for Curlywhirly software of Hexaploid dataset.

Supplementary Data 5
Inputfile_for Curlywhirly software of Tetraploid dataset.
Supplementary Data 6: Inputfile_for Curlywhirly software of CWR dataset.

Supplementary Movie 1
3D Multidimensional scaling plot visualized in Curlywhirly of 56,342 domesticated hexaploid accession with 66,067 SNP markers differentiated by biological status based on passport information (elite, landraces, cultivar, synthetic, etc).

Supplementary Movie 2
3D Multidimensional scaling plot visualized in Curlywhirly of 56,342 domesticated hexaploid accession distributed in 12 clusters based on MRD and clustering analysis.

Supplementary Movie 3
3D Multidimensional scaling plot visualized in Curlywhirly of 18,946 domesticated tetraploid accessions with 30,806 SNP markers differentiated by biological status based on passport information.

Supplementary Movie 4
3D Multidimensional scaling plot visualized in Curlywhirly of 18,946 domesticated tetraploid accession distributed in 7 clusters based on MRD and clustering analysis.

Supplementary Movie 5
3D Multidimensional scaling plot visualized in Curlywhirly of 3,903 CWR accessions based on Jaccard distance using 61,505 SilicoDArT markers including the 27 wild relative species.
