# Supplementary material for: Diversity analysis of 80,000 wheat accessions reveals consequences and opportunities of selection footprints
Source: Nat Commun. 2020 Sep 11;11:4572. doi: 10.1038/s41467-020-18404-w (PMC7486412; doi:10.1038/s41467-020-18404-w)
Supplement: Supplementary file 2 — Peer Review [file 41467_2020_18404_MOESM2_ESM.pdf]

Reviewers' comments:

Reviewer #1 (Remarks to the Author):

In this manuscript, Sansaloni and colleagues present the results of a large-scale genotyping effort on wheat accessions from the CIMMYT and ICARDA germplasm collections. The biggest novelty of the study is the sheer scale of the data set. In total, the authors genotyped close to 80,000 wheat accessions and I am not aware of a larger genotyping effort for a germplasm collection. This effort clearly shows that the transition to digital germplasm collections is now feasible. As such, the topic and scale of the analyses presented here are timely and highly relevant. I particularly liked the visualization of the data in CurlyWhirly, which is innovative and cool. The results section however does not go beyond a basic population genomics description of the data set. Unfortunately, there is no information in the results on how this genomic information can now be utilized for breeding. Given the 'big promises' made in the title this was slightly disappointing. For example, is there historic phenotypic information for some of the accessions that could be utilized for a GWAS? Such an additional analysis would be highly valuable to demonstrate that this data set is useful to dissect traits and to transfer beneficial alleles from landraces into elite material by using the markers described in this study. Also, the results section is very repetitive, since the same analyses are essentially presented three times for hexaploid, tetraploid, and wild wheats, respectively.

Another important aspect is accessibility of data for breeders. Unfortunately, the two links provided in the 'data and materials availability' section are not yet active. For example, is it possible to retrieve the accession names from the multidimensional scaling plots in CurlyWhirly by simply clicking on one of the dots?

A last major concern is the choice of genotyping method, or more precisely, the use of a proprietary software for variant calling. There are numerous open source tools available to handle this kind of sequencing data and it is very unfortunate in my view that the authors chose a 'black box' to call variants. This makes it very difficult to judge the quality of the variant calling. For example, I am not entirely convinced that a reference-free variant calling is superior to variant calling after alignment to a reference sequence as it is done for most GBS applications. I have indicated more specific concerns regarding the genotyping below.

Specific comments:

• Line 35: An important aspect of population genomic analyses is to make sure that most markers are neutral and not under selection. How many of the markers that are 'linked' to genes are in exons, introns, 5' and 3' regulatory regions? How many of them result in non-synonymous sequence changes? This is essential information that needs to be specified here or in the results section. The same is true for the information presented in Table S1. What does 'linked to genes' mean exactly?
• Line 107: 'The percentages of markers located within genes and in close proximity to genes (within 10 kb) was 53%, 65% and 70.2% for the CWR, tetraploid and hexaploid markers.' Table S1 however shows that 39,201 (51%) of the CWR markers, 14,127 (27.8%) of the tetraploid markers, and 28,422 (30.2%) of the hexaploid markers are linked to genes. What is the reason for the different percentages in the text and in table S1? This needs to be aligned. What does '#of content genes' and '#of loci on hexaploid' refer to? Also, the numbers are again different in the 'Data_S2' table, which lists 26,607 genic markers (?) for hexaploid, 33,546 for tetraploid, and 28,289 for CWR.
• Line 113: 'Seventy-seven percent of the markers mapped uniquely for the hexaploids on RefSeq v1.0.' What about the other 23% that did not map uniquely? Were they retained in the subsequent analyses? If yes, how did the authors make sure that the observed polymorphism is caused by a true SNP between two accessions at the same locus and not caused by polymorphisms between homoeologous loci? This is a very important point that needs clarification. The authors should re-run some of the analyses with only the uniquely mapped markers. Does this change the outcome of the analyses (for example the shape of the multidimensional scaling plots)?

• Line 139: It would be very interesting to show a multidimensional scaling plot based on genebank origin (CIMMYT vs. ICARDA). Do accessions from the two genebanks cluster separately? The same should be done for the tetraploid accessions.
• Line 144: What is the difference between a cultivar and an elite breeding line? How is a landrace different from a cultivar? Is this linked to collection date or place? Some additional information on this classification would be helpful for non-specialized readers.
• Line 159: 'and suggests that a large portion of the genetic diversity in the landraces has not been utilized in modern breeding'. I feel that Fig.1a does not fully reflect this. Only when looking at the supplementary video this becomes clear. I think it might be better to capture another image of the video for Fig. 1a. For example, I find the image shown after 9 seconds in the video much more convincing. In addition, the color code in Fig. 1a is not optimal. It is nearly impossible to distinguish cultivars form genetic stocks.
• What are the outliers shown in Fig. 1b? I did not find any description in the text.
• Line 295: 'Interestingly, although breeding programs visibly reduced diversity, a few elite breeding lines appear to maintain a wide range of the diversity found among landrace materials'. This is a contradiction. In my view, Fig 2a. shows that the elite lines cover the whole diversity of the landraces, hence no reduction in diversity in modern breeding?
• Line 563: What was the reason for genotyping the samples at two different laboratories? The authors need to demonstrate that this decision does not bias the analysis, for example that there is no clustering according to the laboratories where the samples were sequenced.
• Line 620-625: The SNP markers are called independently of any reference genome. The authors claim that this is an advantage over other genotyping methods. I agree that this might have been the best approach a few years ago. But given that high-quality reference genomes now exist for diploid, tetraploid and hexaploid wheat I doubt that a reference free SNP calling is still the best strategy. For example, around 23% of the markers from hexaploid wheat mapped to multiple positions in the reference genome. This could indicate that the observed polymorphisms are due to homoeologous loci and not differences between accessions at the same locus. More information on this is needed.
• On a similar note, important information about the sequencing itself is lacking. The authors only mention that the samples were sequenced on an Illumina HiSeq 2500. Was this single or paired-end sequencing? What was the read length? This is important information when it comes to mapping against the various reference genomes.
• Line 964: The authors declare no competing interests. However, one of the authors (Andrzej Kilian) is working for the DArT company and thus has a certain interest in promoting DArT markers. This is legitimate but should be mentioned here in my view.

Reviewer #2 (Remarks to the Author):

Sansaloni et al., Dissecting wheat biodiversity to ensure bread for future generations

Sansaloni et al embark towards the characterisation, analysis and ordering of wheat genebank accessions. The Dart approach used allows to skim almost 80000 (!!!!!!) wheat accessions in the two most important germplasm repositories worldwide in an economic manner. The approach and aim is fairly similar to a recent report on the characterisation and analysis of the barley germplasm (PMID:31253974). (Btw: this should be mentioned and cited; nop I'm not an author of this paper). Breadth of the analysis, the huge collections analysed and structured and the sheer economic, scientific and socioeconomic importance of wheat underpin the high priority of structuring, analysing and exploiting the germplasm collections using powerful NGS and data analysis approaches for very practical and urgent needs we have. A very important and valuable contribution for next generation breeding and structuring/exploitation of our germplasm resources! Nevertheless, I have a couple of points I have to mention and cause me some difficulties in understanding:

Lines 181 ff: Fst values are being calculated in a series of different figures. It (1) wouldn't harm to

introduce the concept and question addressed by the Fst values as well as the meaning/assumption of th thresholds used. (2) It is unusual and without any value to calculate chromosome scale Fst's. Usually Fst's are computed for sliding windows and drops or steep increases demarcate potential selection. Complementary measures are often discussed and used in parallel as well. Since the sequences are based on Dart approach and technology the resulting sequence information doesn't deliver longer continuous sequences. Is it valid to use the short (77bp, correct?) sequence reads for Fst calculations? If so, I'd like to see sliding windows on selected chromosomes that ideally match and are confirmed by previously reported Fst profiles that were based on whole genome profiles (eg.: PMID:3096261). Avoid the Fst values computed for whole chromosomes.
Figures: some of the figures are direct output of the programs used for the respective analysis and are more or less screen shot quality. Can this be amended? In some of the figures one or the other axes is simply missing. Also for all of the "C" figures (bar chart type diversity analysis) This is really enigmatic and hard to grasp/non-intuitive. The figure legends don't give sufficient information, abbreviations used are not explained (I can guess though…) and why in some of the fields numbers are given and in other not is unclear. Btw: what are these numbers? (again I can guess, but…). As already spelled out above: I don't think that given whole genome/-chromosome Fst values is a valid way to make use of the Fst analysis. I'd be grateful for a modification.

Some (minor) comments and criticism:
Line 66: "…linkage drag, resulting from the numerous undesired or deleterious genes …" well the linkage drag is not a consequence of introduction of undesired genes as suggested in this sentence. Also I'd be very sceptical about the concept of introduction of deleterious genes. Less favourable genes or alleles yes, but I'm not sure whether any case of a deleterious gene introduction (in a molecular and mechanistic rather than genetic sense) has been demonstrated. Can you modify sentence and argument?
Line 101: you might ant references to more recent large scale/genomic reports that also report on gene flow and introgressions among different wheat species. PMID:31043759, PMID:31043760
Lines 122-125: The percentage of SNP markers with genetic/genomic positions is fairly low given full genome reference genomes. Why is this? Ambigious mappings because of short sequence length?
Line 134: What are "Fst values on a per marker basis"? Please clearify. What is sequence window chosen?
Line 201: "…likely tetraploids…" can this be tested and confirmed?
Figure 1E: what is cluster 3? And is 3D and 4D really significantly different to some of the other D chromosomes?
In general: can you please report only one digit after the comma (e.g. 2,5 instead of 2,53)? Would improve readability…
Line 311/312: "The third division…" This an enigmatic sentence. Can you amend and translate into less population genetics/genomics terms? The same is true for Figure 2 legend. Second division of three clusters… ????
Line 374: involved rather than involve
Line 318/319: 2E is supposed to show that 1B and 7B provide outstanding Fst values/diversity. I'm not convinced about this argument when checking the plot. Any significance measures?

Reviewer #3 (Remarks to the Author):

An extensive program to genotype a large proportion of the wheat genetic resources held in the CIMMYT and ICARDA genebanks is described in the paper. The report covers over 56,000 accessions contributing around 10% of the total number of samples in the global collections. This represents a

considerable body of work and provides a resource that should be of great value to the wheat research community. The paper is very descriptive since it focuses on the relationship between the different groups of accessions. There are no major surprises with the key conclusion “The analysis revealed landraces with unexplored diversity, presenting fertile ground for exploration and application in breeding programs developing the wheat varieties of the future.” (lines 36-37).
Although, we are repeatedly told how important and powerful the genotyping datasets will be for wheat improvement, there is no real attempt made by the authors to show how this would be achieved. Similarly, there is little analysis of the nature of the different germplasm pools, such as a study of signatures for selection or adaptation, or opportunities for linking genotypic data to agronomic traits. The lack of discussion around application with examples, is a major failing of the current version.

Other points
154 unclassified samples – from the genotyping data can they work out what these are? Does the genotyping data give any clues to their origin?
167-169 Information on synthetics – when and how were they generated? Is there a record of their production? Given the apparent importance of the synthetics in the elite germplasm pool, some analysis on the timing of their generation and rationale for the specific crosses could be interesting and could provide an interesting discussion point, particaulry given the recent publications from CIMMYT indicating the importance of these lines in their current breeding program.
174 The multiple subdivisions of the Mexican lines is confusing. We have 1_Landraces Mexico, 5_Tradicional landraces Mexico, 7_Modern Mexico and 9_Modern landraces Mexico. Given the location of CIMMYT headquarters in Mexico, what is the relationship of these to the CIMMYT program? Can some explanation be provided on this material?
308-311 and later There is the broad question around the purity of the lines used. Presence of hexaploids in tetraploid accessions raises general questions of seed purity and whether the passport data can be trusted. Also from the analysis of replicates (see below) is there an opportunity to assess the over levels of heterozygocity in the lines assayed?
313-318 Since Ethiopian landraces made up over 18% of the accessions – is the high diversity of this material a reflection of abundance of accessions rather than a true representation of diversity?
Figure 2E Cluster 4 the “Outliers” are suspected hexaploids. Since this is essentially a group of lines that have incorrect passport information, it may be best to eliminate them from the analysis.
CWT section The small sample size for many of the species may mean that the diversity reflects population structure rather than true diversity since some species were probably only sampled from a small region. This problem can be seen in the strange distribution for some species which implies there may be both greater diversity within species and other relationships between species, but these have not been captured due to the small and local sampling.
510-514 “Further analysis identified genomic ‘hot spots’ or regions effecting changes between important germplasm groups, thereby suggesting targets for research and breeding efforts; for example, footprints where key genetic changes have been effected by breeding programs as they developed elite lines and cultivars, or genomic regions where synthetics harbor greatest diversity relative to elite breeding germplasm.” It is not clear where this analysis is shown in the results and discussion section.
519-521 “The analysis of the 18,946 tetraploids emphasized the strong bottleneck in diversity introduced by recent breeding programs, but also identified a few elite lines that seemingly break this trend and could be of special value.” Why do they believe these lines are of “special value” and how would they be used? Some discussion of the implications would be helpful. Can they provide an explanation for the elite lines that “break this trend”?
Methods
Plants - Five plants were grown for each accession but only one plant used for the DNA analysis. It appears they didn’t collect seed of the plants used for the DNA extraction. This is unfortunate since this would have made good reference material. What happened to the other four plants – did they check for homogeneity? – From 591-599 they described the technical replication – can they comment on the homogeneity of the plants and purity of the seed stock?

In the methods we are told that the SilicoDArTs “detect methylation variation”. Has this been proven or is it just supposition? If so, what are the implications for the analysis and the stability of the polymorphisms they used for the analysis. We know that there are extensive epigenetic changes associated with polyploidisation, so using these markers may give a distorted view of diversity.
535-541 The final paragraph on the importance of genotyping is of questionable value. This is really just waffle.

Minor issues
26-27 The opening sentence suggests a direct link between climate change, human population growth and the use of genetic resources. This should be rewritten to provide a clear reason for why diversity is so important in crop breeding.
28 What is the difference between “Undomesticated wild species” and "crop wild relatives” with respect to “wheat improvement”?
32 “Presence/absence” to “presence/absence”
41 Wheat is the “most widely-grown crop” not “one of the world’s three most widely grown”
46 “processed for various other uses” add "other"
89 “to elicit initial insights” into what?
219 Figure 1A Although clustered to one side of the plot, there does seem to be a good distribution of “Breeder Elite Line” across the full spectrum of diversity. Can they comment on this?
Fig 2B 5 Tradicional landraces Mexico
274 heterocigocity
Fig 1C Hard to understand and read – maybe look at an alternative labelling of the columns
396 “Ae. Sharonensis” to “Ae. sharonensis”
402 Ae. Biuncialis
494 There have been other extensive genotyping surveys, so this is not really unique as claimed.
496 Why do they claim DArTSeq is “uniquely suited”. Several publications suggest that other techniques are superior.
521-523 Twice “finally”
527-528 “This finding is of great use to genebank managers, who are validating prior to correcting any erroneous passport data.” What does this mean?

Methods
577-578 and 607 The use of proprietal software is always a bit problematic. Can they provide a simple overview of the software to help the reader understand what was actually done with the data.
700-701 “We used the base-2 logarithm as when the allele frequencies are equal to 0.5 the index value is 1.0, maximum of diversity.” What does this mean?

Reviewers' comments:

Reviewer #1 (Remarks to the Author):

In this manuscript, Sansaloni and colleagues present the results of a large-scale genotyping effort on wheat accessions from the CIMMYT and ICARDA germplasm collections. The biggest novelty of the study is the sheer scale of the data set. In total, the authors genotyped close to 80,000 wheat accessions and I am not aware of a larger genotyping effort for a germplasm collection. This effort clearly shows that the transition to digital germplasm collections is now feasible. As such, the topic and scale of the analyses presented here are timely and highly relevant. I particularly liked the visualization of the data in CurlyWhirly, which is innovative and cool. The results section however does not go beyond a basic population genomics description of the data set. Unfortunately, there is no information in the results on how this genomic information can now be utilized for breeding. Given the 'big promises' made in the title this was slightly disappointing. For example, is there
historic phenotypic information for some of the accessions that could be utilized for a GWAS? Such an additional analysis would be highly valuable to demonstrate that this data set is useful to dissect traits and to transfer beneficial alleles from landraces into elite material by using the markers described in this study. Also, the results section is very repetitive, since the same analyses are essentially presented three times for hexaploid, tetraploid, and wild wheats, respectively.

Another important aspect is accessibility of data for breeders. Unfortunately, the two links provided in the 'data and materials availability' section are not yet active. For example, is it possible to retrieve the accession names from the multidimensional scaling plots in CurlyWhirly by simply clicking on one of the dots?

In the data statement that we sent, we specified that the data would be available in those public resources at the time of publication. But, for Dataverse we did provide a special username and password.
For Dataverse the account is:
Username: NC_reviewer_2019
Password: NC_reviewer_2019

A last major concern is the choice of genotyping method, or more precisely, the use of a proprietary software for variant calling. There are numerous open source tools available to handle this kind of sequencing data and it is very unfortunate in my view that the authors chose a 'black box' to call variants. This makes it very difficult to judge the quality of the variant calling. For example, I am not entirely convinced that a reference-free variant calling is superior to variant calling after alignment to a reference sequence as it is done for most GBS applications. I have indicated more specific concerns regarding the genotyping below.

DArTseq has became a technology of choice for practically all areas of research involving over 1,000 species of plants, animals and microbs. There are hundreds of papers published using this technology package taking advantage of its cost and time effectiveness. It is mostly due to the

full integration of library construction methods with analytical procedures implemented in DArtsoft14 algorithms. More information on the actual algorithms involved in this software was added to the M&M section

Specific comments:

• Line 35: An important aspect of population genomic analyses is to make sure that most markers are neutral and not under selection. How many of the markers that are 'linked' to genes are in exons, introns, 5' and 3' regulatory regions? How many of them result in non-synonymous sequence changes? This is essential information that needs to be specified here or in the results section. The same is true for the information presented in Table S1. What does 'linked to genes' mean exactly?

Please see Data S2- Gene Annotation, Column E.

• Line 107: 'The percentages of markers located within genes and in close proximity to genes (within 10 kb) was 53%, 65% and 70.2% for the CWR, tetraploid and hexaploid markers.' Table S1 however shows that 39,201 (51%) of the CWR markers, 14,127 (27.8%) of the tetraploid markers, and 28,422 (30.2%) of the hexaploid markers are linked to genes. What is the reason for the different percentages in the text and in table S1? This needs to be aligned. What does '#of content genes' and '#of loci on hexaploid' refer to? Also, the numbers are again different in the 'Data_S2' table, which lists 26,607 genic markers (?) for hexaploid, 33,546 for tetraploid, and 28,289 for CWR.

These discrepancies were fixed in the table and text.

• Line 113: 'Seventy-seven percent of the markers mapped uniquely for the hexaploids on RefSeq v1.0.' What about the other 23% that did not map uniquely? Were they retained in the subsequent analyses? If yes, how did the authors make sure that the observed polymorphism is caused by a true SNP between two accessions at the same locus and not caused by polymorphisms between homoeologous loci? This is a very important point that needs clarification. The authors should re-run some of the analyses with only the uniquely mapped markers. Does this change the outcome of the analyses (for example the shape of the multidimensional scaling plots)?

For the analysis, we used only the uniquely mapped markers in the hexaploid and tetraploid group. This was not clear in the manuscript, but we added one sentence clarifying this. For the CWR group we consider that was appropriate to use marker mapped uniquely and marker not mapped since the group include a wide range of exotic accession with different genomes.

• Line 139: It would be very interesting to show a multidimensional scaling plot based on genebank origin (CIMMYT vs. ICARDA). Do accessions from the two genebanks cluster separately? The same should be done for the tetraploid accessions.

In the hexaploid we can not clearly differentiate clusters based on genebank origin.

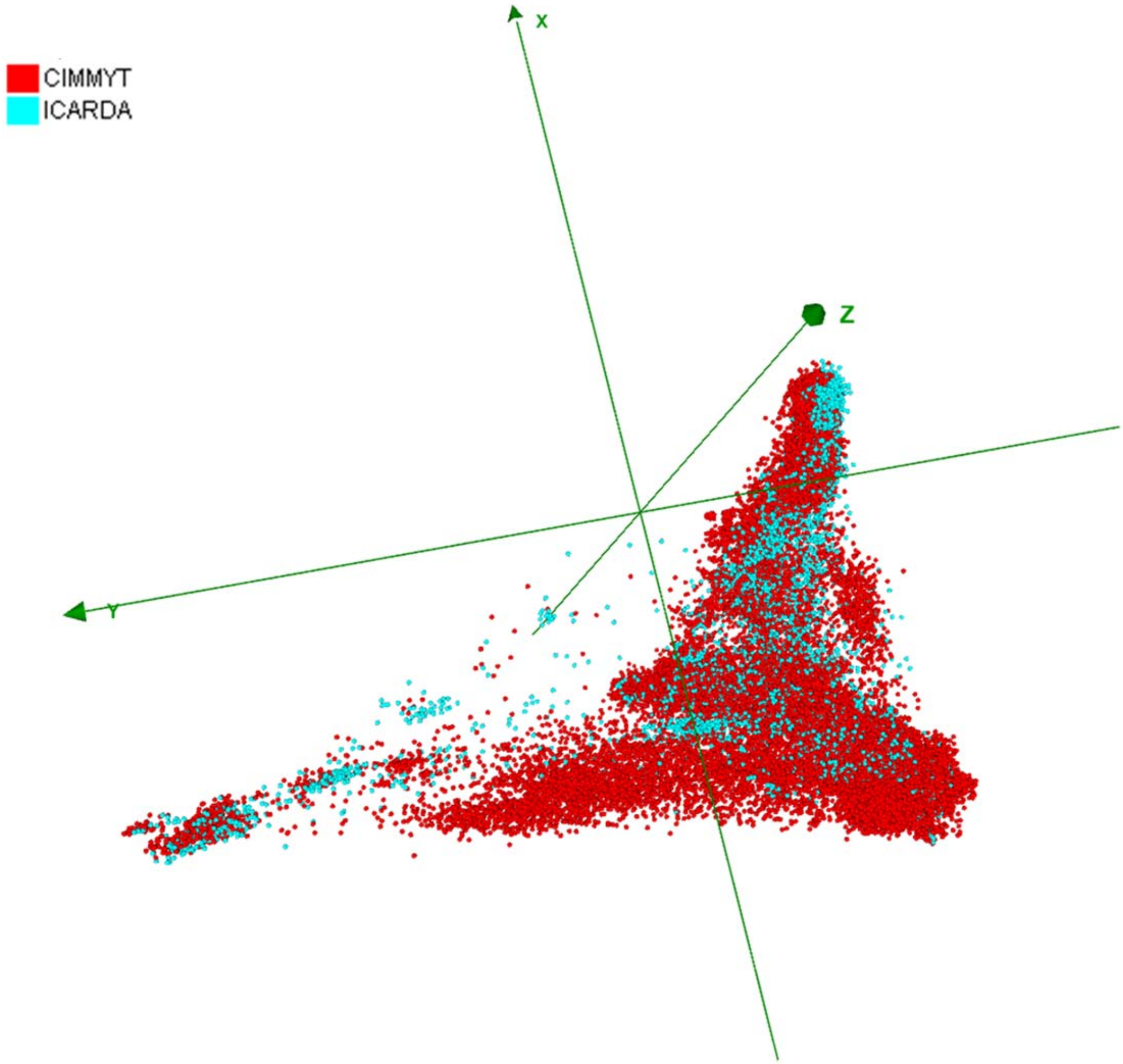

In the Tetraploid group we can observed that the elite material are mostly belong to CIMMYT genebank and the landraces from ICARDA.

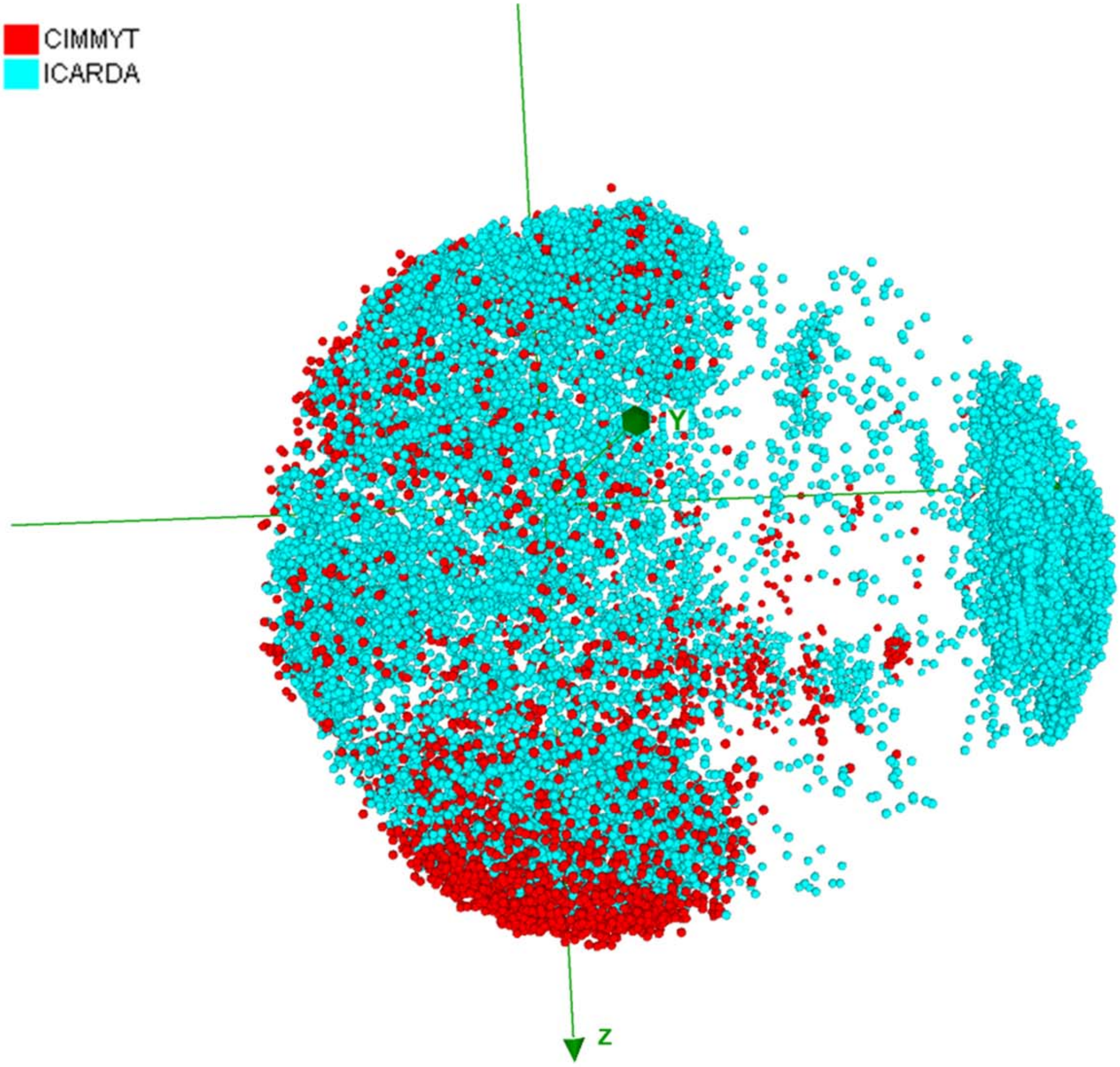

• Line 144: What is the difference between a cultivar and an elite breeding line? How is a landrace different from a cultivar? Is this linked to collection date or place? Some additional information on this classification would be helpful for non-specialized readers.

Typically, a wheat landrace is a population or mixture of pure lines, while a variety or cultivar is a genetically and phenotypically “distinct, uniform and stable”. A landrace that consists of

multiple pure lines can be “de-bulked” resulting in a set of individual pure lines, which collectively represent the original mixed landrace. This approach, used for the Mexican and Iranian wheat landraces held by CIMMYT, permits the maintenance of rare alleles, and the characterization, evaluation and genotyping of pure-lines.

• Line 159: ‘and suggests that a large portion of the genetic diversity in the landraces has not been utilized in modern breeding’. I feel that Fig.1a does not fully reflect this. Only when looking at the supplementary video this becomes clear. I think it might be better to capture another image of the video for Fig. 1a. For example, I find the image shown after 9 seconds in the video much more convincing. In addition, the color code in Fig. 1a is not optimal. It is nearly impossible to distinguish cultivars form genetic stocks.

This is the fig 1a after 9 sec of the video. We also changed the color code.

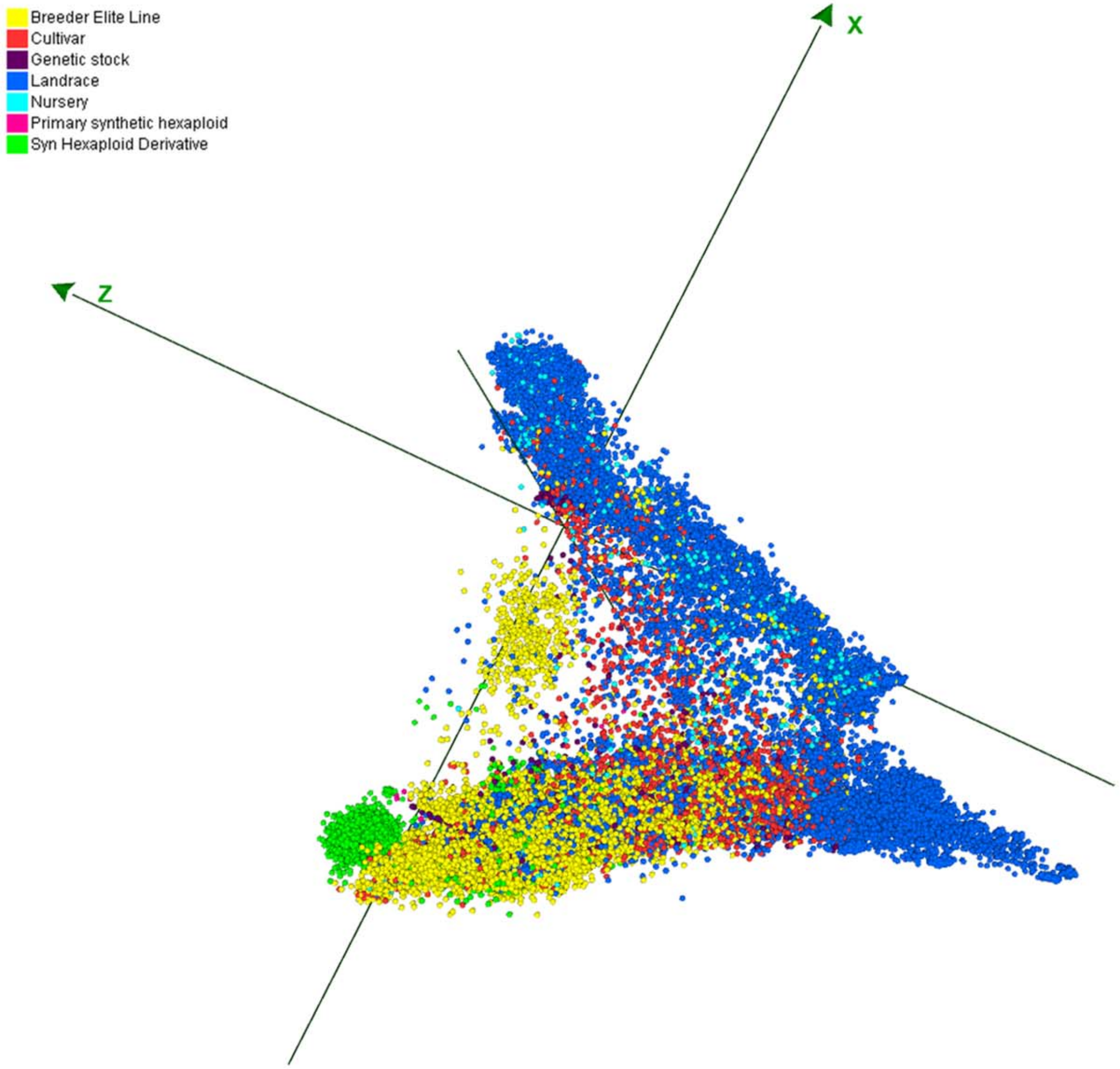

• What are the outliers shown in Fig. 1b? I did not find any description in the text.

From the text: Two small sub-groups (2, 8), comprising 2.6% of the accessions, were separated from the Syn A group (10); based on their lack of markers on chromosome D, these two small sub-groups are likely tetraploids that were originally miss-classified as hexaploids in their passport data and were identify in the analysis as Outliers.

• Line 563: What was the reason for genotyping the samples at two different laboratories? The authors need to demonstrate that this decision does not bias the analysis, for example that there is no clustering according to the laboratories where the samples were sequenced.

At the beginning of the project, we started the genotyping at DArT Company while we built the genotyping laboratory at CIMMYT. Then we continue we the generation of data at cIMMYT genotyping platform using the same technology. We cross validate several time the data generated in both lab always giving 99.9% of reproducibility.

• Line 620-625: The SNP markers are called independently of any reference genome. The authors claim that this is an advantage over other genotyping methods. I agree that this might have been the best approach a few years ago. But given that high-quality reference genomes now exist for diploid, tetraploid and hexaploid wheat I doubt that a reference free SNP calling is still the best strategy. For example, around 23% of the markers from hexaploid wheat mapped to multiple positions in the reference genome. This could indicate that the observed polymorphisms are due to homoeologous loci and not differences between accessions at the same locus. More information on this is needed.

The choice of DArTsoft14 over alternative methods was based on two foundations: 1. this software was developed specifically to work on the sequence libraries generated by DArTseq method and exploits effectively characteristics of these libraries. The fixed fragment length enables very fast clustering algorithm and methyl filtration step reduces dramatically the presence of repetitive sequences in the libraries. The software is also fully integrated with DArTdb database/LIMS system therefore securing complete data integrity. The software uses highly compressed input file format (fastqcol) reducing data storage cost and enabling very fast processing of even very large data set. The analysis of the size reported here can be completed well within 24 hours on modest power computers. 2. While reference sequence data for some wheat accessions became recently available, their quality and relevance for the materials analysed in the project is still somewhat limited. Unfortunately having reference sequence in hand does not reduce in any significant way the risk of ploidy affecting marker calling process in some way. When mapping the short reads (69 bp) onto very large, hexaploid reference genome with high level of genetic redundancy and with already known high level of structural diversity does not offer complete assurance that the correct homeolog is matched with the tag. In fact the use of DArT consensus map generated with the same genome complexity reduction as diversity analysis reported here already applied offers significant increase in unique and precise mapping of the reads to a unique genome position.

• On a similar note, important information about the sequencing itself is lacking. The authors only mention that the samples were sequenced on an Illumina HiSeq 2500. Was this single or paired-end sequencing? What was the read length? This is important information when it comes to mapping against the various reference genomes.

From Method section: After PCR, equimolar amounts of amplification products from each sample of the 96-well microtiter plate were bulked, purified, quantified and amplified (c-Bot

bridge amplification, Illumina Inc., San Diego, CA), followed by single read sequencing of 77 cycles on Illumina Hiseq 2500 (Illumina Inc., San Diego, CA).

• Line 964: The authors declare no competing interests. However, one of the authors (Andrzej Kilian) is working for the DArT company and thus has a certain interest in promoting DArT markers. This is legitimate but should be mentioned here in my view.

Competing interest. The authors declare no competing interests, except Andrzej Kilian and Jason Carling who works at DArT Company.

Reviewer #2 (Remarks to the Author):

Sansaloni et al., Dissecting wheat biodiversity to ensure bread for future generations

Sansaloni et al embark towards the characterisation, analysis and ordering of wheat genebank accessions. The Dart approach used allows to skim almost 80000 (!!!!!!) wheat accessions in the two most important germplasm repositories worldwide in an economic manner. The approach and aim is fairly similar to a recent report on the characterisation and analysis of the barley germplasm (PMID:31253974). (Btw: this should be mentioned and cited; nop I'm not an author of this paper). Breadth of the analysis, the huge collections analysed and structured and the sheer economic, scientific and socioeconomic importance of wheat underpin the high priority of structuring, analysing and exploiting the germplasm collections using powerful NGS and data analysis approaches for very practical and urgent needs we have. A very important and valuable contribution for next generation breeding and structuring/exploitation of our germplasm resources! Nevertheless, I have a couple of points I have to
mention and cause me some difficulties in understanding:

Lines 181 ff: Fst values are being calculated in a series of different figures. It (1) wouldn't harm to introduce the concept and question addressed by the Fst values as well as the meaning/assumption of th thresholds used. (2) It is unusual and without any value to calculate chromosome scale Fst's. Usually Fst's are computed for sliding windows and drops or steep increases demarcate potential selection. Complementary measures are often discussed and used in parallel as well. Since the sequences are based on Dart approach and technology the resulting sequence information doesn't deliver longer continuous sequences. Is it valid to use the short (77bp, correct?) sequence reads for Fst calculations? If so, I'd like to see sliding windows on selected chromosomes that ideally match and are confirmed by previously reported Fst profiles that were based on whole genome profiles (eg.: PMID:3096261). Avoid the Fst values computed for whole chromosomes.

From the text: Calculation of the fixation index (Fst) on a per marker basis and the average value on a 1Mb window identified regions in the genome where the population structure explains

a high proportion of the genetic diversity. This analysis suggest there are different levels of allelic fixation between the sub-groups defined above.

Figures: some of the figures are direct output of the programs used for the respective analysis and are more or less screen shot quality. Can this be amended? In some of the figures one or the other axes is simply missing. Also for all of the "C" figures (bar chart type diversity analysis) This is really enigmatic and hard to grasp/non-intuitive. The figure legends don't give sufficient information, abbreviations used are not explained (I can guess though…) and why in some of the fields numbers are given and in other not is unclear

We only show the number in the 2 group that are part of the division, not in the other because will be redundant.

Btw: what are these numbers?

The number inside the boxes are the He (again I can guess, but…).

C) Representation of the distribution of 12 groups based on clusters analysis. The size of the boxes are proportional to the number of accession. In the right side are the Fixation index (Fst) values and inside the boxes the expected heterocigocity (He) of each group division and in the bottom the 12 clusters are identified with a brief description and the number which corresponds to figure 1B. Left numbers are the number of levels.

As already spelled out above: I don't think that given whole genome/-chromosome Fst values is a valid way to make use of the Fst analysis. I'd be grateful for a modification.

Some (minor) comments and criticism:
Line 66: "…linkage drag, resulting from the numerous undesired or deleterious genes …" well the linkage drag is not a consequence of introduction of undesired genes as suggested in this sentence. Also I'd be very sceptical about the concept of introduction of deleterious genes. Less favourable genes or alleles yes, but I'm not sure whether any case of a deleterious gene introduction (in a molecular and mechanistic rather than genetic sense) has been demonstrated. Can you modify sentence and argument?

Several challenges limit breeders' use of germplasm bank accessions, but the biggest hurdles are 1) identifying which of the possible 560,000 accessions to use, and 2) the co-introduction of less favorable alleles when landraces and CWR are crossed with elite lines.

Line 101: you might ant references to more recent large scale/genomic reports that also report on gene flow and introgressions among different wheat species. PMID:31043759, PMID:31043760

Lines 122-125: The percentage of SNP markers with genetic/genomic positions is fairly low given full genome reference genomes. Why is this? Ambigious mappings because of short sequence length?

Line 134: What are “Fst values on a per marker basis”? Please clearify. What is sequence window chosen?

From the text: Calculation of the fixation index (Fst) on a per marker basis and the average value on a 1Mb window identified regions in the genome where the population structure explains a high proportion of the genetic diversity. This analysis suggest there are different levels of allelic fixation between the sub-groups defined above.

Line 201: “…likely tetraploids…” can this be tested and confirmed?

Most of accessions identified as outliers were visually tested and we confirmed that were tetraploid (Durum wheat). Some of the one that we still have doubt we will plant in the field for further evaluation.

Figure 1E: what is cluster 3? And is 3D and 4D really significantly different to some of the other D chromosomes?

Cluster 3 is the level in which the clustering analysis is divided in 3 groups. The synthetic wheat group (purple) is differentiated from the Elite/landrace group (red). It is expected that the D genome will be significantly different between the groups since the synthetic are created from a tetraploid wheat with an exotic D genome (Aegilop taichii).

Part of figure 1C:

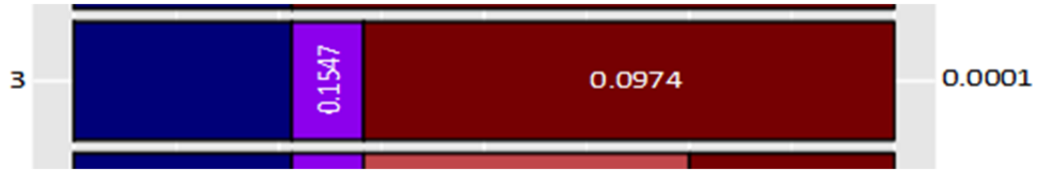

In general: can you please report only one digit after the comma (e.g. 2,5 instead of 2,53)? Would improve readability…

Modified in the text.

Line 311/312: “The third division…” This an enigmatic sentence. Can you amend and translate into less population genetics/genomics terms? The same is true for Figure 2 legend. Second division of three clusters… ????

Modified in the text.

Line 374: involved rather than involve

Modified in the text.

Line 318/319: 2E is supposed to show that 1B and 7B provide outstanding Fst values/diversity. I'm not convinced about this argument when checking the plot. Any significance measures?

Modified in the text.

Reviewer #3 (Remarks to the Author):

An extensive program to genotype a large proportion of the wheat genetic resources held in the CIMMYT and ICARDA genebanks is described in the paper. The report covers over 56,000 accessions contributing around 10% of the total number of samples in the global collections. This represents a considerable body of work and provides a resource that should be of great value to the wheat research community. The paper is very descriptive since it focuses on the relationship between the different groups of accessions. There are no major surprises with the key conclusion "The analysis revealed landraces with unexplored diversity, presenting fertile ground for exploration and application in breeding programs developing the wheat varieties of the future." (lines 36-37).
Although, we are repeatedly told how important and powerful the genotyping datasets will be for wheat improvement, there is no real attempt made by the authors to show how this would be achieved. Similarly, there is little analysis of the nature of the different germplasm pools, such as a study of signatures for selection or adaptation, or opportunities for linking genotypic data to agronomic traits. The lack of discussion around application with examples, is a major failing of the current version.

Other points
154 unclassified samples – from the genotyping data can they work out what these are? Does the genotyping data give any clues to their origin?

We tried to classify using genotyping data, but we need to test and confirm the results and will be for further analysis.

167-169 Information on synthetics – when and how were they generated? Is there a record of their production? Given the apparent importance of the synthetics in the elite germplasm pool, some analysis on the timing of their generation and rationale for the specific crosses could be interesting and could provide an interesting discussion point, particaulry given the recent publications from CIMMYT indicating the importance of these lines in their current breeding program.

A recent study (Rosyara et al. 2019) highlighted the contributions of synthetic wheats in maintaining and enhancing both genetic diversity and genetic gains over the years. Since 1986, CIMMYT has generated more than 1,400 synthetic wheats (spring type), and crosses were then made between the most promising ones and elite bread wheat lines. According to our database,

the initial crosses for the lines used in this study were made between 1986 and 2012, with the majority of crosses concentrated in 2009 and 2012. The distribution of the number of crosses per year is similar between Syn A and Syn B groups. The resulting introgressions of D' genome to synthetic derivative lines have contributed with novel variation for particular traits of interest. Currently, approximately 50 targeted synthetics are developed in CIMMYT annually, by crossing elite durum wheats with *Ae. tauschii* accessions selected based on their genetic diversity.

Umesh Rosyara, Masahiro Kishii, Thomas Payne, Carolina Paola Sansaloni, Ravi Prakash Singh, Hans-Joachim Braun & Susanne Dreisigacker. Genetic contribution of synthetic hexaploid wheat to CIMMYT's spring bread wheat breeding germplasm. Scientific Reports, volume 9, Article number: 12355 (2019) https://www.nature.com/articles/s41598-019-47936-5

174 The multiple subdivisions of the Mexican lines is confusing. We have

1_Landraces Mexico,

5_Tradicional landraces Mexico,

7_Modern Mexico and

9_Modern landraces Mexico.

Given the location of CIMMYT headquarters in Mexico, what is the relationship of these to the CIMMYT program? Can some explanation be provided on this material?

Wheat was introduced to Mexico from the Mediterranean area (Spain) ~500 years ago. In collaboration with CONABIO (the Mexican National Commission for the Study and Use of Biodiversity), CIMMYT collected landraces from more than 300 locations in 16 states (Skovmand et al., 1995; project conducted during 1994-1998). These landraces (most of which do not exist anymore in Spain) are still grown in some areas of Mexico because of their special adaptation to stresses.

Being CIMMYT based in Mexico and actively promoting exchange of genetic resources with national programme partners, the coexistence of landraces with higher yielding semi-dwarf lines is expected. Some traditional landraces may consequently have introgressions from modern cultivars, resulting in the sub-group named 'modern landraces' ('introgressed landraces' according to Casañas et al., 2017). Recent studies have proposed to open the concept of landraces, to incorporate these variations resulting from population dynamics and constant state of evolution resulting from natural and artificial selection (Villa et al. 2005; Casañas et al. 2017).

We agree that the name of sub-group 9 is confusing. The group is too small to allow interpretation of its uniqueness, and it is now referred as Modern landraces 1 and Modern landraces 2

Skovmand, B., P. N. Fox, G. Varughese, D. -de-LeoGonzalez 1995. International Activities in Wheat Germplasm: CIMMYT's Perspective. In: R. R. Duncan, editor, International Germplasm Transfer: Past and Present, CSSA Spec. Publ. 23. CSSA and ASA, Madison, WI. p. 135-148. doi:10.2135/cssaspecpub23.c10.

- Casañas, F., Simó, J., Casals, J., & Prohens, J. (2017). Toward an Evolved Concept of Landrace. Frontiers in plant science, 8, 145. doi:10.3389/fpls.2017.00145
- Villa, T., Maxted, N., Scholten, M., & Ford-Lloyd, B. (2005). Defining and identifying crop landraces. Plant Genetic Resources, 3(3), 373-384. doi:10.1079/PGR200591
- http://www.conabio.gob.mx/institucion/cgi-bin/datos.cgi?Letras=E&Numero=1

308-311 and later There is the broad question around the purity of the lines used. Presence of hexaploids in tetraploid accessions raises general questions of seed purity and whether the passport data can be trusted. Also from the analysis of replicates (see below) is there an opportunity to assess the over levels of heterozygocity in the lines assayed?

The problem of purity, that is, homogeneity/heterogeneity between plants from the same accession is under study. Below you can see the “unpublished” (in process) results of a job conducted using 70 accessions and 20 plants per accession, and the modified Rogers’ distance (mrd). 90% of distances between accessions showed a value less than or equal to 0.64 while for the “between plants within accession” that percentile was 0.07, and for the between “technical repetitions the 90% percentile was 0.05. As the accessions were selected at random we would think there is a small representative sample from the collection.

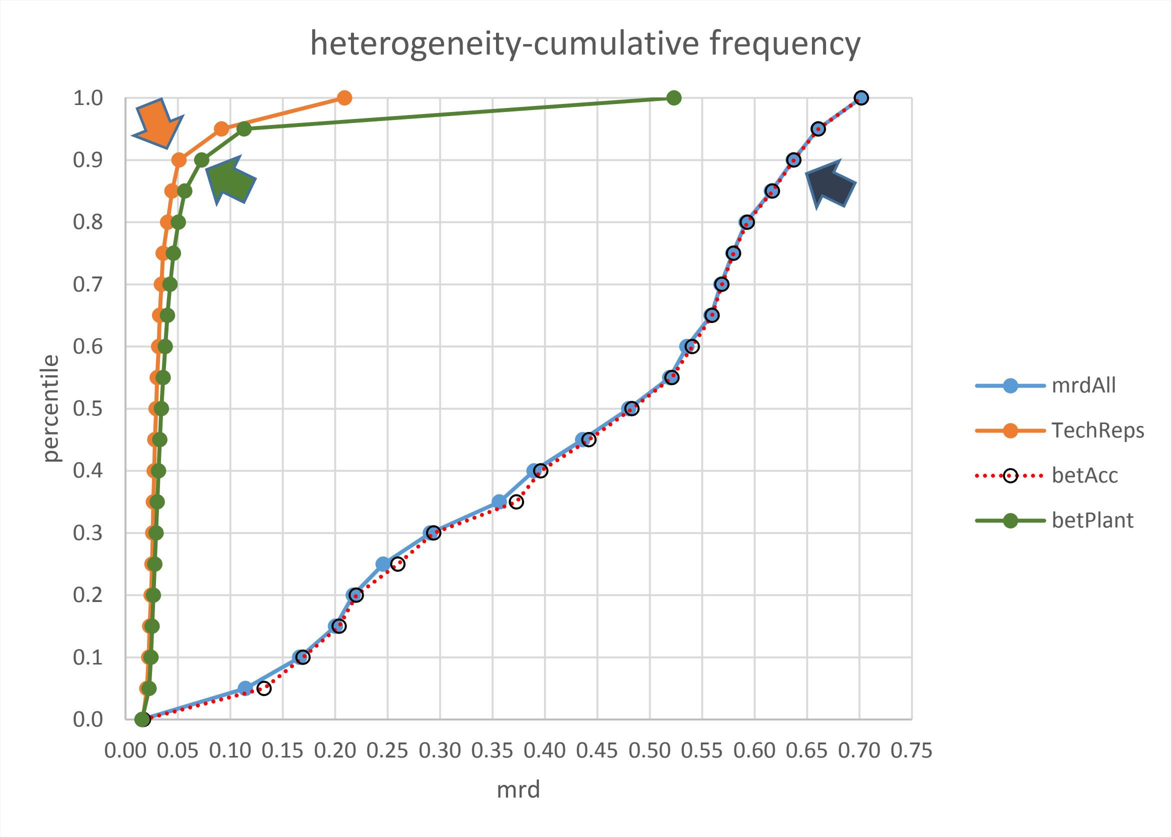

313-318 Since Ethiopian landraces made up over 18% of the accessions – is the high diversity of this material a reflection of abundance of accessions rather than a true representation of diversity?

Tetraploid wheat has been cultivated in Ethiopia for thousands of years, and the area is considered center of diversity for that species (Harlan, 1971). Therefore, it is expected that the analyses reflect the high diversity observed in the region. The big number of accessions kept in working collections may also relate to the high diversity observed in the material.

Harlan JR. Agricultural origins: centers and noncenters. Science. 1971 Oct 29;174(4008):468-74

Figure 2E Cluster 4 the "Outliers" are suspected hexaploids. Since this is essentially a group of lines that have incorrect passport information, it may be best to eliminate them from the analysis.

We kept these outliers in the analysis since it is a very important outcome from this study for Genebank management porpuse. They can now fix the passport information.

CWT section The small sample size for many of the species may mean that the diversity reflects

population structure rather than true diversity since some species were probably only sampled from a small region. This problem can be seen in the strange distribution for some species which implies there may be both greater diversity within species and other relationships between species, but these have not been captured due to the small and local sampling.

510-514 “Further analysis identified genomic ‘hot spots’ or regions effecting changes between important germplasm groups, thereby suggesting targets for research and breeding efforts; for example, footprints where key genetic changes have been effected by breeding programs as they developed elite lines and cultivars, or genomic regions where synthetics harbor greatest diversity relative to elite breeding germplasm.” It is not clear where this analysis is shown in the results and discussion section.

More analysis in new version

519-521 “The analysis of the 18,946 tetraploids emphasized the strong bottleneck in diversity introduced by recent breeding programs, but also identified a few elite lines that seemingly break this trend and could be of special value.” Why do they believe these lines are of “special value” and how would they be used? Some discussion of the implications would be helpful. Can they provide an explanation for the elite lines that “break this trend”?

While comparing landraces to elite lines, the analyses demonstrate that crop domestication and breeding selection have reduced the genetic diversity in specific regions in favor of agronomically advantageous alleles (positive selection). This reduction in genetic diversity, however, can increase genetic vulnerability and reduce crop plasticity for adaptation to changes in production environments. Therefore, lines that kept higher frequencies of rare alleles can be used as sources of the putatively lost variability and may provide favorable genes or alleles (Lopes et al. 2015 https://academic.oup.com/jxb/article/66/12/3477/525347). The use of these sources for widening genetic diversity in elite wheat lines requires several actions, including hybridization strategies in breeding programs, proper monitoring of genetic diversity, identification of allelic variations for known functional genes, and promotion of precise phenotypic characterization (Lopes et al. 2015).

Methods

Plants - Five plants were grown for each accession but only one plant used for the DNA analysis. It appears they didn’t collect seed of the plants used for the DNA extraction. This is unfortunate since this would have made good reference material. What happened to the other four plants –

At the beginning of the project it was evaluated to possibility to keep the plants as reference material, but giving the large number of samples it would be impossible or very expensive to maintain the large collection. The plant were destroyed but we keep DNA samples.

did they check for homogeneity?

The problem of purity, that is, homogeneity/heterogeneity between plants from the same accession is under study. Below you can see the “unpublished” (in process) results of a job conducted using 70 accessions and 20 plants per accession, and the modified Rogers’ distance (mrd). 90% of distances between accessions showed a value less than or equal to 0.64 while for the “between plants within accession” that percentile was 0.07, and for the between “technical repetitions the 90% percentile was 0.05. As the accessions were selected at random we would think there is a small representative sample from the collection.

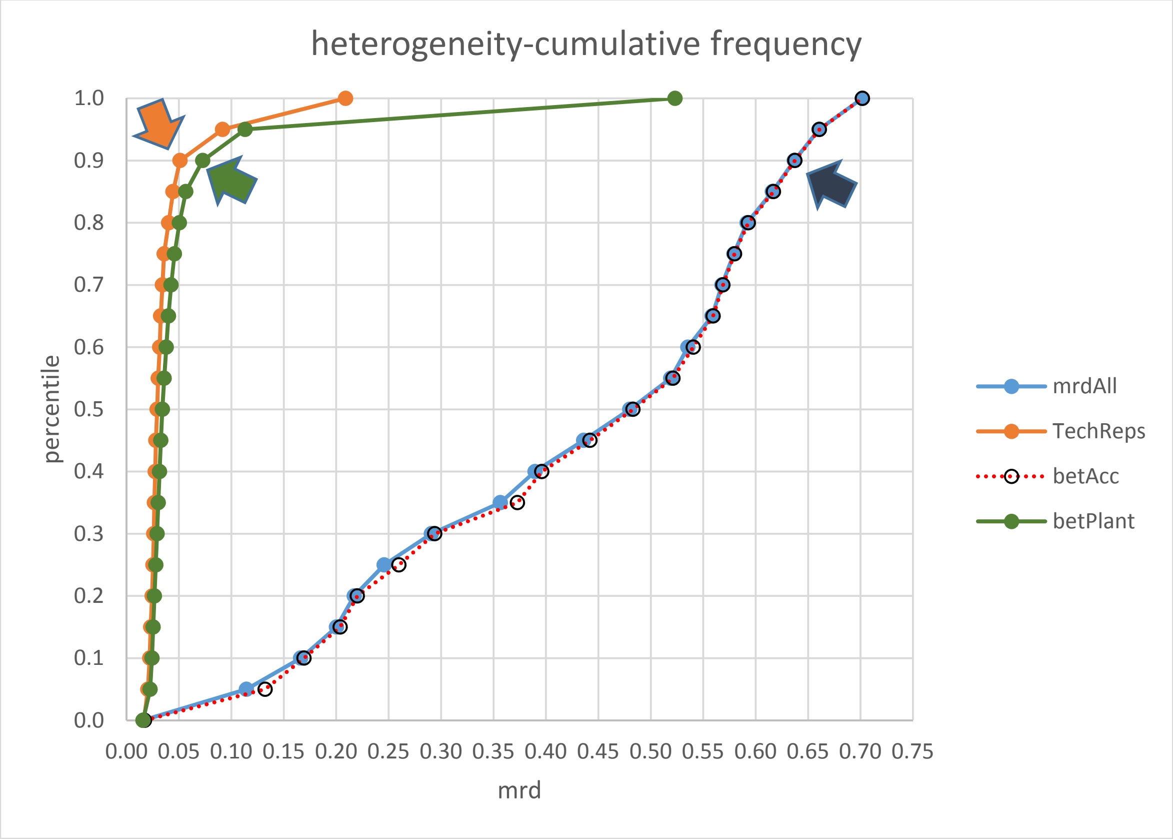

– From 591-599 they described the technical replication – can they comment on the homogeneity of the plants and purity of the seed stock?

See answer above

In the methods we are told that the SilicoDArTs “detect methylation variation”. Has this been proven or is it just supposition? If so, what are the implications for the analysis and the stability of the polymorphisms they used for the analysis. We know that there are extensive epigenetic

changes associated with polyploidisation, so using these markers may give a distorted view of diversity.

We provided in the new submission a reference to the paper in which a small proportion (under 10%) of markers generated with DArT complexity reduction method was attributed to methylation variation. While this proportion may be higher in wheat genome and we do not have exact estimate we are quite confident that the markers based on this type of molecular variation are not distorting the pattern of diversity given well established correlation between sequence divergence and methylation divergence. Importantly, there is generally high level of consensus between the diversity patterns generated by SNPs and SilicoDArTs confirming that methylation variation does not distort the picture in any significant way.

535-541 The final paragraph on the importance of genotyping is of questionable value. This is really just waffle.

Minor issues
26-27 The opening sentence suggests a direct link between climate change, human population growth and the use of genetic resources. This should be rewritten to provide a clear reason for why diversity is so important in crop breeding.

Edited

28 What is the difference between "Undomesticated wild species" and "crop wild relatives" with respect to "wheat improvement"?

32 "Presence/absence" to "presence/absence"

41 Wheat is the "most widely-grown crop" not "one of the world's three most widely grown"
46 "processed for various other uses" add "other"
89 "to elicit initial insights" into what?

Fig 2B 5 Tradicional landraces Mexico
274 heterocigocity
Fig 1C Hard to understand and read – maybe look at an alternative labelling of the columns
396 "Ae. Sharonensis" to "Ae. sharonensis"
402 Ae. Biuncialis
494 There have been other extensive genotyping surveys, so this is not really unique as claimed.
496 Why do they claim DArTSeq is "uniquely suited". Several publications suggest that other techniques are superior.

521-523 Twice “finally”
527-528 “This finding is of great use to genebank managers, who are validating prior to correcting any erroneous passport data.” What does this mean?

They are evaluating the material identified as outlier to confirm they have erroneous passport information. Then, they will modify the passport data into the database.

Methods
577-578 and 607 The use of proprietal software is always a bit problematic. Can they provide a simple overview of the software to help the reader understand what was actually done with the data.
700-701 “We used the base-2 logarithm as when the allele frequencies are equal to 0.5 the index value is 1.0, maximum of diversity.” What does this mean?

Due to the equation, and the fact we are working with a biallelic marker the maximum expected heterozygosity for a marker showing allele frequencies (0.5, 0.5) is he = 1-(0.5^2+(1-0.5)^2) = 0.5, that allele frequencies, when using the base 2 logarithm just produce a Shannon index of: sh = - [(0.5 * log2(0.5) + (0.5 * log2(0.5)] = 1.0

Reviewer #1 (Remarks to the Author):

In their revised version, Sansaloni et al. addressed many of my comments. It is a bit disappointing though that the authors ignored two of my major queries outlined at the beginning of my report: (i) an example of how such a massive data set can be used to assist breeding and (ii) the repetitiveness of the results section (or at least I did not find any information in the response letter and the main text if/how these queries have been addressed. It is a bit unfortunate that no document with track-changes has been added to the revised version). Two very similar comments have also been raised by reviewer 3.

I leave it up to the editor to decide to what extend these two points should be addressed. All the 'technical' comments have been addressed.

As mentioned in my first report, this is to my knowledge the largest genotyping effort in plant science, which justifies publication of this article in Nature Communications.

Reviewer #2 (Remarks to the Author):

Some of my comments haven't been adressed and for some others the response is somewhat cryptic and I'm not sure whether the authors agree and have amended the ms. or not. Can you please clarify these? Also it would be helpful to have a ms. version that highlights the changes undertaken.

Reviewer #3 (Remarks to the Author):

The authors have addressed most of the concerns and questions identified in the reviewers. However, both I and another reviewer have raised the issue of the relevance of this work to wheat breeding programs and suggest the authors provide some examples of how these datasets could be used. The authors have provided the examples of the pre-harvest sprouting gene, and the traits grain protein content and SDS-sedimentation. In addition, they provide several examples of how genotypic data on diversity panels have been used (lines 576 to 591) but these are all examples of previous studies using other datasets. If anything, these examples suggest the large dataset generated here is not required for these types of analyses. The authors should explain why this dataset adds value over and above that provided by smaller studies. Also, given that the title, abstract and introduction all make a big point of the utility of the data in breeding, the relevance to breeding requires further development. The authors have also not effectively dealt with the concerns raised by two reviewers around the use of DArTseq as the genotyping platform. The comment that "DArTseq has become a technology of choice for practically all areas of research…." is not correct and does not address the concerns raised. I don't have a problem with the use of this technique, but the authors cannot ignore the intrinsic limitations of this approach compared to other techniques. Somewhere in the manuscript they need to provide comment and an explanation for why this platform was used.
In response to several of the reviewer questions, the authors have provided detailed replies. They should consider providing some of this additional information in the supplementary files.

REVIEWER COMMENTS

Reviewer #1 (Remarks to the Author):

In their revised version, Sansaloni et al. addressed many of my comments. It is a bit disappointing though that the authors ignored two of my major queries outlined at the beginning of my report: (i) an example of how such a massive data set can be used to assist breeding and (ii) the repetitiveness of the results section (or at least I did not find any information in the response letter and the main text if/how these queries have been addressed. It is a bit unfortunate that no document with track-changes has been added to the revised version). Two very similar comments have also been raised by reviewer 3.

I apology for the lack of track changes in the revised manuscript. The reason is because the manuscript was extensively re-written incorporating all your good suggestion. In the new version I tracked the changes.

(i) **In the line 475 we added novel analysis to the manuscript that show the application of the data in breeding:**

Diversity patterns reveal genomic regions under positive selection

Analysis of Fst values on a variant-per-variant basis across the bread wheat genome highlights areas of positive selection. This is particularly informative when relatively high Fst values are considered together with the backgrounds of the groups defined by the clusters (Data S5). For each cluster split, the highest Fst values reveal the genomic variants that contributed to the separation of the two sub-groups, thereby identifying molecular footprints possibly associated with selective sweeps. We implemented this analysis across the full dataset, noting the genomic regions with high Fst values (Data S9). We illustrate the numerous potentially interesting analyses by focusing on two important cluster splits: (1) the first split, which separates the accessions of traditional germplasm from the group that includes most of the elite lines, and (2) the third split, which consolidates the core cluster of elite lines by removing a large set of Mexican landraces (Fig. S7). This analysis identified genomic regions that are known to be associated with key agronomic traits, but more importantly, we also uncovered many regions that could help explain the recent history of modern wheat breeding and offer target alleles for future breeding. For example, the significant QTL within the region of chromosome 3A associated with the well-characterized pre-harvest sprouting gene (*TaMFT*)[45], are present in germplasm in cluster 2 (elite lines and Mexican landraces) but are absent in cluster 4 (elite lines and cultivars) (Fig. 4).

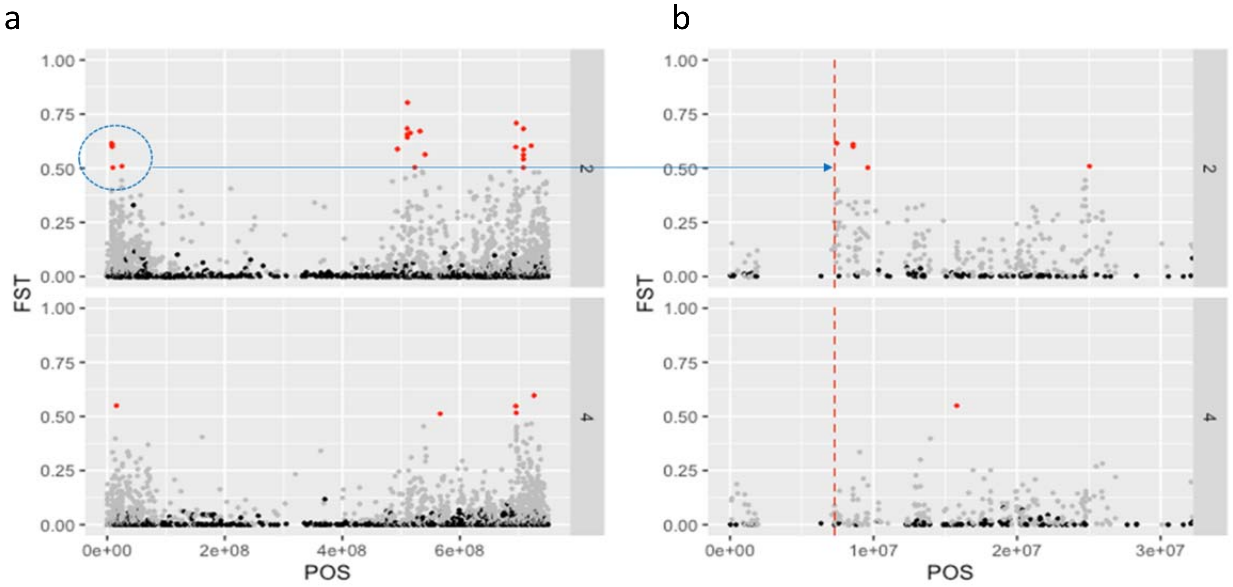

Figure 4: Analysis of Fst values on a variant-per-variant basis across the genome highlights areas of positive selection. a) Fst analysis of the complete chromosome 3A in cluster 2 (upper half) and 4 (lower half of figures); b) Zoom-in of chromosome 3A positioning of the pre-harvest sprouting gene *TaMFT*.

GWAS analysis reveals loci associated with grain protein content and SDS-sedimentation

To conduct association scans with the DArTseq data, we phenotyped 3,870 samples for two important traits for processing and end-use quality, grain protein content (GPC) and SDS-sedimentation (Fig. 5 and Table S5). We found 18 genomic regions associated with GPC on 12 chromosomes, with highest peaks on 4A and 4B, followed by 5A, 5B, 7A and 7B. Similarly, Kumar et al (2019)[46] reported major and stable QTL for GPC on chromosomes 5B, 7A and 7B of an exotic genotype and indicated that these QTL were independent of grain yield. Such QTL could be useful to enhance GPC through marker-assisted selection, particularly if they do not compromise yield. Comparison with 49 GPC studies[47] suggests that QGPC.ndsu.5B (located on 5BS) and QGPC.ndsu.7A.2 (located on 7AL) could be novel QTL which the exotic germplasm could contribute to the wheat breeding gene pool for increasing GPC.

SDS-sedimentation is a common test to determine overall gluten quality. High values on this test are associated with strong gluten (preferred for bread-making), while low values are associated with weak gluten (preferred for pastry products). Here we report significant QTL for SDS-sedimentation and putatively associate them with known storage protein genes. Specifically, high molecular weight glutenins, Glu-A1, Glu-B1 and Glu-D1 (located on the long arms of chromosomes 1A, 1B and 1D), and low molecular weight glutenins, Glu-A3, Glu-B3 and Glu-D3 (located on the short arms of chromosomes 1A, 1B and 1D) are candidate genes for the QTL on chromosomes 1A, 1B and 1D, which had the largest effects on SDS-sedimentation in this study. All these glutenin genes are well known and their variability and effects on processing and end-use quality have been extensively reported[48].

a

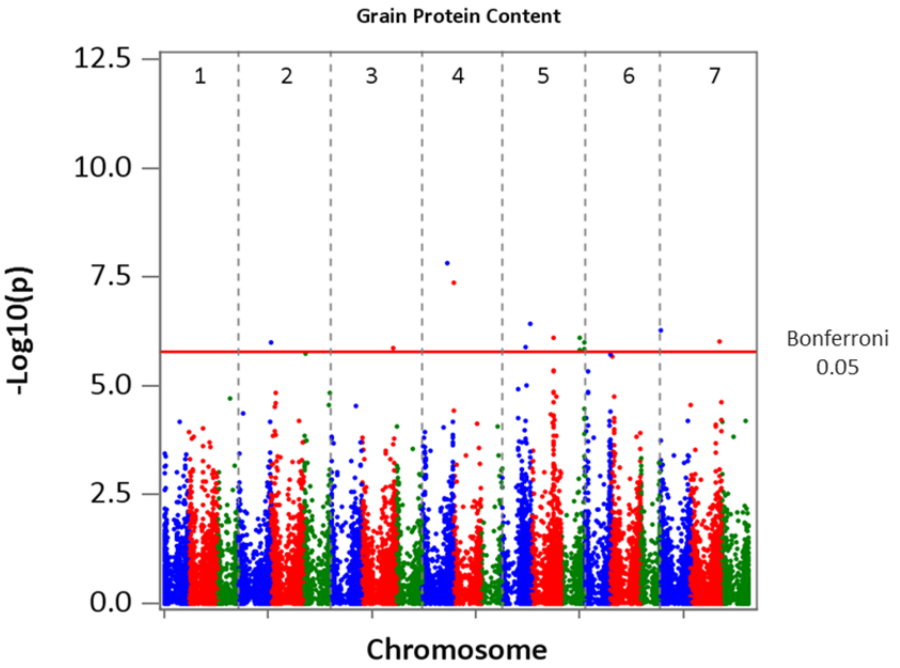

b

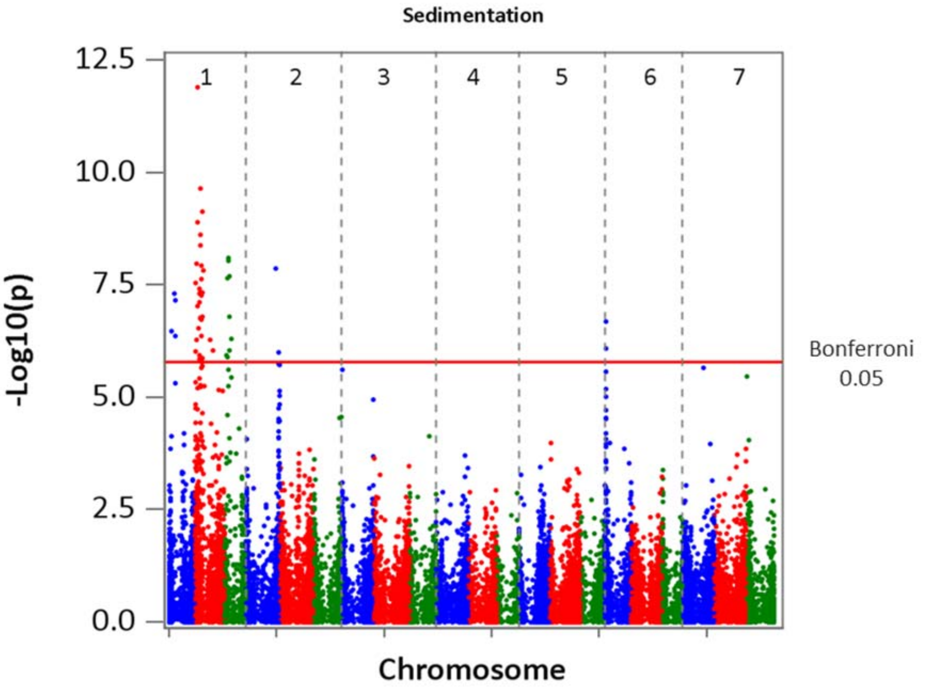

Figure 5: Genome-wide association scans for wheat quality characters. a) 18 genomic regions were associated with GPC on 12 chromosomes, with the highest peaks on 4A and 4B, followed

by 5A, 5B, 7A and 7B. b) 19 genomic regions associated with SDS on 4 chromosomes, 1A, 1B and 1D, previously reported, and a new QTL on 2A.

(i) In the discussion section **(line 574)** we incorporated many examples of how this data has been already utilized in different breeding studies:

These massive-scale genotypic data have already been used in several studies focused on enhancing the use of genetic diversity in wheat breeding. Singh et al.[28] used DArTseq genotypic and multi-environment phenotypic data to demonstrate, for the first time, positive contributions of exotic germplasm to lines derived from crosses of exotics with CIMMYT's best elite lines. Genomic-based prediction using 8,416 Mexican and 2,403 Iranian landraces from CIMMYT's germplasm bank estimated prediction accuracies from 0.41 to 0.65 for Mexican, and from 0.18 to 0.65 for Iranian landraces[32]. Saint Pierre et al.[49] characterized 803 spring wheat lines, including elite germplasm and diverse accessions, to develop models for genomic prediction of phenology traits and grain yield, and to predict performance of lines in environments where the lines were not tested. Sehgal et al.[50] selected 200 diverse gene bank accessions out of 1,423 spring bread wheat accessions for use in pre-breeding and allele mining for candidate genes for drought and heat stress tolerance. Finally, Sehgal et al.[51] described efforts to identify genomic regions with stable expression and their epistatic interactions for grain yield and yield stability in a large panel of elite wheat under multiple environments via a genome wide association mapping (GWAM) approach. These multiple studies exemplify the value of this germplasm which is now easier to utilize and exploit thanks to the resources generated in the present study.

(line 593) Native allelic variation for relevant breeding traits is one such resource. The analysis provides a basis for targeted exploration and allele mining activities moving forward. Diversity per-se is of limited value for breeding, instead the value lies in the understanding of diversity and the identification and use of novel diversity associated with breeder relevant traits. There are a number of paradigms currently in use to better understand and identify breeder relevant diversity. Before the advent of wide-spread genomic characterization core collections were proposed as a model for mining representations of general diversity, these have evolved to use genomic data in their definition as more widespread characterization has become available52–54. Another approach, reflecting landrace adaptation to local environments, was the Focused Identification of Germplasm Strategy (FIGS) where passport derived collection site variables were used to identify materials of potential interest for phenotypic evaluation for specific environment-associated traits. More recent analysis has extended and revised these approaches to incorporate in-depth understanding and application of genomics. In maize, passport data, associated climate variables from collection sites are being used in conjunction with genome wide fingerprint data to identify alleles from broad germplasm collections associated with breeder relevant parameters[56,57]. Using this information and screening against genomic profiles of existing elite germplasm enables the identification of both previously un-

highlighted standing variation of breeding relevance existing within elite germplasm and also novel breeder relevant diversity which can then be introgressed into breeding pools using appropriate strategies (S. Hearne pers comm). Taking these parallels and moving forward with wheat, there is a clear opportunity to use the understandings derived from comprehensive genomic characterization, together with associated data, to define and implement clear strategies to explore and use relevant genetic diversity for breeding in a more targeted data driven manner.

(i) We welcome the comment about perceived repetitiveness of the results section. In order to address the issue, we have adjusted titles and some of the flow within the respective hexaploid, tetraploid and CWR results sub-sections. Nonetheless, we still describe a common set of measures for each analysis group for scientific clarity, enabling those interested in cross-group overviews to compare and contrast the genepool level differences.

I leave it up to the editor to decide to what extend these two points should be addressed. All the 'technical' comments have been addressed.

As mentioned in my first report, this is to my knowledge the largest genotyping effort in plant science, which justifies publication of this article in Nature Communications.

Reviewer #2 (Remarks to the Author):

Some of my comments haven't been adressed and for some others the response is somewhat cryptic and I'm not sure whether the authors agree and have amended the ms. or not. Can you please clarify these? Also it would be helpful to have a ms. version that highlights the changes undertaken.

First revision: Reviewer #2 (Remarks to the Author):

Sansaloni et al., Dissecting wheat biodiversity to ensure bread for future generations

Sansaloni et al embark towards the characterisation, analysis and ordering of wheat genebank accessions. The Dart approach used allows to skim almost 80000 (!!!!!!) wheat accessions in the two most important germplasm repositories worldwide in an economic manner. The approach and aim is fairly similar to a recent report on the characterisation and analysis of the barley germplasm (PMID:31253974). (Btw: this should be mentioned and cited; nop I'm not an author of this paper). Breadth of the analysis, the huge collections analysed and structured and the sheer economic, scientific and socioeconomic importance of wheat underpin the high priority of structuring, analysing and exploiting the germplasm collections using powerful NGS and data analysis approaches for very practical and urgent needs we have. A very important and valuable contribution for next generation breeding and structuring/exploitation of our germplasm resources! Nevertheless, I have a couple of points I have to

mention and cause me some difficulties in understanding:

Lines 181 ff: Fst values are being calculated in a series of different figures. It (1) wouldn't harm to introduce the concept and question addressed by the Fst values as well as the meaning/assumption of th thresholds used. (2) It is unusual and without any value to calculate chromosome scale Fst's. Usually Fst's are computed for sliding windows and drops or steep increases demarcate potential selection. Complementary measures are often discussed and used in parallel as well. Since the sequences are based on Dart approach and technology the resulting sequence information doesn't deliver longer continuous sequences. Is it valid to use the short (77bp, correct?) sequence reads for Fst calculations? If so, I'd like to see sliding windows on selected chromosomes that ideally match and are confirmed by previously reported Fst profiles that were based on whole genome profiles (eg.: PMID:3096261). Avoid the Fst values computed for whole chromosomes.

From the text: Calculation of the fixation index (Fst) on a per marker basis and the average value on a 1Mb window identified regions in the genome where the population structure explains a high proportion of the genetic diversity. This analysis suggest there are different levels of allelic fixation between the sub-groups defined above.

Figures: some of the figures are direct output of the programs used for the respective analysis and are more or less screen shot quality. Can this be amended? In some of the figures one or the other axes is simply missing. Also for all of the "C" figures (bar chart type diversity analysis) This is really enigmatic and hard to grasp/non-intuitive. The figure legends don't give sufficient information, abbreviations used are not explained (I can guess though…) and why in some of the fields numbers are given and in other not is unclear

We increased the quality of the figures and we made sure all axes are present. We moved the figure C to supplementary figures since was not intuitive and we replaced for an STRUCTURE analysis/figure which is more standard way of present the diversity analysis in many publications. In the supplementary figure (bar chart type) we only show the number in the 2 group that are part of the division, not in the other because will be redundant.

Btw: what are these numbers?

The number inside the boxes are the He (again I can guess, but…).

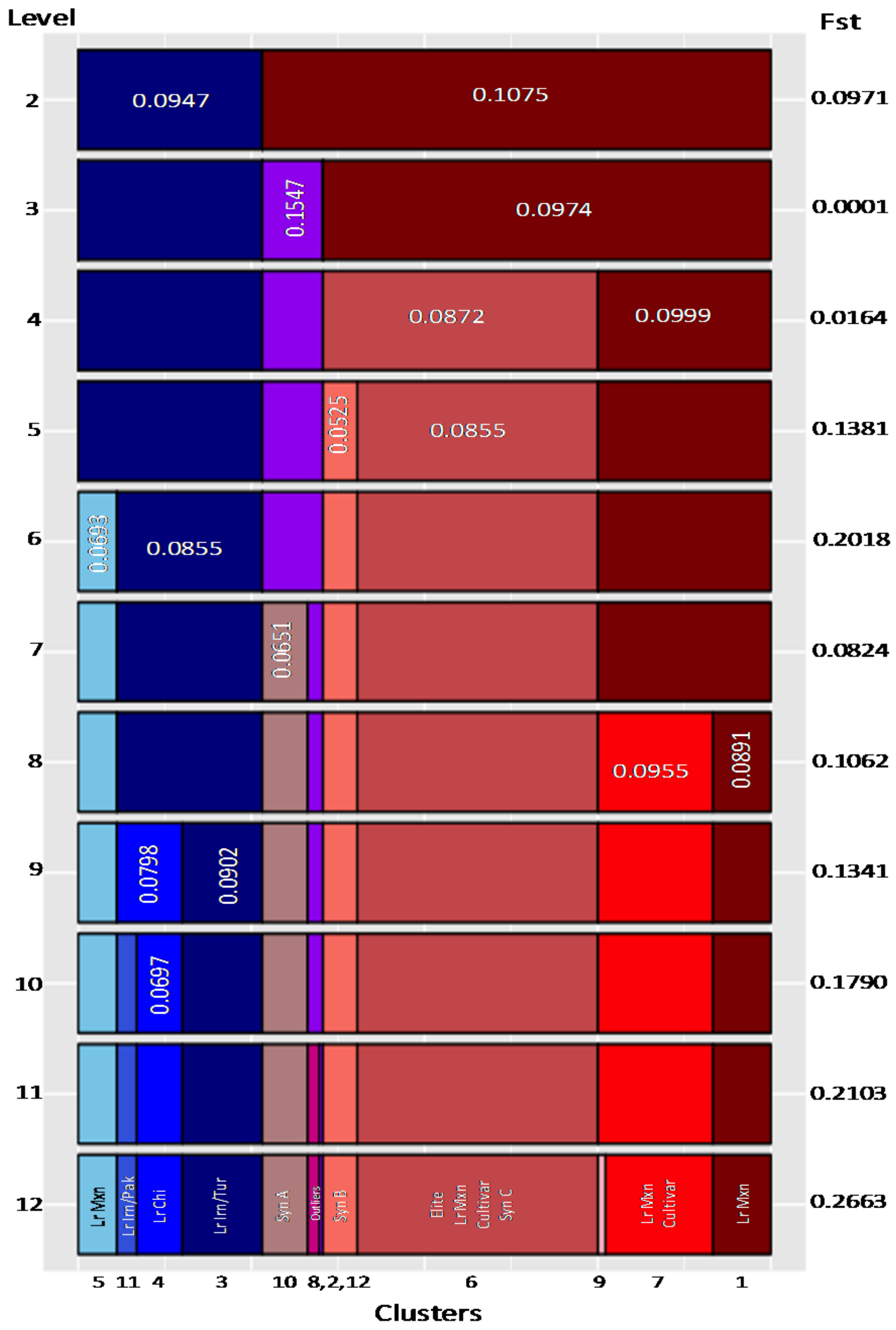

Supplementary Figure 7: Representation of the distribution of 12 groups based on clusters analysis. The size of the boxes are proportional to the number of accession. In the right side are the Fixation index (Fst) values and inside the boxes the expected heterozygosity (He) of each group division and in the bottom the 12 clusters are identified with a brief description and the number which corresponds to figure 1B. Left numbers are the number of levels.

As already spelled out above: I don't think that given whole genome/-chromosome Fst values is a valid way to make use of the Fst analysis. I'd be grateful for a modification.

We modified this in the new analysis. The analysis of Fst is on per marker bases and it was previously on sliding windows.

Some (minor) comments and criticism:
Line 66: "…linkage drag, resulting from the numerous undesired or deleterious genes …" well the linkage drag is not a consequence of introduction of undesired genes as suggested in this sentence. Also I'd be very sceptical about the concept of introduction of deleterious genes. Less favourable genes or alleles yes, but I'm not sure whether any case of a deleterious gene introduction (in a molecular and mechanistic rather than genetic sense) has been demonstrated. Can you modify sentence and argument?

Several challenges limit breeders' use of germplasm bank accessions, but the biggest hurdles are 1) identifying which of the possible 560,000 accessions to use, and 2) the co-introduction of less favorable alleles when landraces and CWR are crossed with elite lines.

Line 101: you might ant references to more recent large scale/genomic reports that also report on gene flow and introgressions among different wheat species. PMID:31043759, PMID:31043760

Modified in the text.

Lines 122-125: The percentage of SNP markers with genetic/genomic positions is fairly low given full genome reference genomes. Why is this? Ambigious mappings because of short sequence length?

The percentage of markers with genome position is fairly low because in the diversity analysis we included many different species (8 for the hexaploidy, 8 for the tetraploid and 29 for the CWR). If we consider that the reference genome represents a very homogeneous T. Aestivum aestivum specie, it is expected that not all markers generated will aligned on the reference. This observation doesn't mean that are not good markers, simply are not present in the reference.

Line 134: What are "Fst values on a per marker basis"? Please clearify. What is sequence window chosen?

From the text: Calculation of the fixation index (Fst) on a per marker basis and the average value on a 1Mb window identified regions in the genome where the population structure explains a high proportion of the genetic diversity. This analysis suggest there are different levels of allelic fixation between the sub-groups defined above.

Line 201: "…likely tetraploids…" can this be tested and confirmed?

Most of accessions identified as outliers were visually tested and we confirmed that were tetraploid (Durum wheat). Some of the once that we still have doubt we will plant in the field for further evaluation.

Figure 1E: what is cluster 3? And is 3D and 4D really significantly different to some of the other D chromosomes?

Cluster 3 is the level in which the clustering analysis is divided in 3 groups. The synthetic wheat group (purple) is differentiated from the Elite/landrace group (red). It is expected that the D genome will be significantly different between the groups since the synthetic are created from a tetraploid wheat with an exotic D genome (Aegilop taichii).

Part of figure 1C:

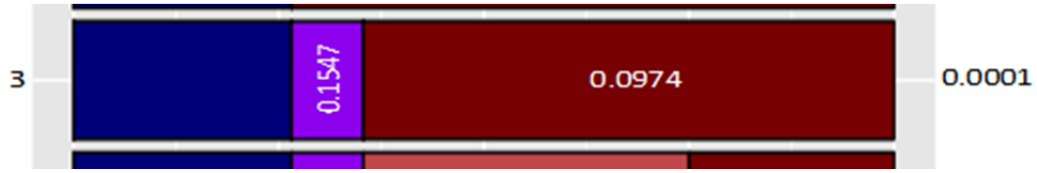

In general: can you please report only one digit after the comma (e.g. 2,5 instead of 2,53)? Would improve readability…

Agree and modified in the text.

Line 311/312: "The third division…" This an enigmatic sentence. Can you amend and translate into less population genetics/genomics terms? The same is true for Figure 2 legend. Second division of three clusters… ????

Agree and modified in the text.

Line 374: involved rather than involve

Agree and modified in the text.

.

Line 318/319: 2E is supposed to show that 1B and 7B provide outstanding Fst values/diversity. I'm not convinced about this argument when checking the plot. Any significance measures?

Agree and modified in the text.

Reviewer #3 (Remarks to the Author):

The authors have addressed most of the concerns and questions identified in the reviewers. However, both I and another reviewer have raised the issue of the relevance of this work to wheat breeding programs and suggest the authors provide some examples of how these datasets could be used. The authors have provided the examples of the pre-harvest sprouting gene, and the traits grain protein content and SDS-sedimentation. In addition, they provide several examples of how genotypic data on diversity panels have been used (lines 576 to 591) but these are all examples of previous studies using other datasets. If anything, these examples suggest the large dataset generated here is not required for these types of analyses. The authors should explain why this dataset adds value over and above that provided by smaller studies. Also, given that the title, abstract and introduction all make a big point of the utility of the data in breeding, the relevance to breeding requires further development.

Thanks for your comments.

All examples given in the discussion (line 576 to 591) are studies performed with small subsets that are belong to the large data presented in this study. This is a way to demonstrate the importance of this analysis since many other studies can utilized this information for different purpose and applications like genomic selection, GWAS, specific diversity studies by country, region or species.

We added to the new version of the discussion:

(line 593) Native allelic variation for relevant breeding traits is one such resource. The analysis provides a basis for targeted exploration and allele mining activities moving forward. Diversity per-se is of limited value for breeding, instead the value lies in the understanding of diversity and the identification and use of novel diversity associated with breeder relevant traits. There are a number of paradigms currently in use to better understand and identify breeder relevant diversity. Before the advent of wide-spread genomic characterization core collections were proposed as a model for mining representations of general diversity, these have evolved to use genomic data in their definition as more widespread characterization has become available52–54. Another approach, reflecting landrace adaptation to local environments, was the Focused Identification of Germplasm Strategy (FIGS) where passport derived collection site variables were used to identify materials of potential interest for phenotypic evaluation for specific environment-associated traits. More recent analysis has extended and revised these approaches to incorporate in-depth understanding and application of genomics. In maize, passport data, associated climate variables from collection sites are being used in conjunction with genome wide fingerprint data to identify alleles from broad germplasm collections associated with breeder relevant parameters[56,57]. Using this information and screening against genomic profiles of existing elite germplasm enables the identification of both previously un-highlighted standing variation of breeding relevance existing within elite germplasm and also novel breeder relevant diversity which can then be introgressed into breeding pools using appropriate strategies (S. Hearne pers comm). Taking these parallels and moving forward with wheat, there is a clear opportunity to use the understandings derived from comprehensive genomic characterization, together with associated data, to define and implement clear

strategies to explore and use relevant genetic diversity for breeding in a more targeted data driven manner.

The authors have also not effectively dealt with the concerns raised by two reviewers around the use of DArTseq as the genotyping platform. The comment that “DArTseq has become a technology of choice for practically all areas of research….” is not correct and does not address the concerns raised. I don’t have a problem with the use of this technique, but the authors cannot ignore the intrinsic limitations of this approach compared to other techniques. Somewhere in the manuscript they need to provide comment and an explanation for why this platform was used.

The genotyping work presented in this manuscript began in 2011 when the project Seeds of discovery started. So, it was almost 10 years ago. The best option of genotyping at that moment were GBS using ApeK enzyme (which is not ideal for wheat due to the large genome) and DArTseq that use methylation filtration (PstI) to reduce the complexity of the genome representation. Furthermore, the allele-calling pipeline does not require a reference genome, which was important to start the work and is still important to avoid ascertainment bias, which would be very strong, considering that in this study different species are compared. The reference-free approach offers an unbiased method to assess genetic diversity in a large collection of accessions as the one we have analyzed. The use of any of the current available high-quality wheat references would introduce an unbalanced view of the present diversity disregarding, for instance, novel genomic sequences that are only presented in exotic accessions.

We included a comment into the Method section:

In this technology, the allele-calling pipeline does not require a reference genome which offers an unbiased method to assess genetic diversity in a large collection of accessions as the one we have analyzed. It might not be the most suitable approach for other investigations in which having a free-reference calling or not using a fully repeatable method like a chip or array it could be a disadvantage. But, considering the objectives of this study and the exotic material we are analysing we found that DArTseq was the most appropriate genotyping approached to use at the beginning of the Seeds of Discovery project.

REVIEWERS' COMMENTS:

Reviewer #1 (Remarks to the Author):

In this revised version of the manuscript, the authors include substantial new data that demonstrate the usefulness of this large genotyping effort for trait discovery and breeding. The Fst analysis reveals potential genomic regions under selection. The genome-wide association study revealed candidate loci for grain protein content and other quality traits. Most importantly, the authors highlight the potential role of exotic material that might contribute new beneficial alleles in future wheat breeding efforts.

Regarding the discussion around the DArTseq technology. From today's perspective, DArTseq is probably not the best choice. But as the authors mention in their response letter, this was a long-lasting project and I appreciate that at the time when this project started this technology was among the best for wheat genotyping.

I am satisfied with this new version of the manuscript and recommend publication in Nature Communications.

Reviewer #2 (Remarks to the Author):

all my previously raised points have been adressed and answered. Thanks! A very important contribution

Reviewer #3 (Remarks to the Author):

In the second review of this paper, I raised two concerns with the revised version. Unfortunately, neither have been addressed in the latest revision.
The first concern was around the added value to wheat breeding provided by this extensive study relative to previous work on characterising germplasm collections. The authors have provided a good discussion (from line 553) around the significance of diversity in breeding and comment on a range of approaches for assessing germplasm. However, their argument about the added value provided through this new dataset is based on diversity "which can then be introgressed into breeding pools using appropriate strategies (S. Hearne pers comm)." This is hardy an adequate response to the concern raised. The authors fail to make a case for why this study will have a major impact on wheat breeding strategies.
The second concern was around the use of DArTseq for genotyping. The authors were asked to provide a commentary on the limitations of this technique. They have failed to do so. The two sentences provided (lines 608 to 612) are incomprehensible. The comment made in the response letter and in the sentence (lines 612 to 614), that the project was initiated in 2011 and this was seen as an appropriate technology at the time, is reasonable. However, this is also a tacit recognition that an alternative method would be used if the project were to be initiated today. Consequently, a discussion of the weaknesses and limitations is still needed.

REVIEWERS' COMMENTS:

Reviewer #1 (Remarks to the Author):

In this revised version of the manuscript, the authors include substantial new data that demonstrate the usefulness of this large genotyping effort for trait discovery and breeding. The Fst analysis reveals potential genomic regions under selection. The genome-wide association study revealed candidate loci for grain protein content and other quality traits. Most importantly, the authors highlight the potential role of exotic material that might contribute new beneficial alleles in future wheat breeding efforts.

Regarding the discussion around the DArTseq technology. From today's perspective, DArTseq is probably not the best choice. But as the authors mention in their response letter, this was a long-lasting project and I appreciate that at the time when this project started this technology was among the best for wheat genotyping.

I am satisfied with this new version of the manuscript and recommend publication in Nature Communications.

Thank you for your positives comments and the contributions to improve this manuscript.

Reviewer #2 (Remarks to the Author):

all my previously raised points have been adressed and answered. Thanks! A very important contribution

Thank you for all suggestions. It really help us to improve the manuscript.

Reviewer #3 (Remarks to the Author):

In the second review of this paper, I raised two concerns with the revised version. Unfortunately, neither have been addressed in the latest revision.
The first concern was around the added value to wheat breeding provided by this extensive study relative to previous work on characterising germplasm collections. The authors have provided a good discussion (from line 553) around the significance of diversity in breeding and comment on a range of approaches for assessing germplasm. However, their argument about the added value provided through this new dataset is based on diversity "which can then be introgressed into breeding pools using appropriate strategies (S. Hearne pers comm)." This is hardy an adequate response to the concern raised. The authors fail to make a case for why this study will have a major impact on wheat breeding strategies.
The second concern was around the use of DArTseq for genotyping. The authors were asked to provide a commentary on the limitations of this technique. They have failed to do so. The two

sentences provided (lines 608 to 612) are incomprehensible. The comment made in the response letter and in the sentence (lines 612 to 614), that the project was initiated in 2011 and this was seen as an appropriate technology at the time, is reasonable. However, this is also a tacit recognition that an alternative method would be used if the project were to be initiated today. Consequently, a discussion of the weaknesses and limitations is still needed.

Thank you for all your comments and suggestions. In the previous version I added in the method section the limitations and an explanation of why this technology was selected 10 years ego.
